# Determination of hepatitis C virus subtype prevalent in Sindh, Pakistan: a phylogenetic analysis

**DOI:** 10.1038/s41598-024-59342-7

**Published:** 2024-05-15

**Authors:** Saba Farooq, Sirmast Faiz, Atia-tul Wahab, M. Iqbal Choudhary

**Affiliations:** 1 Mediagnost Gesellschaft Für Forschung Und Herstellung Von Diagnostika, Reutlingen, Germany; 2 National Institute of Virology, Dr. Panjwani Center for Molecular Medicine and Drug Research, International Center of Chemical and Biological Sciences, University of Karachi, Karachi, 75270 Pakistan; 3 Dr. Panjwani Center for Molecular Medicine and Drug Research, International Center of Chemical and Biological Sciences, University of Karachi, Karachi, 75270 Pakistan; 4 H. E. J. Research Institute of Chemistry, International Center for Chemical and Biological Sciences, University of Karachi, Karachi, 75270 Pakistan; 5 Department of Biochemistry, Faculty of Science, King Abdulaziz University, 21412 Jeddah, Saudi Arabia

**Keywords:** Hepatitis, Hepatitis C virus, Prevalence, Genotype, Phylogenetic analysis, Genetic diversity, Hepatitis C virus, Viral epidemiology, Genotype

## Abstract

Hepatitis is a major public health issue, affecting 10–17 million people worldwide, with its prevalence continuously increasing. The Hepatitis C virus (HCV) is responsible for liver related diseases, which include liver cirrhosis, hepatocellular carcinoma, and chronic hepatitis. Pakistan is experiencing a serious rise in HCV cases. This study aimed to assess the prevalence and distribution of HCV genotypes in Sindh, Pakistan. Serum samples from HCV-positive patients were collected from various local hospitals in Sindh. These samples were first screened for HCV antibodies using ELISA. Samples that tested positive for HCV RNA underwent further genotyping through sequencing using the standard Sanger method. The genotypes were identified by comparing the sequences with those available in the National Center for Biotechnology Information (NCBI) database, and a phylogenetic tree was constructed. The phylogenetic analysis showed that all isolates in this study were clustered with genotypes 3a and 3b, except for one sequence that was clustered with genotype 1a. No isolates were found to be clustered with reference genomes of genotypes 2, 4, 5, 6, and 7 suggesting that genotype 3a is endemic in this region. The analyzed sequences demonstrated a 98% similarity with reference and isolated sequences. In summary, sequencing of the HCV 5′ UTR essential for identifying the predominant genotype of HCV RNA in the Sindh region Further research on the distribution of HCV genotypes in other regions of Pakistan could aid in improving screening processes, identifying more effective treatment options, and developing suitable prevention strategies.

## Introduction

The Hepatitis C virus (HCV) belonging to the Flaviviridae family is a hepatotropic virus characterized by its enveloped structure and positive-stranded RNA^1,2^. Often asymptomatic HCV infection can become chronic, leading to liver cirrhosis in 20% cases and increasing the risk of hepatocellular carcinoma (HCC), a major cause of liver-related deaths worldwide^3–5^.The prevalence of HCV-associated cirrhosis is on the rise. HCV transmission occurs through exposure to infected blood, blood products, and body fluids, with invasive procedures, and intravenous drug use also posing transmission risks^6^. Globally, over 3% of the population is chronically infected with HCV, underscoring the urgent need for anti-viral and vaccines^7,8^. Recently WHO received officially validated country-provided data from 130 countries or territories, and used partner-provided data for 70 countries or territories. It was estimated that in 2019, globally, 295·9 million (3·8%) people were living with chronic hepatitis B virus (HBV) infection and 57·8 million (0·8%) people were living with chronic hepatitis C virus (HCV) infection. Globally, there were more than 3·0 million new infections with HBV and HCV and more than 1·1 million deaths due to the viruses in 2019. Among people with HCV infection, 15·2 million (95% CI 12·1–19·0) had been diagnosed between 2015 and 2019, and 9·4 million (7·5–11·7) people diagnosed with hepatitis C infection were treated with direct-acting antiviral drugs between 2015 and 2019^9^. One of the crucial public health needs is the rapid and accurate diagnosis of HCV infections, especially in the high-risk patients, such as those undergoing hemodialysis, blood donors, etc. Definitive diagnosis of HCV-related infections is complicated, and treatment is highly expensive with severe adverse effects. Typically, diagnosis involves the detection of HCV antigen-specific antibodies and viral RNA or HCV core antigens are used^10,11^ Among different diagnostic methods for the detection of HCV, PCR is considered to be the most accurate, and reliable method^12^.

Rapid and accurate genotyping and subtyping identification are crucial from a diagnostic standpoint, as recent treatment strategies are genotype-dependent^13^. The recommended methods for HCV genotyping include the sequencing and phylogenetic analysis. Another method is INNO-LiPA, which targets the highly conserved 5′ untranslated region (UTR) and core region of the virus using genotype- and subtype-specific probes^14^^.^ However, sometimes INNO-LiPA is not able to fully resolve the subtype, and the further refinement may be required by using Sanger sequencing^15,16^.

The incidence of HCV remains high in some regions of the world. During the last 10 years, major progress has been made in terms of HCV characterization and understanding of its genetic diversity^17^. Along with this the development of the third-generation based antibody-based diagnostic tests were developed for most clinical settings. The understanding of genotyping of HCV is important for the treatment because every genotype is associated with variable clinical outcomes^18^.

Study of the geographical distribution worldwide played an important role in the identification of prevalence of most predominant genotypes of HCV. So far, 3 major and broad genotypic patterns have been identified. Pattern one is characterized by high genetic diversity and involves the geographically discrete areas with different genotypes, such as in West Africa the most predominant types are 1, and 2, Central Africa with type 4, and Asia with types 3, and 6. According to a study based on the small genotypes 4 (GT 4) cohort, the most predominant genotype in Middle East region was observed as genotype 4^19^. However, HCV genotype 1 is the most prevalent among non-Arab countries in the region including Turkey, Iran, Cyprus, and Israel^20^.The genotype 6 is mainly distributed in southeast Asia and southern China^21^. This pattern of distribution is due to the long period of endemic infections in particular regions. Second pattern represents the areas with a few subtypes which are circulating in specific risk groups, such as subtype 3a in drug addicts^22^. While the third pattern exists in the region where only single subtype is predominant, for instance in Egypt subtype 4a, and in South Africa subtype 5a is prevalent^23^ According to a phylogenetic study, HCV has existed in human hosts for thousands of years, resulting in particular genotypes that are endemic in distinct geographical regions. Genotypic classification also provides information in terms of global viral evolution and epidemiology and helps in predicting the response to interferon (IFN)-based treatment regimens^24^.

HCV genotype is the strongest predicting factor for sustained virological response because infected individuals with diverse genotypes respond differently to alpha-interferon therapy. In addition, Genotypic determination of HCV is instrumental for controlling therapeutic regimen, improving local control programs and eventually producing an effective vaccine^18^. Only a few studies were conducted in Pakistan on the distribution of different genotypes of HCV.

Patients with Hepatocellular carcinoma (HCC) display strong relevance with genotype 3a, suggesting an association of chronic HCV infection with genotype 3a and HCC. This also reflects the increased rate of oncogenicity^25^. Direct sequencing of the 5ʹ UTR is a valuable method for clinical detection of different HCV genotypes^26^. The current study was conducted to assess the identification, confirmation, and characterization of most predominant genotype of HCV in Sindh province. The present study provides the preliminary information regarding the prevalence of HCV in the province of Sindh.

## Materials and methods

### Ethical approval

The samples used in the study were taken after the approval of the ethical committees from the respective laboratories. This study was approved by the Institutional Ethical Committee (IEC) of the International Center for Chemical and Biological Sciences (ICCBS), University of Karachi (Study #: -059-HB-2021, Protocol #: ICCBS/IEC- 059-HB-2021/Protocol/3.0, Version #: 3.0). This study was conducted in accordance with the principles of the Declaration of Helsinki. 2 mL peripheral blood samples from the recruited patients were collected at different hospitals of Sindh, Pakistan. All study participants provided written informed consent.

### Collection of serum samples

Samples were collected from different diagnostic laboratories/hospitals of Sindh. 2 mL of blood was collected in a clot activator gel tube. Samples were centrifuged at 3000 RPM for 5 min to obtain clear serum. The serum was aliquoted/transferred in a 2 mL serum cup and kept at − 20 °C until further analysis.

### Anti-HCV evaluation

For the rapid screening of HCV infections, the anti‐HCV assays are widely used^27^. During the study, all samples were used for evaluation of anti-HCV antibodies by commercially available kit HCV ELISA 4.0 (MP Biomedicals Germany, GmbH) according to manufacturer’s instructions. Briefly, diluted samples or controls were loaded into a 96-well plate, pre-coated with a HCV-specific antigen. Biotin-conjugate was added in each well, except in blank. The plate was then incubated for 60 min at 37 °C. After incubation, the plate was washed with the dilution-washing buffer, and HRP-conjugate was added, and the plate was incubated for 30 min at 37 °C. After incubation, plate was washed, and coloring solutions (chromagen A and B) were added for detection. Finally, the reaction was stopped by adding stop solution. Intense yellow color indicated the presence of HCV antibodies. Colorimetric signal was measured by absorbance at 450 nm by using a spectrophotometer.

### Nucleic acid preparation, reverse transcription, and PCR

For more sensitive and accurate diagnosis of infection, and the assessment of virological responses, the PCR based methods are principally recommended. It plays a key role in identification, quantification and characterization of the components of HCV viral particles, such as HCV RNA^28^. During the research, nucleic acids from 200 μL serum samples were extracted by the using the viral nucleic acid DNA/ RNA extraction kit (Nucleospin virus, Macherey Nagel, GmbH and Co, Germany), following the manufacturer’s instructions. HCV DETECT (Mediagnost Diagnostika GmbH, Reutlingen; Germany) was used for PCR analysis. For one-step HCV Reverse Transcriptase PCR (RT-PCR), an enzyme mix (Luna) and the primer mix were used. After reverse transcription of the extracted HCV-RNA, the specific amplification of the cDNA yielded a 322 bp PCR product. The cDNA was used as a template for PCR amplification. For the reverse transcription, the reaction mixture was subjected to a temperature of 55 °C for 15 min. Further reaction cycle involved initial denaturation at 95 °C for 1 min, followed by denaturation at 95 °C for 10 secs (40 cycles) and extension at 60 °C for 1 min (40 cycles). Samples from the reaction mixture were subjected to electrophoresis on 2% agarose gel (BioRad). The size of the HCV-PCR product was determined by comparing with a 100-bp DNA ladder (Fermentas, USA), used as DNA size marker. DNA on gel were stained with gel red, and the gels were visualized in a BioRad Gel Documentation System for observation.

### Primers for PCR and sequencing

Primer no. P3 (5′-CACTCCCCTGTGAGGAACT 3′; sense), and no. P9 (5′ CTCCAGAGCATCTGGCA CG -3′; antisense) were used as RT-PCR primers specific for 322 bp. While for sequencing, GGGGCACTCGCAAGC was used.

### Purification of PCR products and sequencing

PCR products of the 5′ UTR region was purified using agarose gel-centrifugation protocol from Omega Bio-Tek (Product D 6492-01/ D 6493-01) and Thermo Scientific GeneJET PCR purification Kit (K0701) by using the manufacturers’ protocols. The genotype of each sample was determined by comparing its sequence with those of HCV prototypes deposited in the GenBank database, followed by further genetic analysis. Purified PCR products were sequenced at 4base Lab AG, Reutlingen, Germany, on a Genetic Analyzer 3130 × 1 using BigDye Terminator 3.1 RR Chemistry.

### Interpretation of HCV 3a nucleotide sequences and phylogenetic analysis

The sequencing data of 5’UTR of HCV-positive patients were analyzed using BioEdit 7.2 software((http://bioedit.software.informer.com/)). The ambiguous data and wrong peaks were removed. Multiple sequence alignment of 37 sequences and reference genomes of each genotype was performed using muscle alignment tool^29^. The aligned data was viewed by Aliview^30^. The nucleotide similarity of the 5ʹ-UTR sequence was checked by using multiple alignment fast Fourier transform methods^31^ (MAFFT version 7 software). The phylogenetic tree was constructed by RAxML 8.2.12 tool by using the maximum likelihood (ML) method and 1000 bootstrap replicates^32^. The phylogeny tree was further elaborated with FigTree v.1.4.434 (http://tree.bio.ed.ac.uk/software/figtree/) and MEGA X software35^33–36^.

### Ethical approval

Ethical clearance was obtained from the research department.

## Results and discussion

### Study population characteristics

In the present study, HCV samples (n = 96) were collected based on their demographic variables. Among the collected samples, 55 (57%) were identified as HCV-positive among females, while 41 (43%) were identified among males. This gender-specific distribution of HCV positivity forms the basis for further analysis related to hepatitis C in studied samples cohort.

The slight increase in HCV prevalence in females as compared to males was observed but this difference was not significant. In the chi-squared test for given probabilities, a chi-squared statistic of 2.0417 with 1 degree of freedom and a *p*-value of 0.153 were obtained. The results suggested that there is no significant difference in the distribution of HCV in both genders in the studied samples.

**Figure1:**
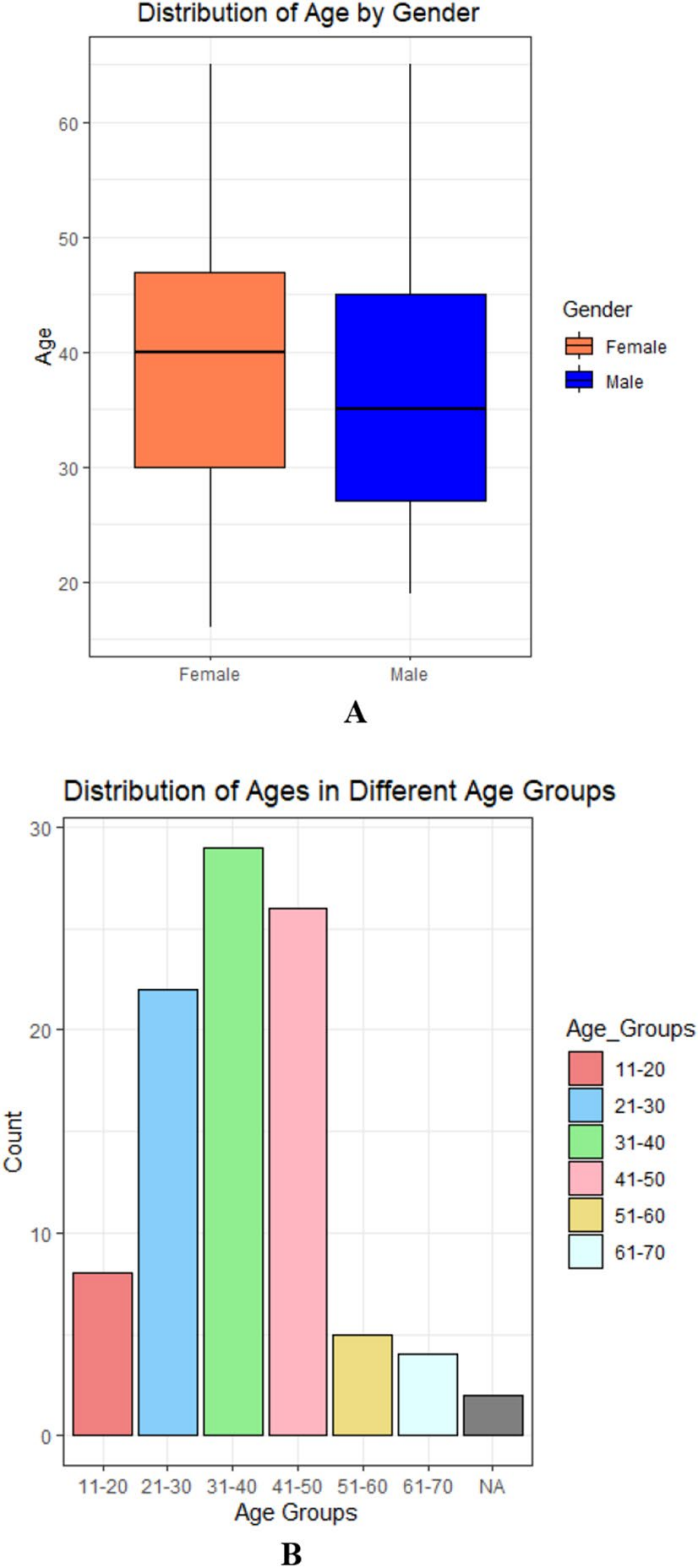
(**A**–**B**): The gender and age association between two groups, indicating the non-significant differences.

The mean age of the study participants was 37.97 years, with a median age of 36.5 years (Fig. 1). The standard deviation of 11.72 years indicated moderate variability in ages within the sample. The mean age in the female patients was 38.76 years, while the mean age in the male patients was 36.90 years, confirming the non-significant and slight age difference between the two groups. The box plot indicates the distribution of age by gender. The Welch Two Sample t-test was conducted to compare the mean ages between the female patients and male patients’ groups. The results showed a t-value of 0.769 with 86.784 degrees of freedom and a *p-*value of 0.444. The 95% confidence interval for the difference in means was [-2.949539, 6.671934]. The lack of statistical significance (p-value > 0.05) and the confidence interval that includes zero suggest that p-value > 0.05 suggests that there is no significant difference in mean ages between female and male patients in our study.

The bar graph presents the prevalence of HCV within different age groups in our study population. Notably, varying prevalence rates across age categories were observed. Individuals in the age group 31–40 exhibited the highest prevalence of HCV (30.2%), followed by age groups 21–30 (22.9%) and 41–50 (27.1%). The age groups 11–20, 51–60, and 61–70 showed lower prevalence rates (8.33%, 5.21%, 4.17%). This distribution sheds light on the age-specific burden of HCV in our study, providing valuable insights for targeted interventions and healthcare strategies.

### Sero prevalence of HCV antibodies among the blood samples

During the current study, a total of 96 blood samples were obtained from diagnostic laboratories. 80.20% of the tested sera were found to be positive for anti-HCV antibodies employing Anti-HCV ELISA method (Fig. 3). The presence of antibodies (anti-HCV Ab +) indicates an exposure to HCV or active viremia. However, some time it does not indicate current or active HCV infection. The “serologic window” between HCV infection and the detection of specific antibodies varies from patient to patient. Presence of anti HCV with the HCV RNA represents confirmed HCV infections. Therefore, over 50 samples were found positive through amplification by PCR(Supplementary S1).

For the further identification about the presence and correlation between the viremia (amount of virus present in blood) and viral nucleic acid, PCR amplification was used.

### Amplification of HCV cDNA by reverse transcription

**Figure 2:**
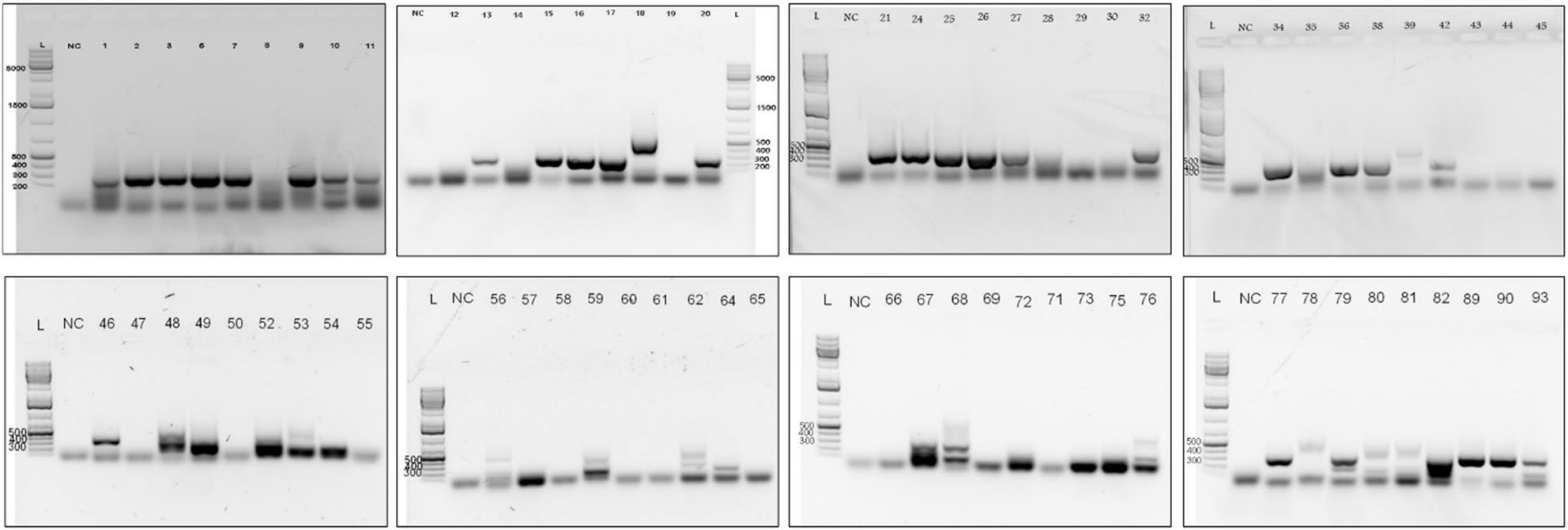
RT-PCR; PCR products of 322 bp on 2% agarose gel electrophoresis before sequencing. Products of PCR standardized in the present study using serum samples infected with HCV. NC Represents the negative control while rest of the other numerical showed the serum samples.

The sizes of PCR product estimated in electrophoresis gel were about 322 bp as predicted (Fig. 2). The present indication confirms the viremia (very strong, strong to low) in patients with reactive anti-HCV results. The 37 samples were further subjected to Sanger Sequencing for genotype determination based on good quality band.

### HCV genotyping of positive sera by Sanger’s sequencing technique

Among all confirmed HCV samples by ELISA and RT-PCR, 40 strongly positive samples were subjected to the Sanger sequencing method of a 5’UTR region, followed by phylogenetic analysis. Identification and confirmation of the HCV subtype, HCV sequences were blasted at NCBI and found more than 98% similarity with 3a 5′ UTR regions and polyproteins of 3a HCV (Table. S1). The datasets generated and/or analyzed during the current study are available in the [National Center for Biotechnology Information] repository, [Accession numbers, GenBank MN153062, MN176509-12, MN184643-46, MN267188 -204 and MN312206-19].

**Figure 3:**
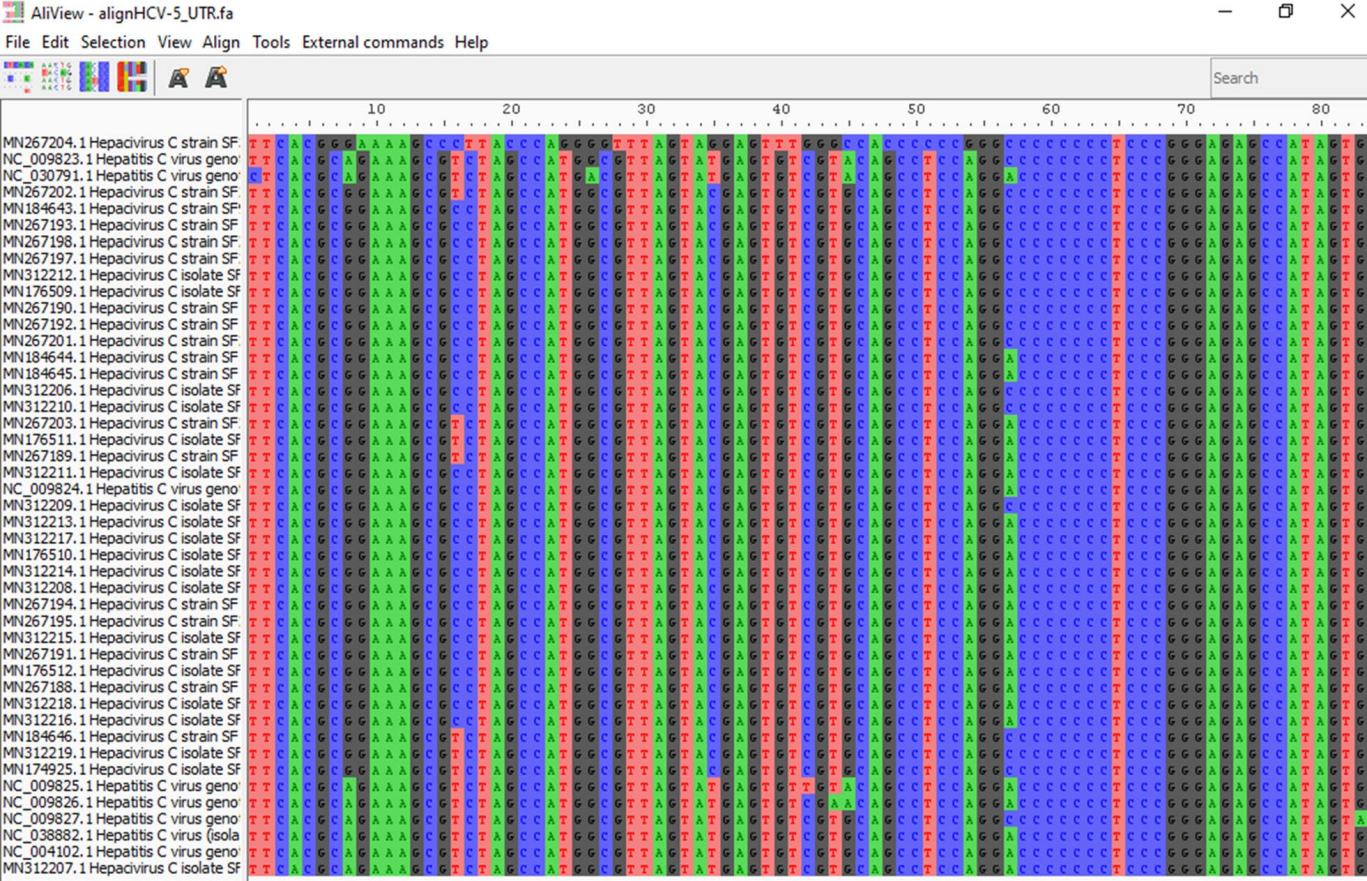
Alignment of the sequence with reference HCV-3a using the Muscle software for multiple sequence alignment program to generate alignments between reference and isolated sequences. Colors indicate the different residues. The aligned files were viewed by Aliview. (MEGA-X file is provided in Supplementary material).

Results indicate that evaluated serum samples carries the genotype 3a with 5´ UTR and polyproteins with more than 98% identity in most of the samples which confirms that the sequence contains high level of conserved regions with the genotype 3a (Fig. 3).

### Phylogenetic analysis of HCV sub genotype

**Figure 4:**
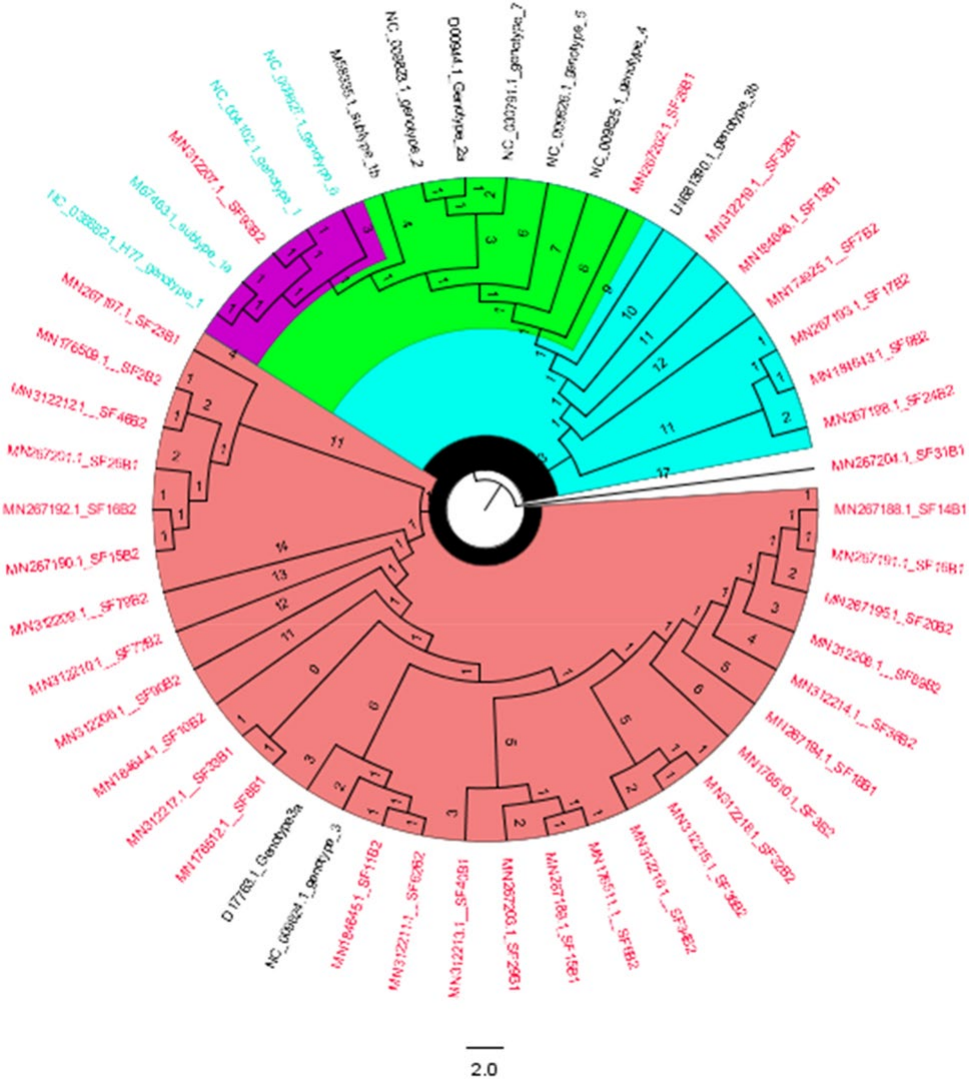
The evolutionary history was inferred via the maximum likelihood method, and the tree with the highest log likelihood (– 723.914137) was displayed. (28/37) sequences were clustered with reference genotype 3a and (7/37) sequences were clustered with genotype 3b and 1/37 sequences was clustered with genotype 1a and one of our sequences was not aligned with any other reference sequences.

Out of all the patients (n = 50), sequencing was successful for samples from 40 patients, and the remaining 3 patients had sequences that showed less similarity during BLAST because of their small size/nucleotide bp and hence were excluded. The sequence alignment of the 5ʹ-UTR isolates from current study was performed with reference genotype (1–7) sequences from the database. The well-conserved areas and few nucleotide substitutions in the 5ʹ-UTR of the HCV genome are shown in Fig. 2. The data also revealed that the length of the 5ʹ-UTR of HCV was up to 350 nucleotides. In the phylogenetic tree, most of the isolates were in a clad and clustered perfectly with the reference sequence. The one isolates from the group did not cluster with the reference sequences (Fig. 4). The phylogenetic tree indicated that all isolates from the current study clustered with with genotype 3a and 3b, and one sequence was clustered with genotype 1a. None of our isolate was clustered with reference genomes of genotype 2, 4, 5, 6, and 7. It indicates that genotype 3a is endemic in this region.

**Figure 5:**
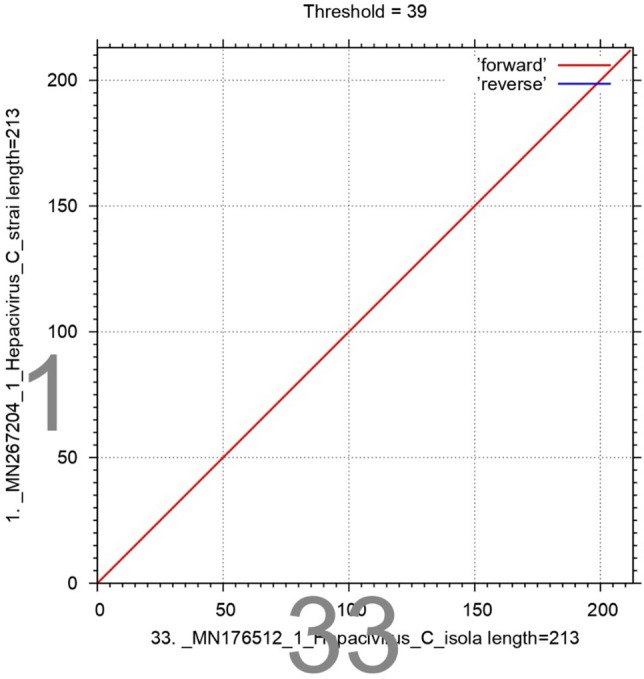
Sequencing of the HCV 5ʹ UTR was performed with a forward primer (red color), and the nucleotide similarity of the 5ʹ UTR sequence was checked by using multiple alignment fast Fourier transform (MAFFTversion7software: https://mafft.cbrc.jp/alignment/server/large.html). All the sequences show significant similarity with sample 1, and sequence no. 2 shows significant similarity with sequences no. 8 and 33.

Sequencing of the HCV 5ʹ UTR was performed with a forward primer (red color), and the nucleotide similarity within the 5' UTR sequences was assessed through the application of the MAFFT version 7 software (https://mafft.cbrc.jp/alignment/server/large.html). Notably, all sequences exhibited marked similarity with sample 1, while sequence 2 displayed significant resemblance to sequences 8 and 33 (Fig. 5).

## Discussion

As far as the prevalence of HCV in developing countries is concerned, Pakistan stands top among them. Generally, in clinical settings for diagnostics, it was consistently reported that screening by immuno-chromatographic test (ICT) method may produce false positive results, and thus it is not recommended for HCV detection^37^. ELISA-based methods for the seroprevalence of HCV are recommended, and now third-generation ELISA kits are available for accurate detection (CADTH, Review). Detection of antibodies to HCV infection is routinely performed by ELISA with high sensitivity and specificity^38^.

The main limitation for the confirmed diagnosis of the virus is due to the lack of standardization in most of the serology protocols.

To confirm the results of serological marker, and to minimize the impact of the instability, a RT-PCR (RT-PCR) method, based on a particular primer design strategy was developed^39^. Molecular virological techniques always play a key role in the accurate diagnosis, and monitoring of treatment for HCV. Nucleic acid amplification (NAT) is the ‘gold standard’ for the detection of active HCV replication^40^. It is exceptionally useful for the diagnosis of acute HCV infection, because of the detection of RNA as early as 1 week after exposure via needle-stick or blood transfusion, and at least 4–6 weeks prior to seroconversion^41^. Overall, the detection of HCV by qualitative RNA test is among the extremely sensitive tests used presently. HCV is a RNA virus, and the reverse transcription PCR is used to detect viral RNA^28,42^. To avoid the false negative results, HCV RNA is detected by RT-PCR based method which is suitable for both screening, and clinical diagnosis. Consequently, it is recommended to use both ELISA and RT-PCR for the diagnosis of HCV infection in clinical settings^38^.

Based on blast similarity and phylogenetic tree, we found genotype 3a (75%), the most prevalent genotype in this study followed by genotype 3b (18.9%) and 1a (2.7%). Similar results were found in other studies as well. Genotype 3 is endemic in Pakistan and other neighboring countries like India and Bangladesh^43^. While genotype 1a was found 2.7% in this study is highly prevalent in the United States^44^.

Previously used methods were usually focused on specific regions of the HCV genome or sometimes analyzed the entire genome but in separate fragments, all this is laborious, cost-prohibitive, and leads to wrong analysis. Therefore, there is an urgent need for more sensitive and reliable methods with the ability to amplify the whole viral genome of clinical samples^45^. Regarding countrywide information, it is suggested that HCV 3 was the leading genotype in Pakistan. Other major types include genotype 1 and mixed genotype. It is assumed that this genotype requires shorter duration of treatment as compared to genotype 1, which eventually leads to the reduced cost, and side effects. The predominance of HCV genotype 3 in Pakistani population establish the predominance of this genotype in the surrounding countries, including India, Iran, Bangladesh, and China^46^.

Determination of HCV genotypes by direct sequencing of HCV 5' UTR of isolates is an efficient approach because it does not require any further processing steps. Subsequently, it eliminates the delays and cost involved in carrying out further amplification reactions^47^. Additionally, the direct sequencing of PCR products provides more detailed sequence information than other genotyping assays. This additional information could prove to be quite useful in the detection of novel HCV genotypes^48^. It was also observed in some studies that 3a 5′UTR differed from the 1a 5′UTR by 27 nucleotides and 2 single nucleotide deletions. Thus, the 3a 5′UTR represents a highly deviated 5′UTR among HCV genotypes. In addition, the 5′UTR of HCV genotype 3 was relatively conserved among subtype isolates (HCV databases)^49^. The 5ʹ UTR is commonly used for the identification of genotype because of the 90% sequence identity in its 341 nucleotides. The rate of mutations is rare in 5-UTR, and sometimes compensatory mutations are developed to preserve the base-pairing shape and conserve the structural characteristics associated with translation efficiency^26^. This study indicates that genotype 3 is most prevalent, so therapies should be directed against this genotype in clinical settings. To eliminate HCV according to WHO deadline 2030, in this region, there is urgent need of massive screening.

## Conclusion

Diagnostics played an important role in numerous aspects of HCV, from its discovery to prevention, transmission, treatment management, and, eventual global eradication. Molecular assays are sensitive and specific for detecting HCV, and thus have proven to be effective. The information obtained from the present study from Sindh region of Pakistan provides useful information about the particular genotype 3a for clinical decision making. Knowing the predominant genotypes is important to plan prevention and treatment strategies as the treatment of different genotypes and prognosis differs considerably. Genotype determination is very important, because rapid treatment response was reported for the infections that are associated with 3a. Therefore, it may be used as a marker for a treatment therapy and eventually decreasing the economic burden on the country.

### Supplementary Information

Supplementary Information 1.

Supplementary Information 2.

## Data Availability

The datasets generated and/or analyzed during the current study are available in the [National Center for Biotechnology Information] repository, [GenBank MN153062, MN176509-12, MN184643-46, MN267188 -204 and MN312206-19].

## Accession code

The article contains new sequence data submitted in NCBI with accession numbers (GenBank MN153062, MN176509 -12, MN184643-46, MN267188 -204 and MN312206 -19).
