# Supplementary material for: Determination of hepatitis C virus subtype prevalent in Sindh, Pakistan: a phylogenetic analysis
Source: Sci Rep. 2024 May 15;14:11159. doi: 10.1038/s41598-024-59342-7 (PMC11096182; doi:10.1038/s41598-024-59342-7)
Supplement: Supplementary file 1 — Supplementary Information 1. [file 41598_2024_59342_MOESM1_ESM.pdf]

HCV ELISA

Negative control Absorbance=**0.025, 0.159**

Blank Absorbance=**0.0025**

Positive control values=**4.000, 4.000**

Calculation of Negative Control= $\frac{0.025+0.159}{2} = 0.092$

Calculation of the Cut-off: (C.O) = 0.092+ 0.12= **0.212**

Interpretation Key:

Negative Results	Positive Results	Borderline
(A/ C.O. < 1): Specimens giving absorbance less than Cut-off values are negative.	(A/ C.O. ≥ 1): Specimens giving absorbance equal to or greater than Cut-off values are negative.	(A/ C.O. = 0.9- 1.1): Specimens with absorbance to Cut-off ratio between 0.9 and 1.1 are considered borderline.

Result Key: Values > 15= +++, > 10= ++ and < 10= +

S. #	Sample #	Results	Interpretation
1.	1	13.18	++
2.	2	18.86	+++
3.	3	18.43	+++
4.	4	18.86	+++
5.	5	18.86	+++
6.	6	15.96	++
7.	7	18.86	+++
8.	9	17.95	+++
9.	10	18.86	+++
10.	11	18.86	+++
11.	13	18.86	+++
12.	14	18.86	+++
13.	15	18.86	+++
14.	16	18.86	+++
15.	17	18.44	+++
16.	20	16.61	+++
17.	21	17.15	+++
18.	24	18.33	+++
19.	25	18.45	+++
20.	26	18.12	+++
21.	27	18.62	+++
22.	28	18.33	+++
23.	29	18.86	+++
24.	30	18.86	+++

25.	32	15.47	+++
26.	34	17.02	+++
27.	35	18.20	+++
28.	36	15.00	++
29.	38	13.60	++
30.	39	18.05	+++
31.	42	17.66	+++
32.	43	18.86	+++
33.	45	16.90	+++
34.	46	17.16	+++
35.	47	15.00	++
36.	48	14.42	++
37.	49	17.99	+++
38.	50	17.96	+++
39.	52	17.86	+++
40.	53	18.86	+++
41.	54	18.86	+++
42.	55	18.86	+++
43.	56	18.41	+++
44.	57	14.15	++
45.	58	18.29	+++
46.	59	10.09	+
47.	60	15.56	+++
48.	61	12.11	++
49.	62	18.63	+++
50.	64	16.72	+++
51.	65	7.98	+
52.	67	18.86	+++
53.	68	16.20	+++
54.	69	13.46	++
55.	71	12.58	++
56.	72	16.74	+++
57.	73	18.86	+++
58.	75	13.87	++
59.	76	16.26	+++
60.	77	18.20	+++
61.	78	14.49	++
62.	79	10.20	+
63.	82	17.35	+++
64.	89	9.51	+
65.	44	17.90	+++
66.	93	17.02	+++
67.	90	16.55	+++
68.	12	17.68	+++
69.	13	18.86	+++

70.	14	16.69	+++
71.	15	14.95	++
72.	16	17.21	+++
73.	17	18.01	+++
74.	18	8.36	+
75.	19	11.67	++
76.	20	15.97	+++
77.	21	16.70	+++
78.	22	18.86	+++
79.	23	17.21	+++
80.	24	13.59	++
81.	26	18.66	+++
82.	28	18.86	+++
83.	29	17.29	+++
84.	30	18.86	+++
85.	31	17.39	+++
86.	32	16.45	+++
87.	33	17.08	+++
88.	40	18.50	+++
89.	35	18.86	+++
