# Supplementary material for: Determination of hepatitis C virus subtype prevalent in Sindh, Pakistan: a phylogenetic analysis
Source: Sci Rep. 2024 May 15;14:11159. doi: 10.1038/s41598-024-59342-7 (PMC11096182; doi:10.1038/s41598-024-59342-7)
Supplement: Supplementary file 2 — Supplementary Information 2. [file 41598_2024_59342_MOESM2_ESM.pdf]

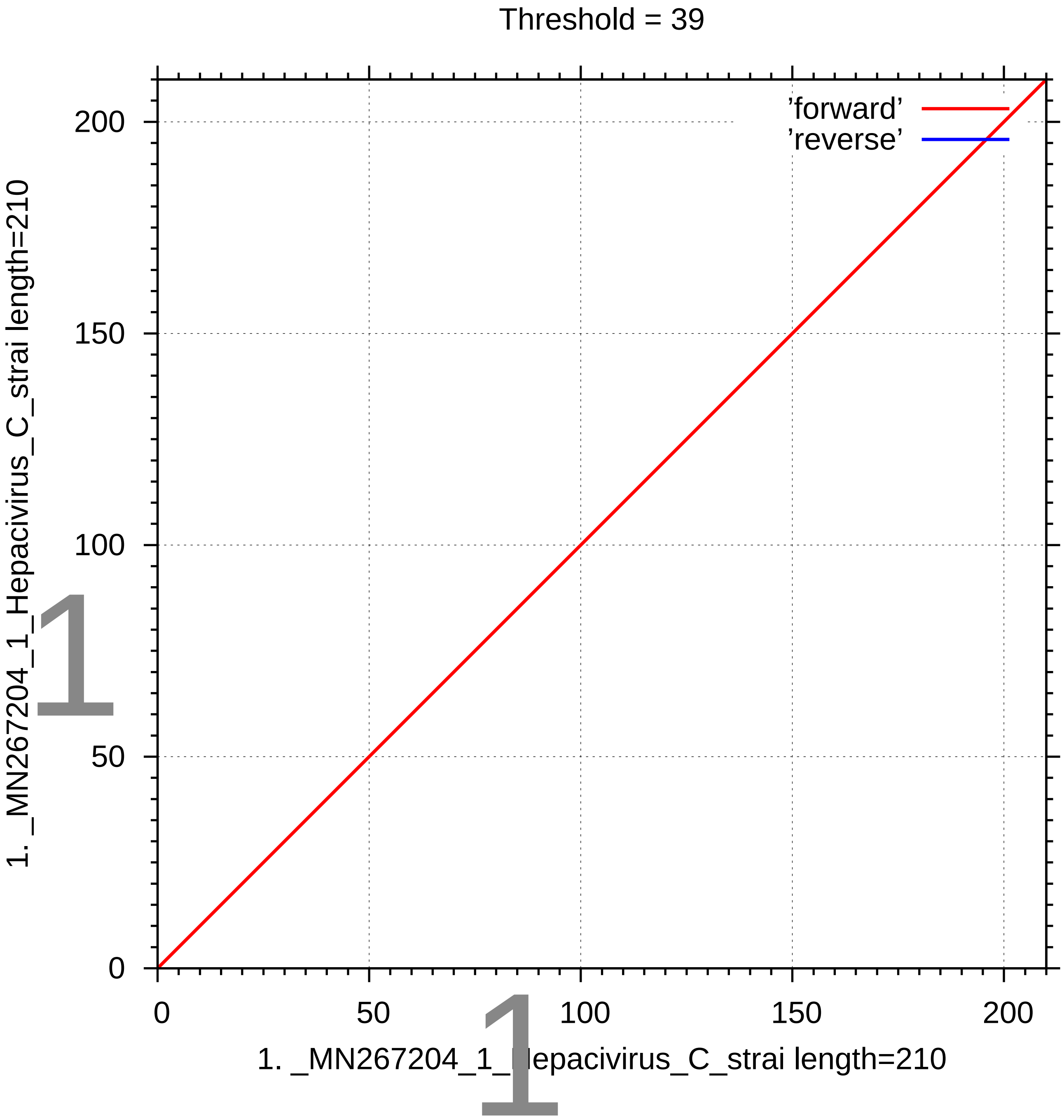

Threshold = 39
'forward'
'reverse'
200
150
100
50
0
0
50
100
150
200
1. _MN267204_1_Hepacivirus_C_strai length=210
1. _MN267204_1_Hepacivirus_C_strai length=210
1
1

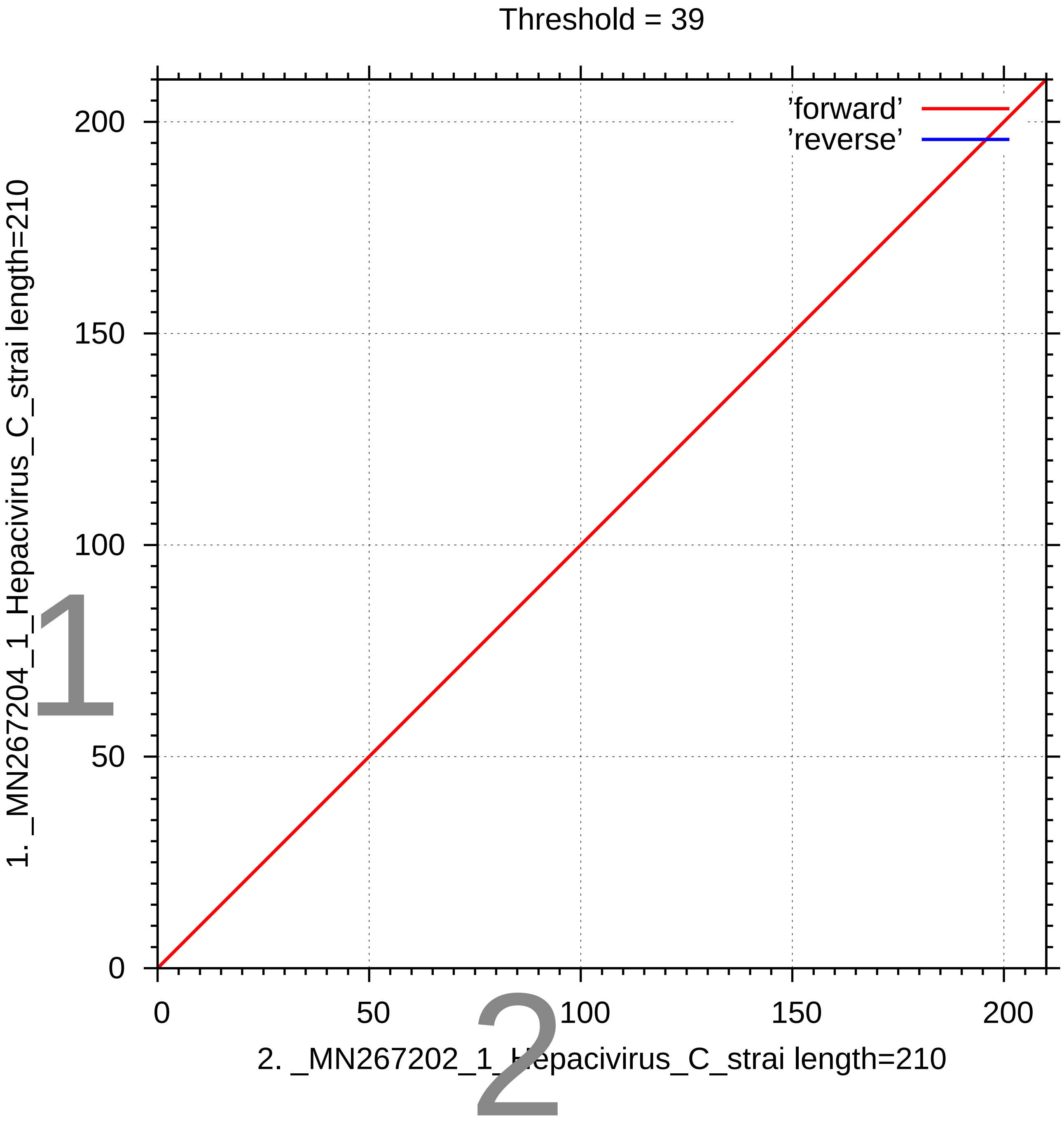
Threshold = 39
'forward'
'reverse'
1. _MN267204_1_Hepacivirus_C_strai length=210
2. _MN267202_1_Hepacivirus_C_strai length=210
0
50
100
150
200
1
2

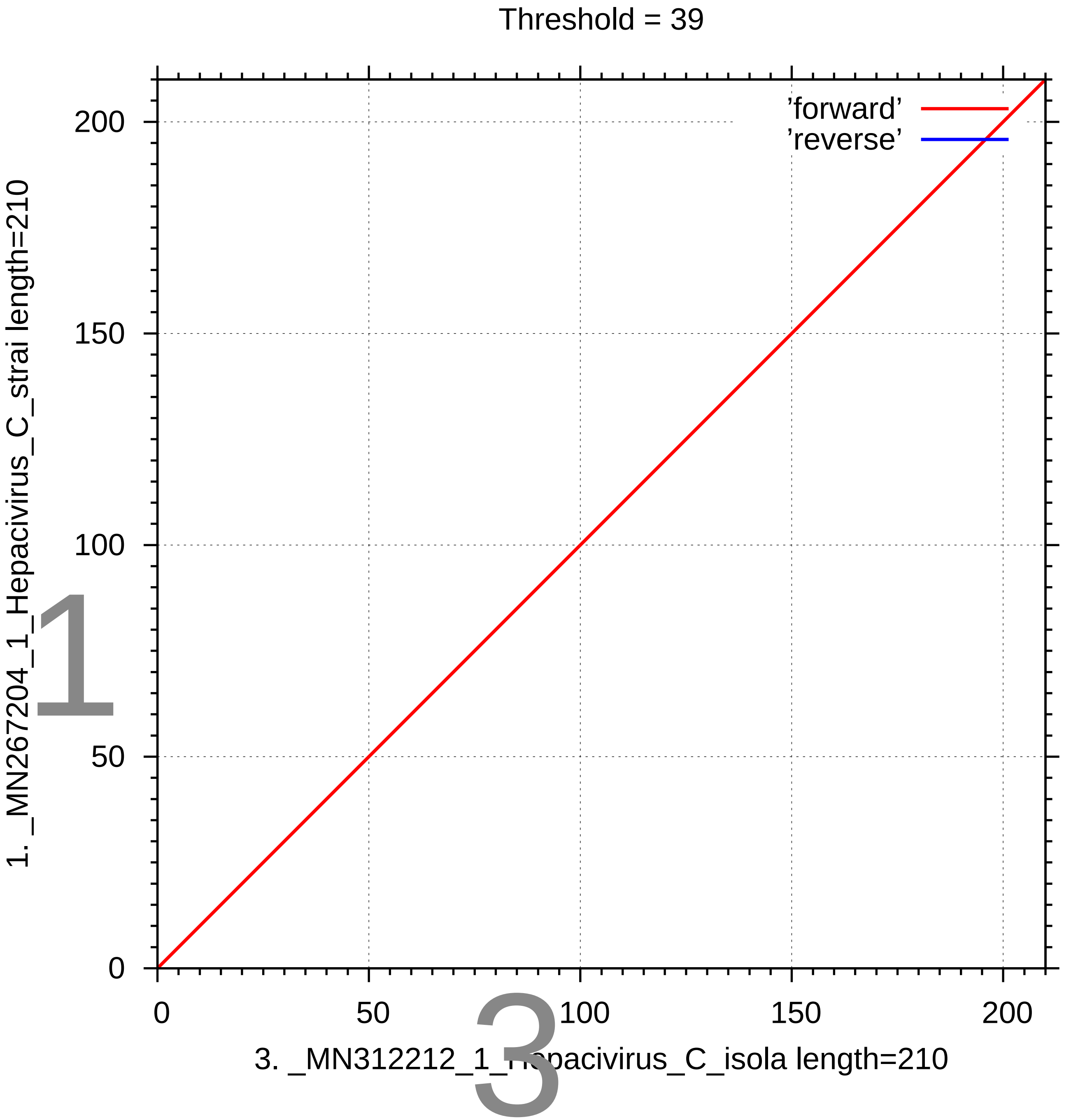

Threshold = 39
'forward'
'reverse'
1. _MN267204_1_Hepacivirus_C_strai length=210
3. _MN312212_1_Hepacivirus_C_isola length=210
0
50
100
150
200
1
3

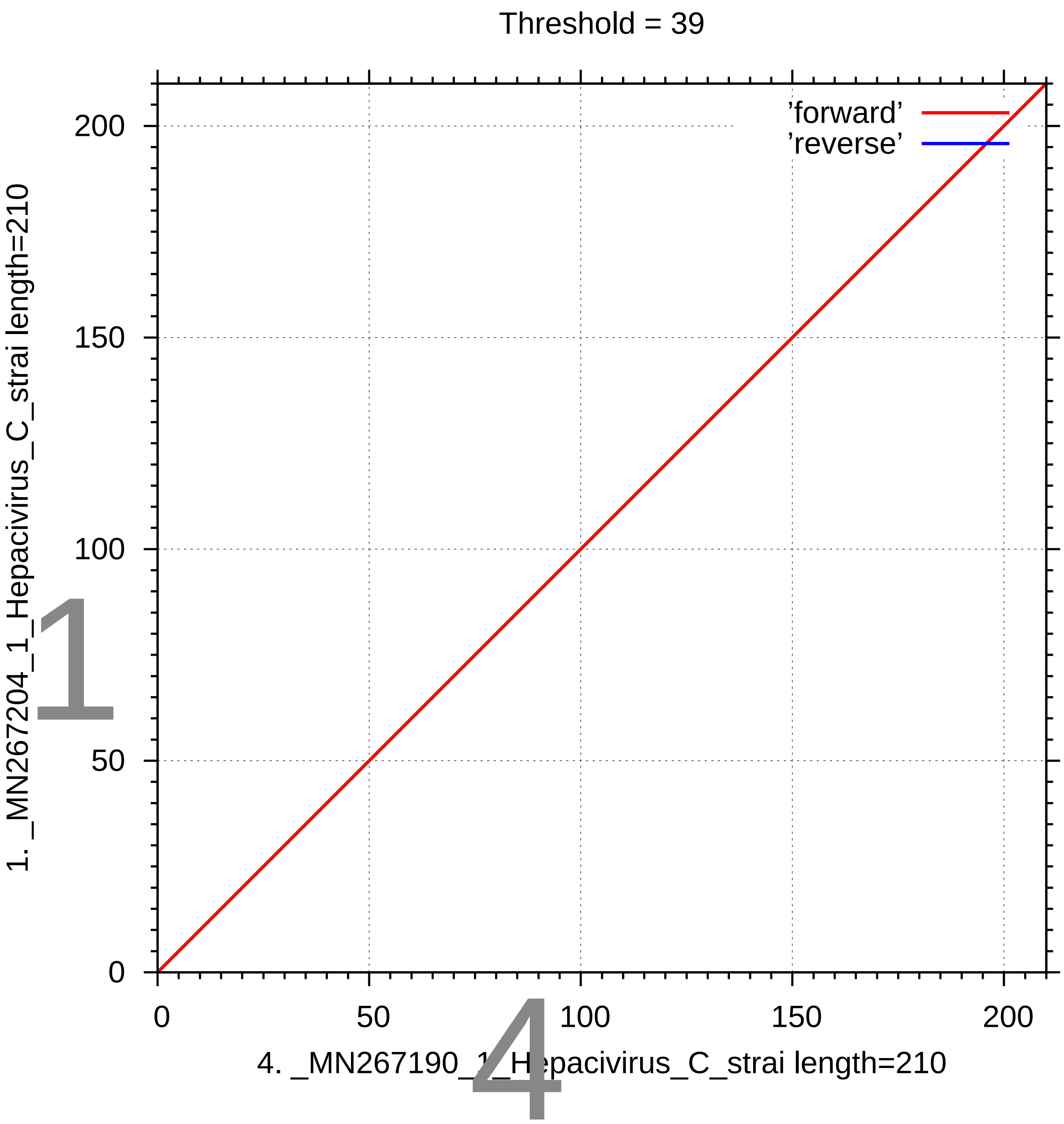
Threshold = 39
'forward'
'reverse'
0
50
100
150
200
1. _MN267204_1_Hepacivirus_C_strai length=210
4. _MN267190_1_Hepacivirus_C_strai length=210
1
4

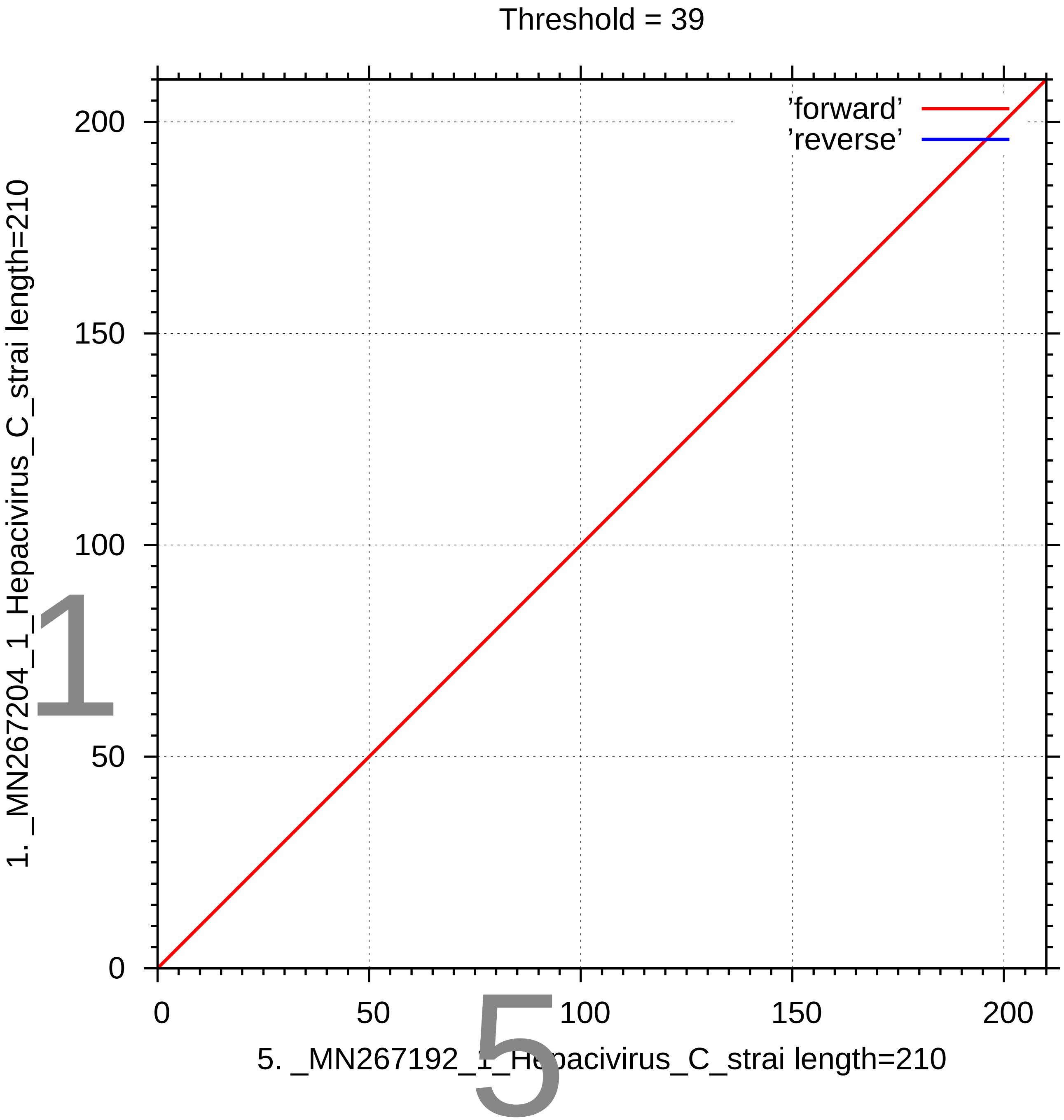
Threshold = 39
'forward'
'reverse'
1. _MN267204_1_Hepacivirus_C_strai length=210
5. _MN267192_1_Hepacivirus_C_strai length=210
0
50
100
150
200
1
5

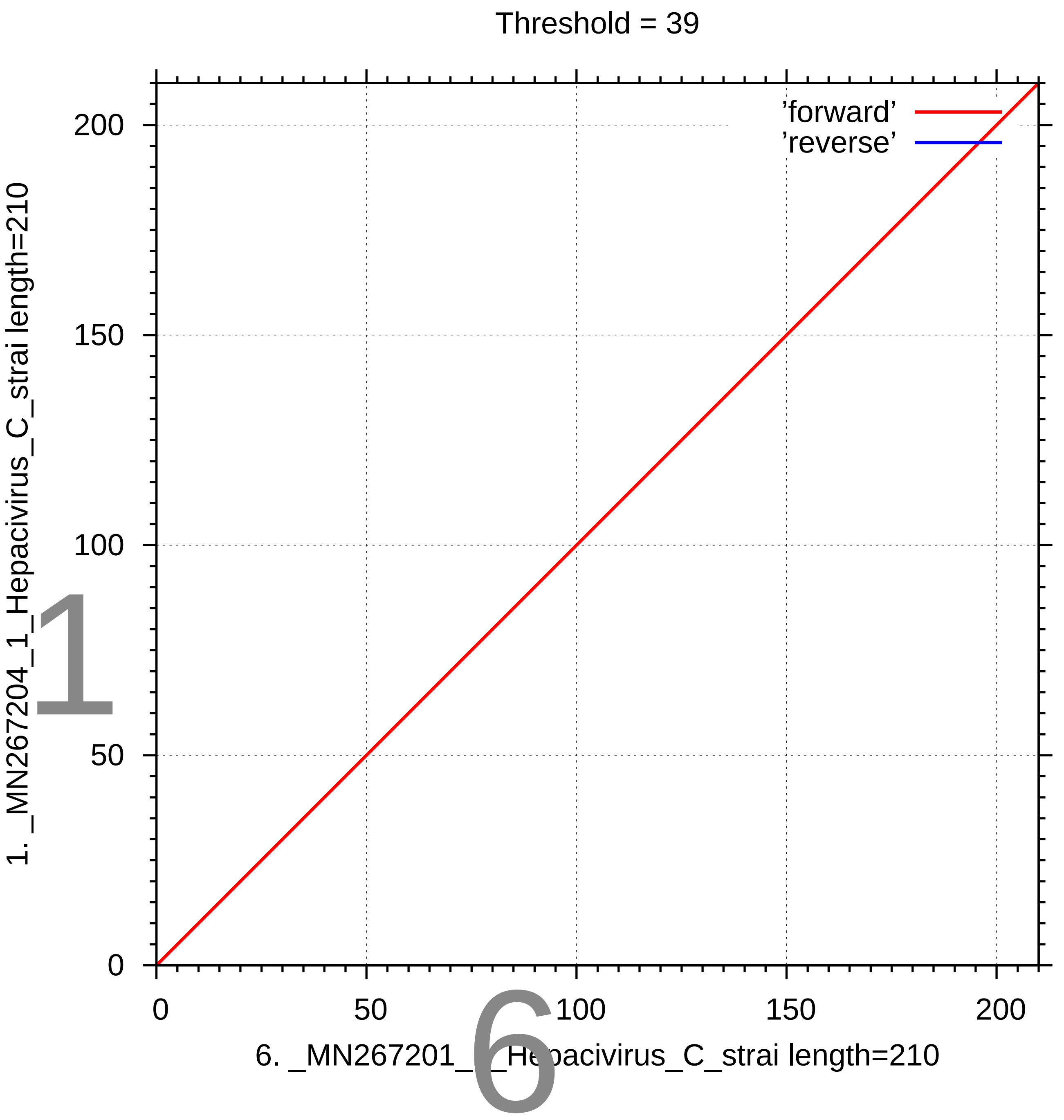
Threshold = 39
'forward'
'reverse'
1. _MN267204_1_Hepacivirus_C_strai length=210
6. _MN267201_ _Hepacivirus_C_strai length=210
0
50
100
150
200
1
6

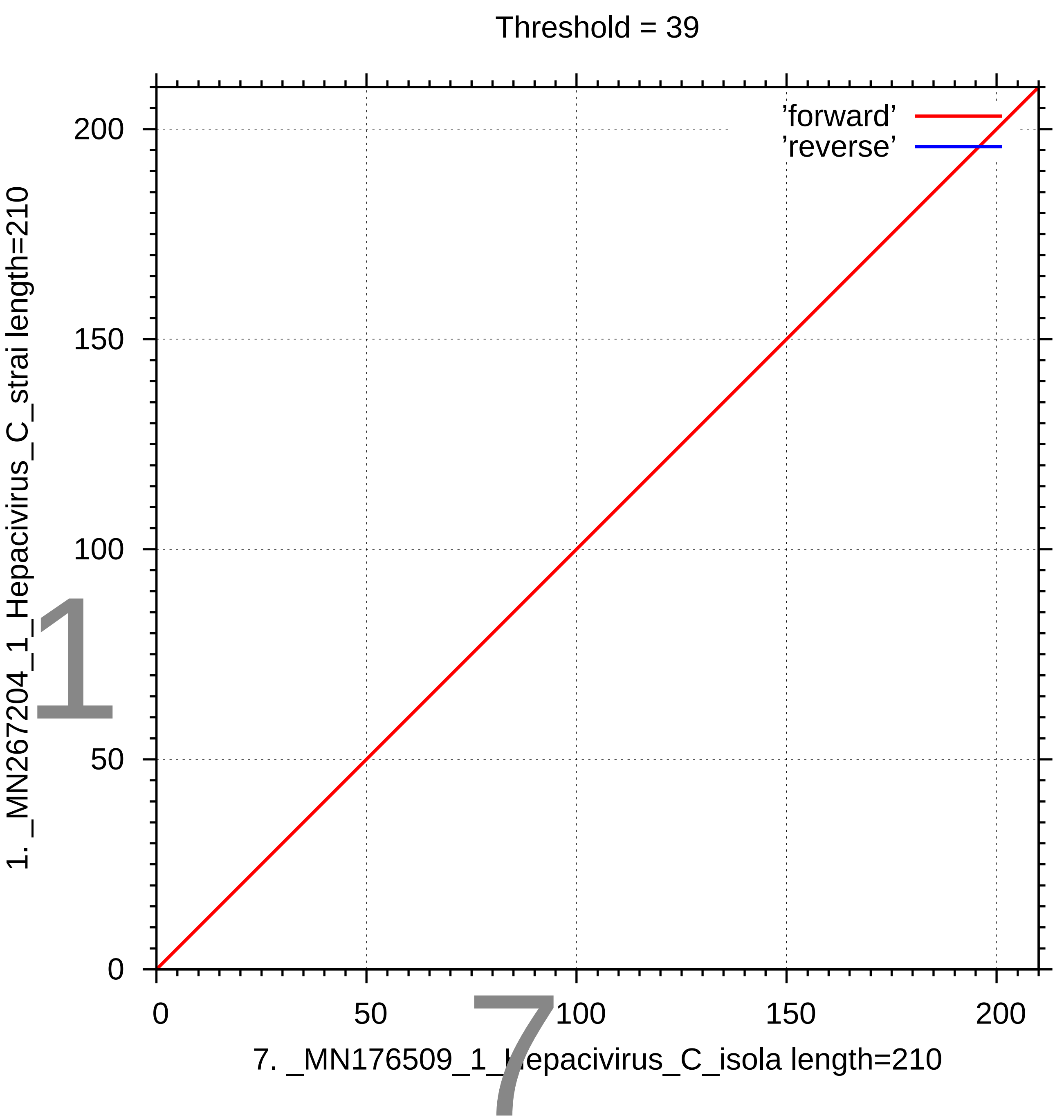
Threshold = 39
'forward'
'reverse'
1. _MN267204_1_Hepacivirus_C_strai length=210
7. _MN176509_1_Hepacivirus_C_isola length=210
0
50
100
150
200
1
7

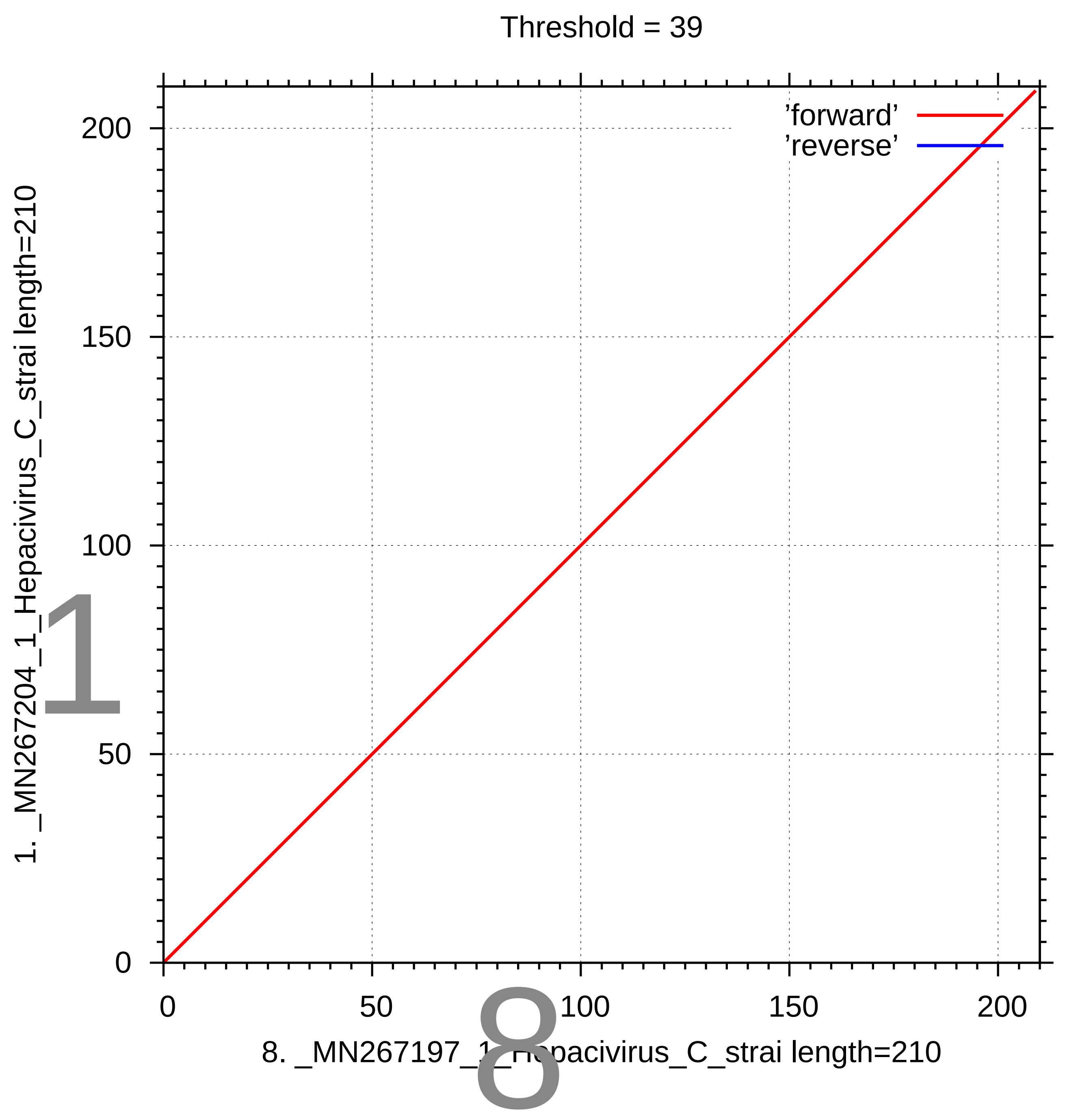
Threshold = 39
'forward'
'reverse'
0
50
100
150
200
0
50
100
150
200
1. _MN267204_1_Hepacivirus_C_strai length=210
8. _MN267197_1_Hepacivirus_C_strai length=210
1
8

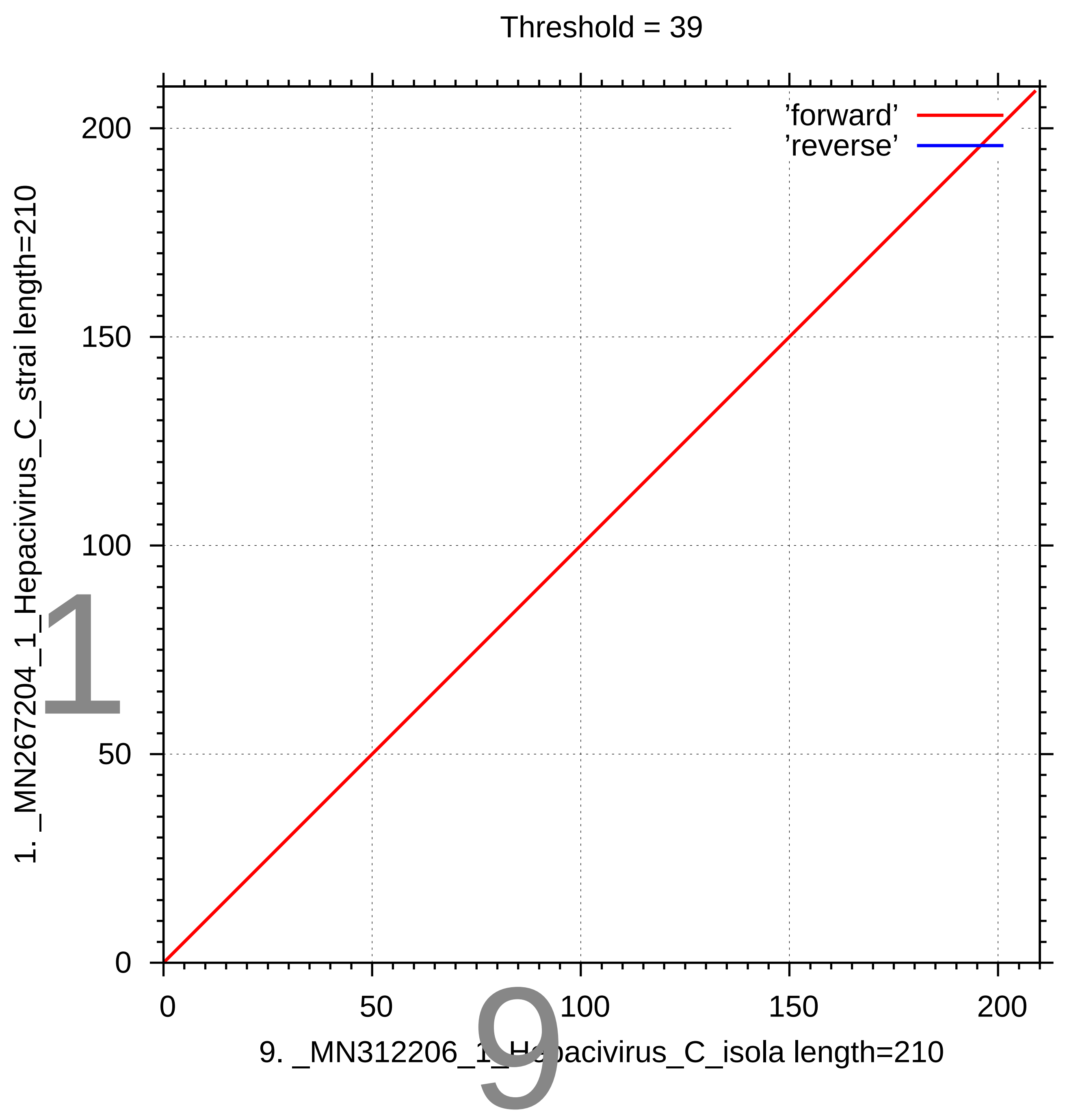
Threshold = 39
'forward'
'reverse'
0
50
100
150
200
0
50
100
150
200
1. _MN267204_1_Hepacivirus_C_strai length=210
9. _MN312206_1_Hepacivirus_C_isola length=210
1
9

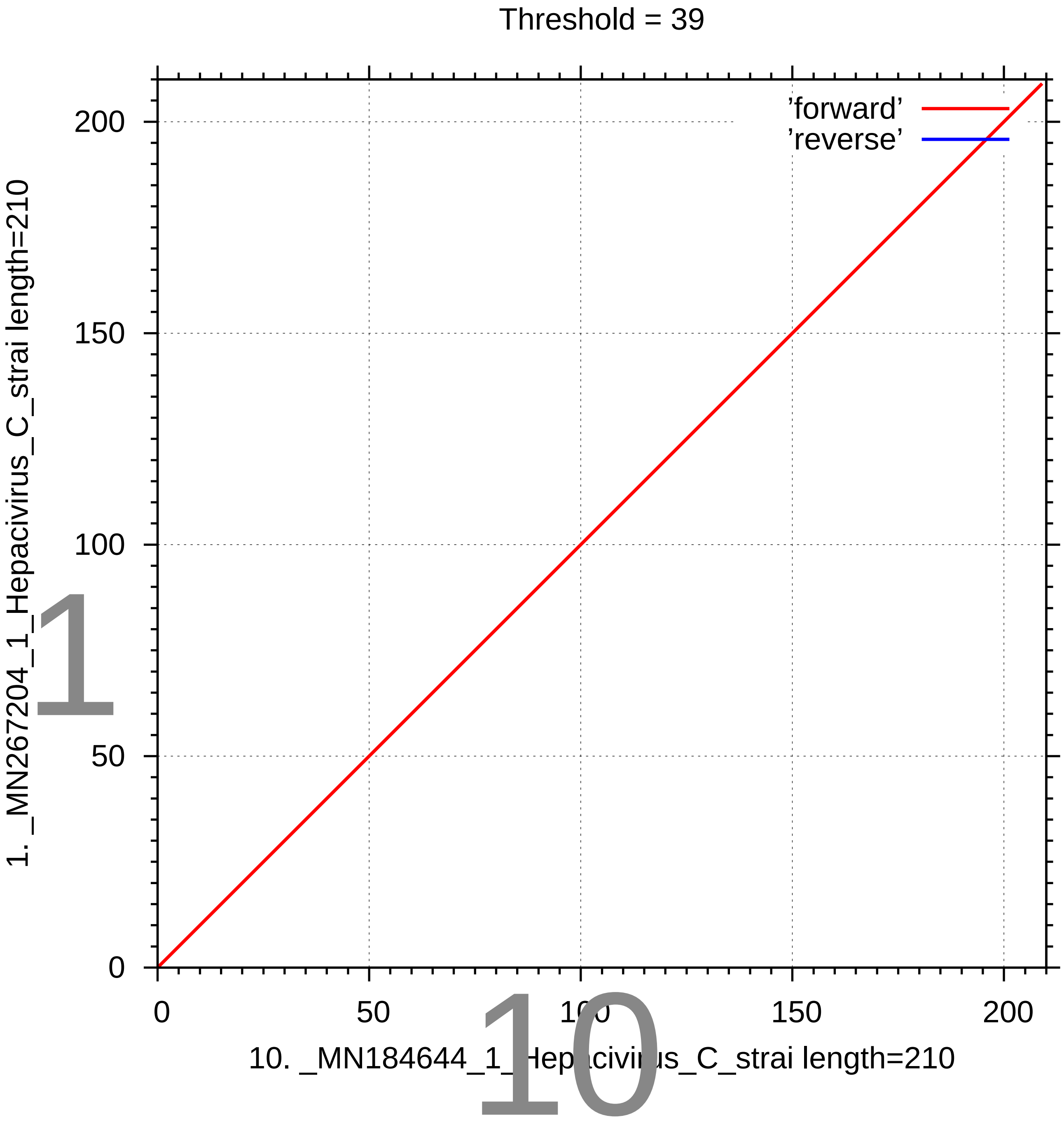
Threshold = 39
'forward'
'reverse'
1. _MN267204_1_Hepacivirus_C_strai length=210
10. _MN184644_1_Hepacivirus_C_strai length=210
0
50
100
150
200
1
10

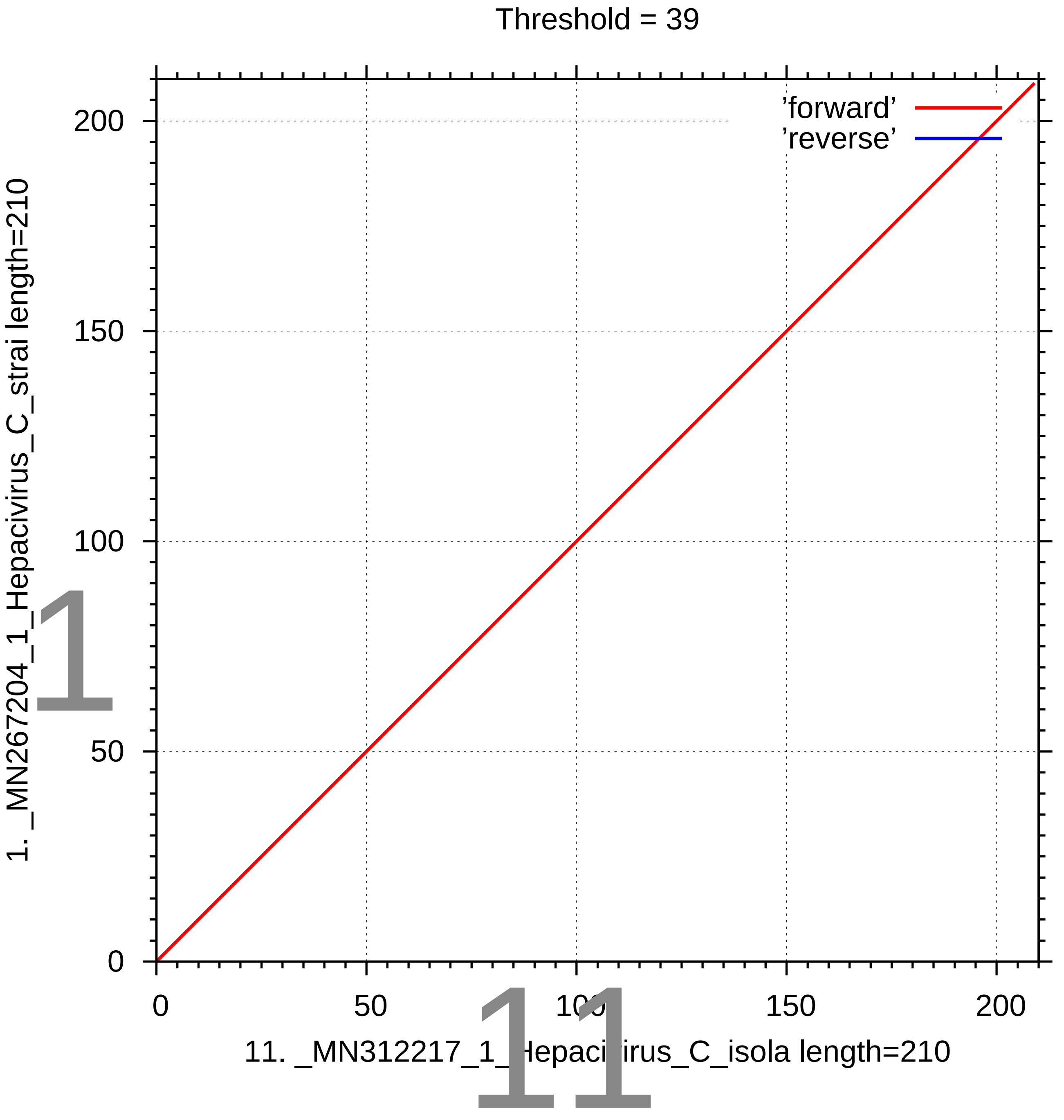
Threshold = 39
'forward'
'reverse'
1. _MN267204_1_Hepacivirus_C_strai length=210
11. _MN312217_1_Hepacivirus_C_isola length=210
0
50
100
150
200
0
50
100
150
200
1
11

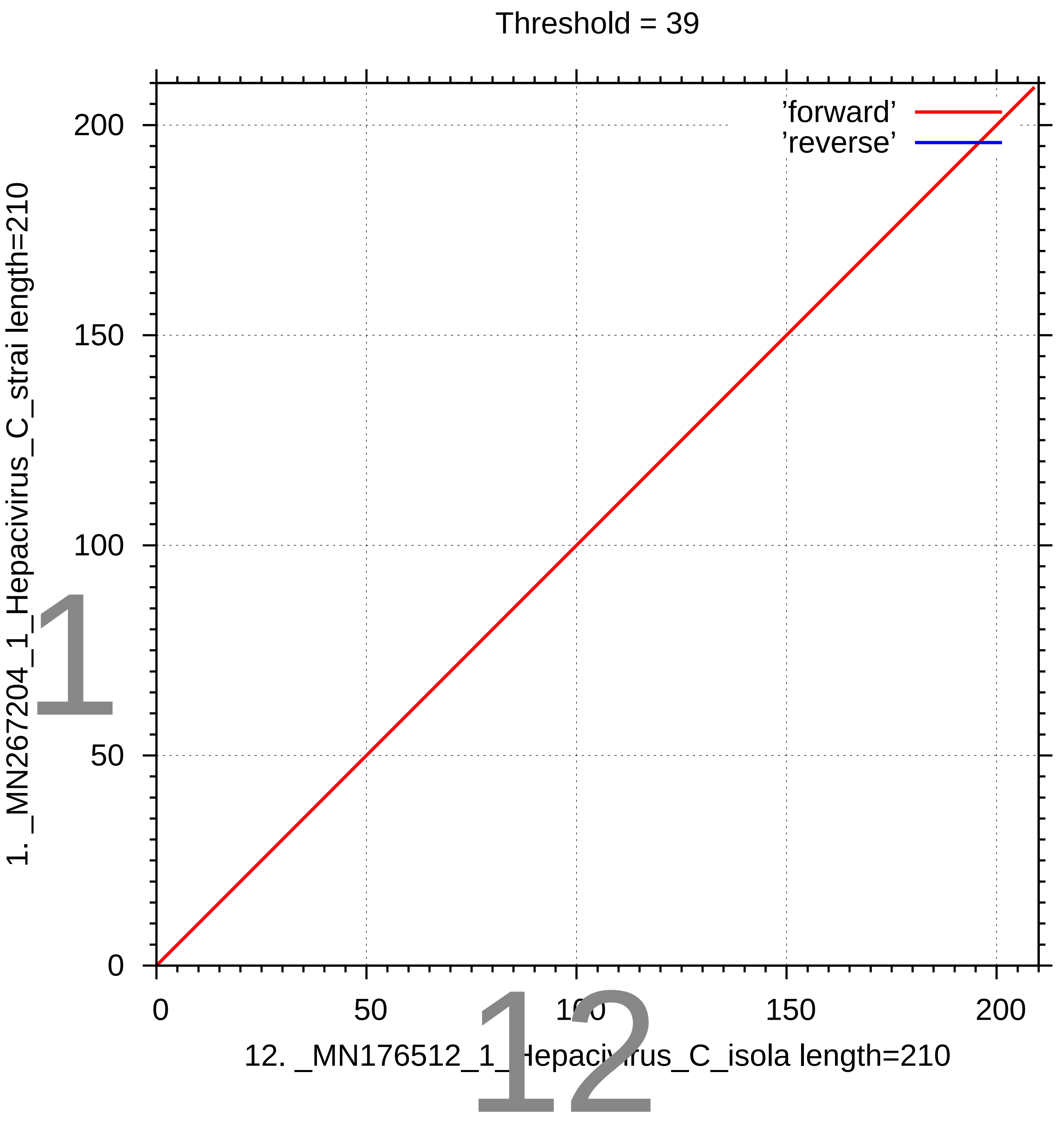

Threshold = 39
'forward'
'reverse'
0
50
100
150
200
0
50
100
150
200
1. _MN267204_1_Hepacivirus_C_strai length=210
12. _MN176512_1_Hepacivirus_C_isola length=210
1
12

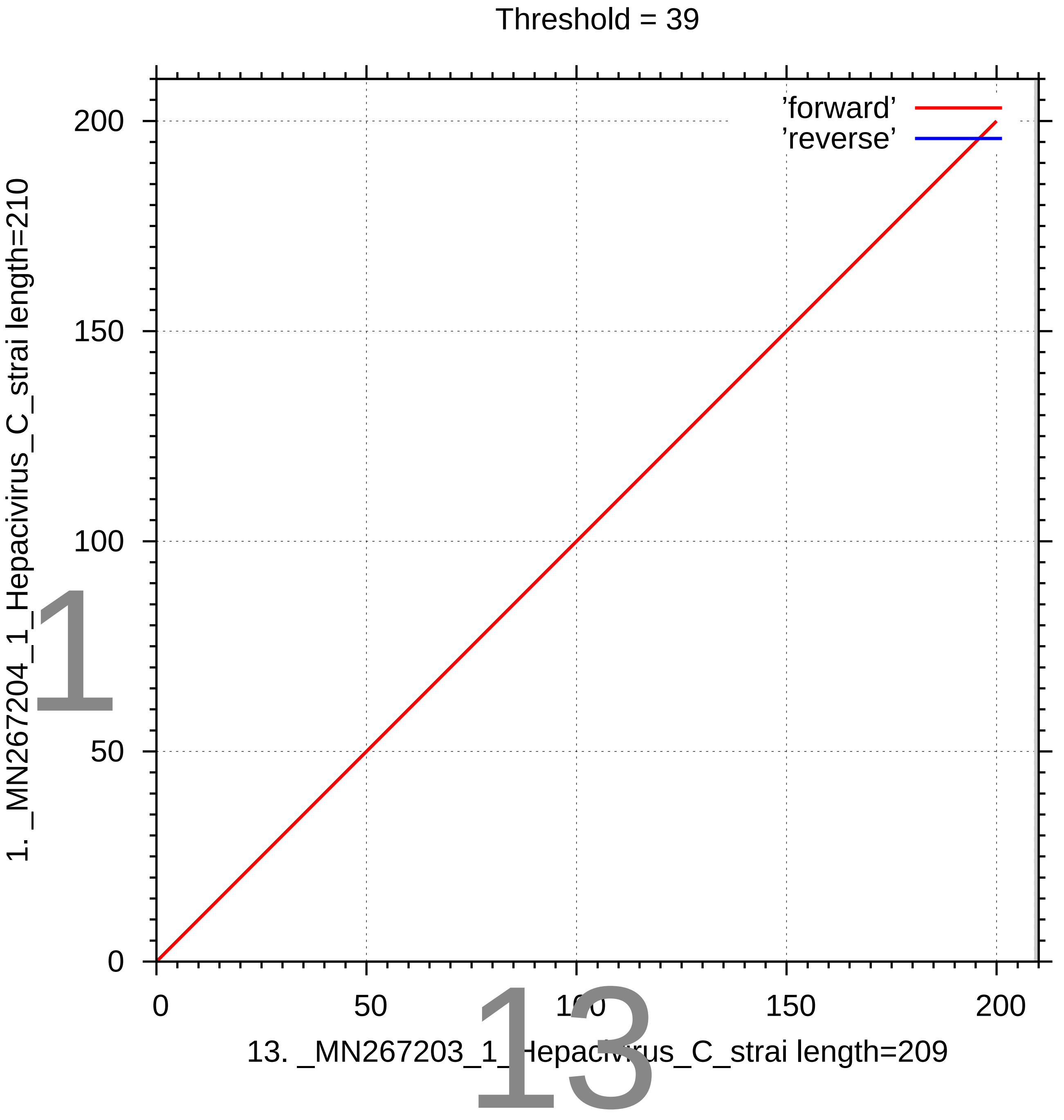
Threshold = 39
'forward'
'reverse'
0
50
100
150
200
0
50
100
150
200
1. _MN267204_1_Hepacivirus_C_strai length=210
13. _MN267203_1_Hepacivirus_C_strai length=209
1
13

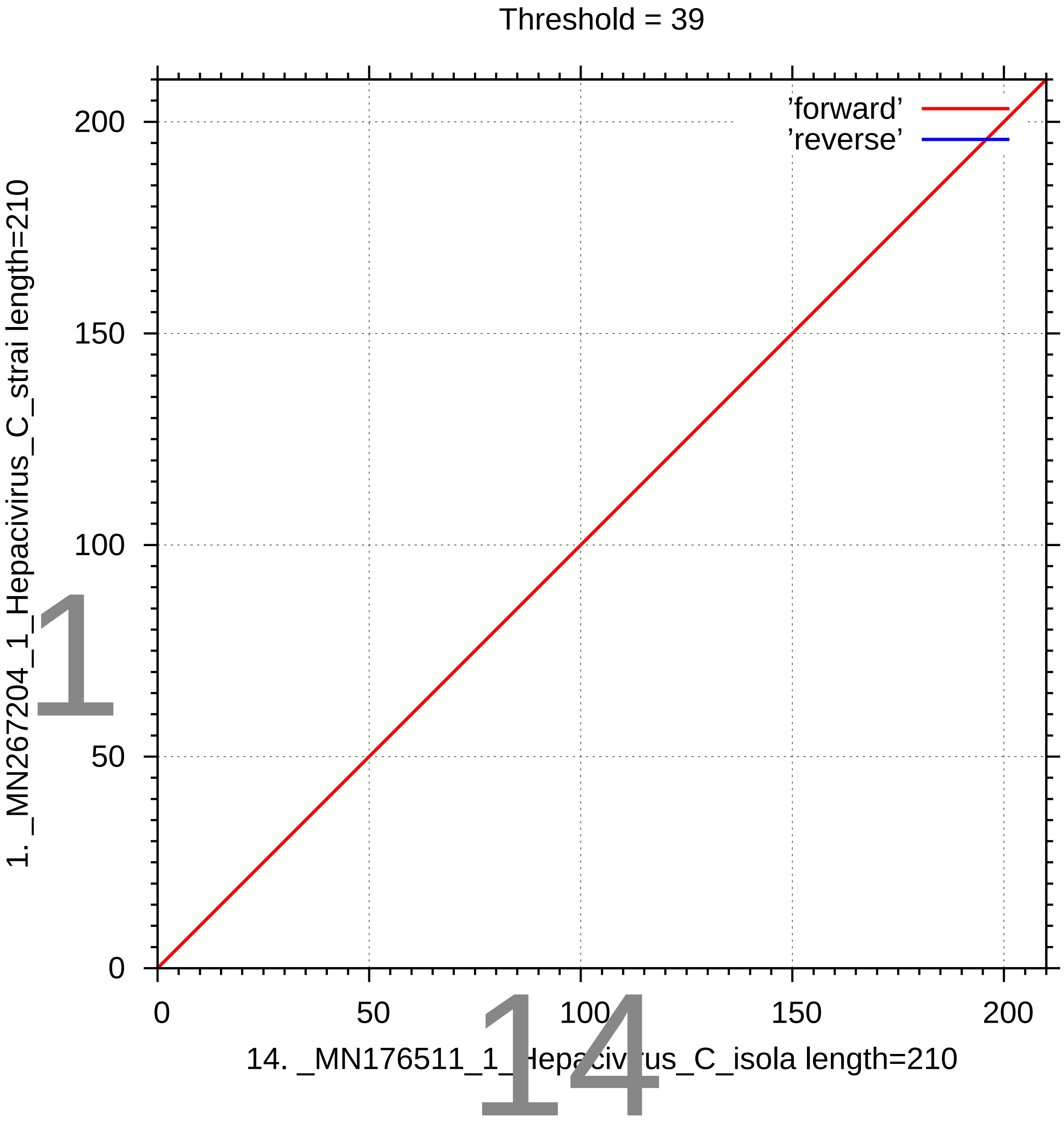
Threshold = 39
'forward'
'reverse'
200
150
100
50
0
0
50
100
150
200
1. _MN267204_1_Hepacivirus_C_strai length=210
14. _MN176511_1_Hepacivirus_C_isola length=210
1
14

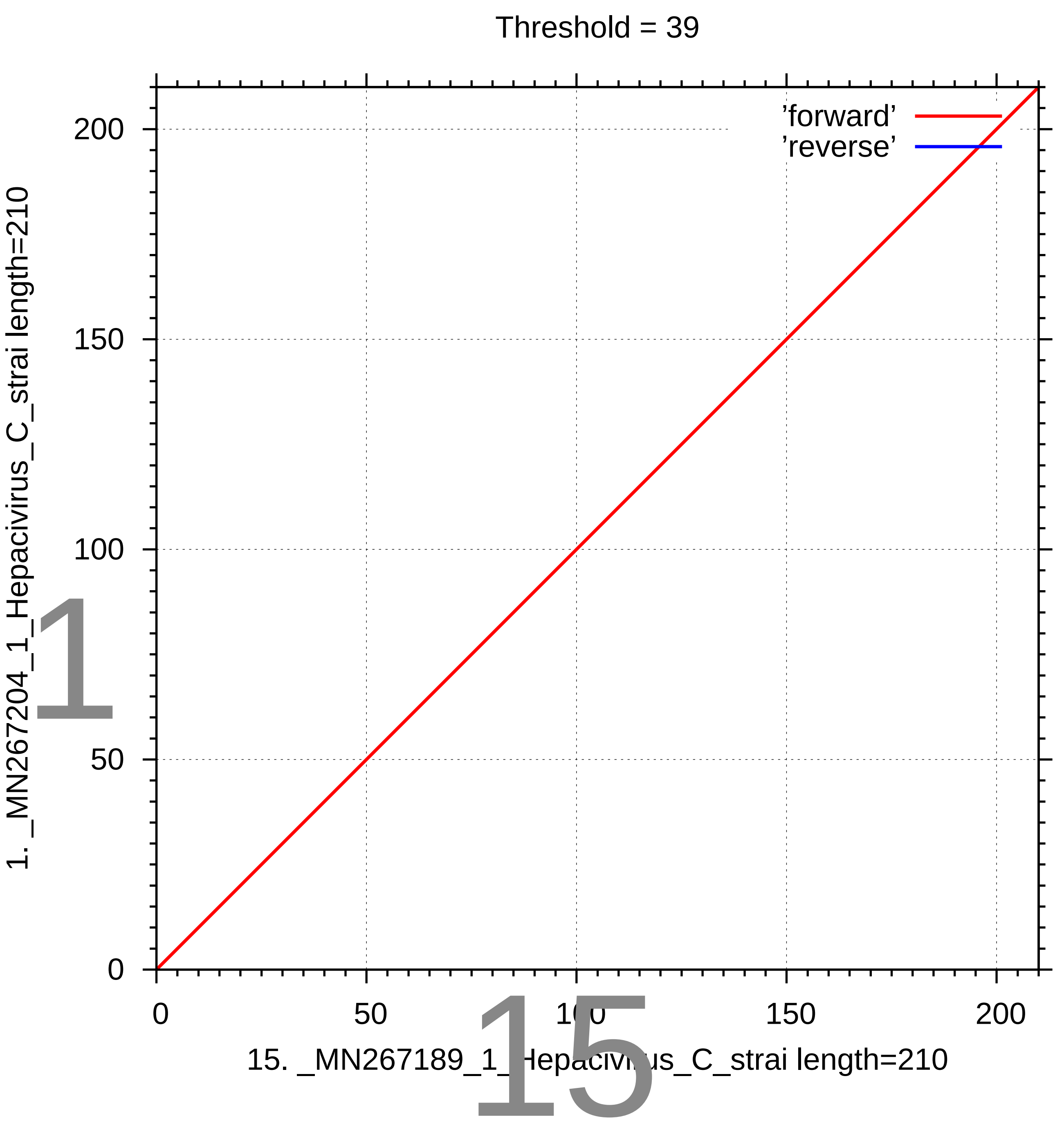
Threshold = 39
'forward'
'reverse'
0
50
100
150
200
0
50
100
150
200
15. _MN267189_1_Hepacivirus_C_strai length=210
1. _MN267204_1_Hepacivirus_C_strai length=210
1
15

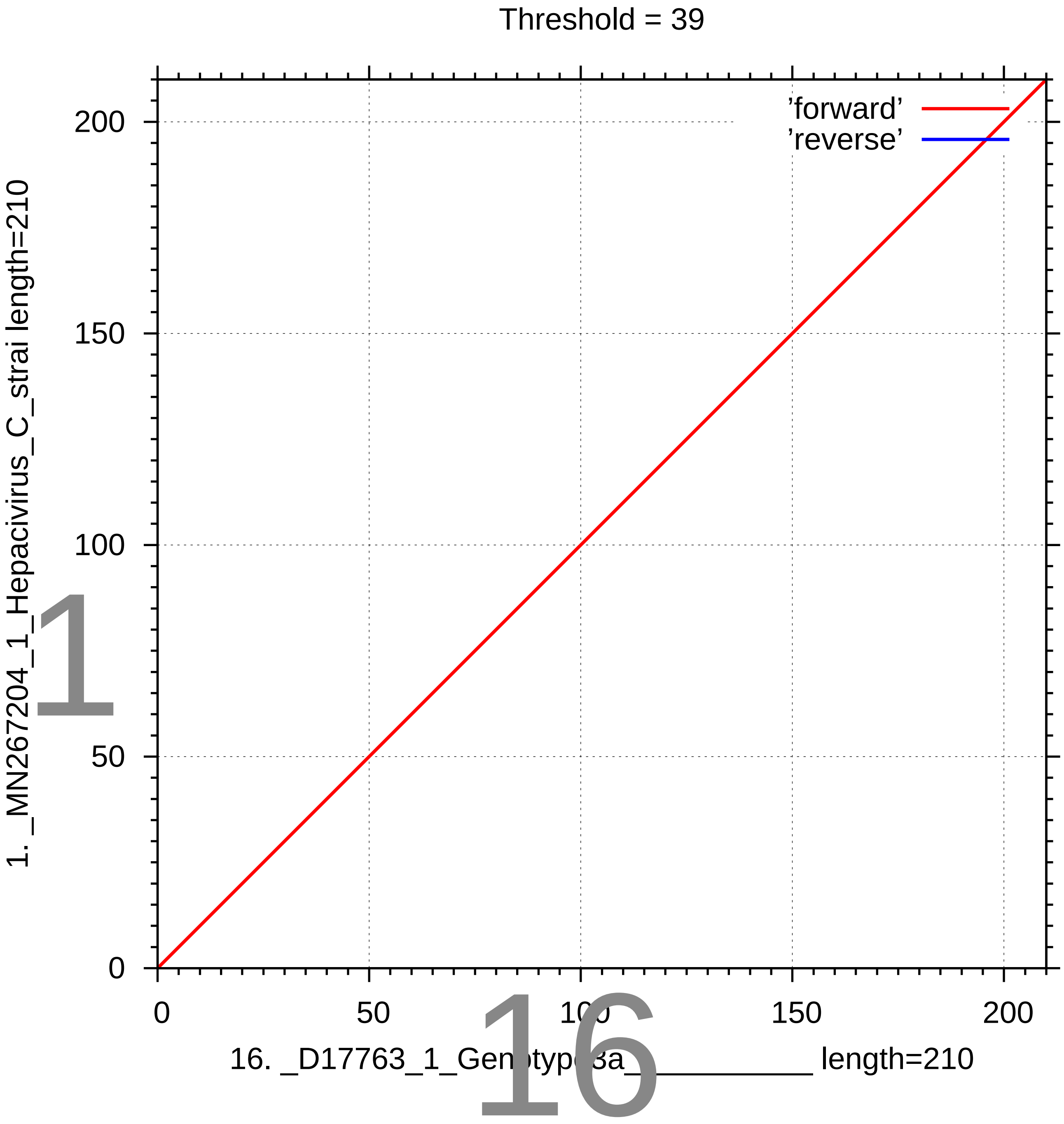
Threshold = 39
'forward'
'reverse'
1. _MN267204_1_Hepacivirus_C_strai length=210
length=210
0
50
100
150
200
1
16

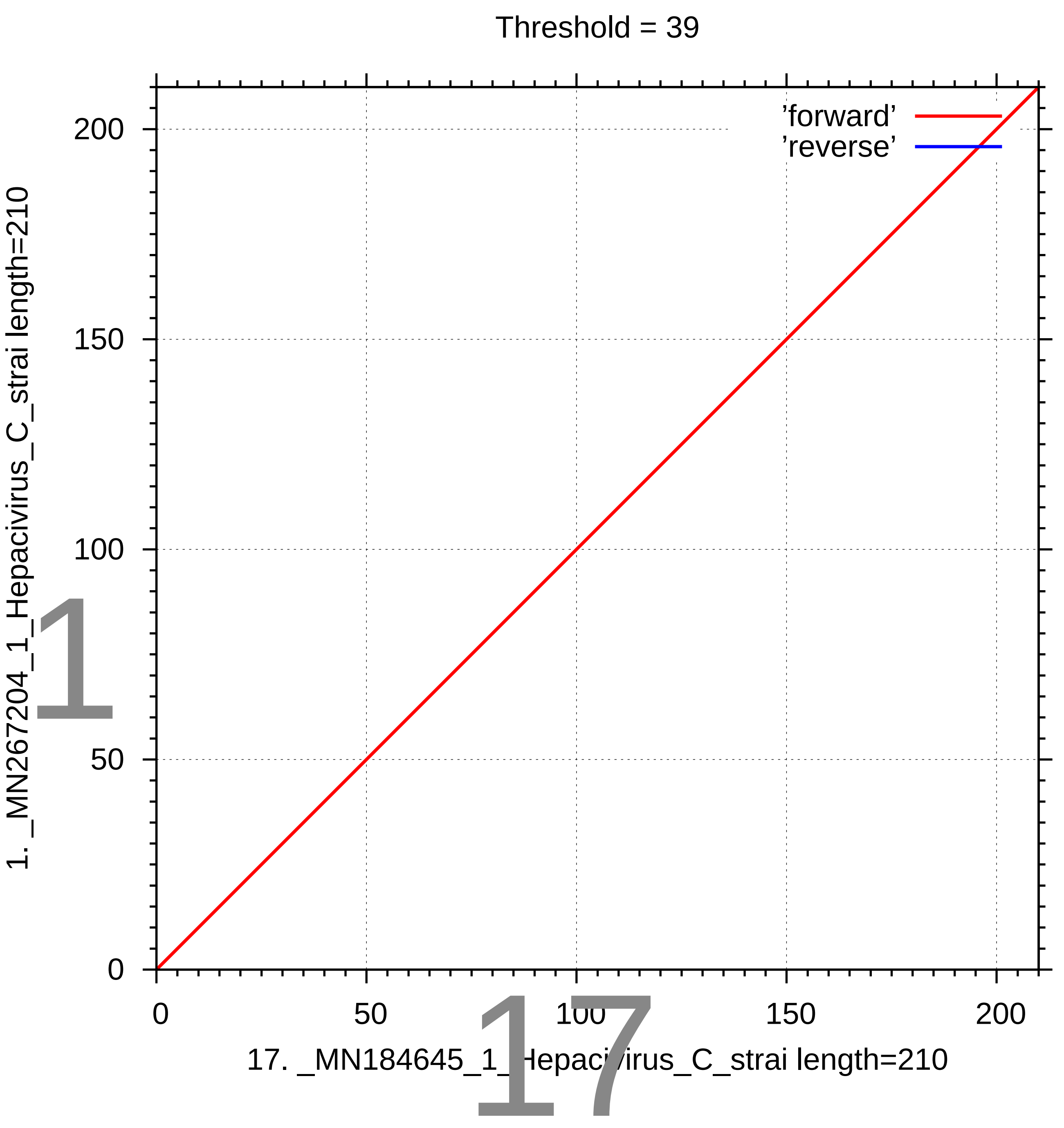
Threshold = 39
'forward'
'reverse'
0
50
100
150
200
0
50
100
150
200
1. _MN267204_1_Hepacivirus_C_strai length=210
17. _MN184645_1_Hepacivirus_C_strai length=210
1
17

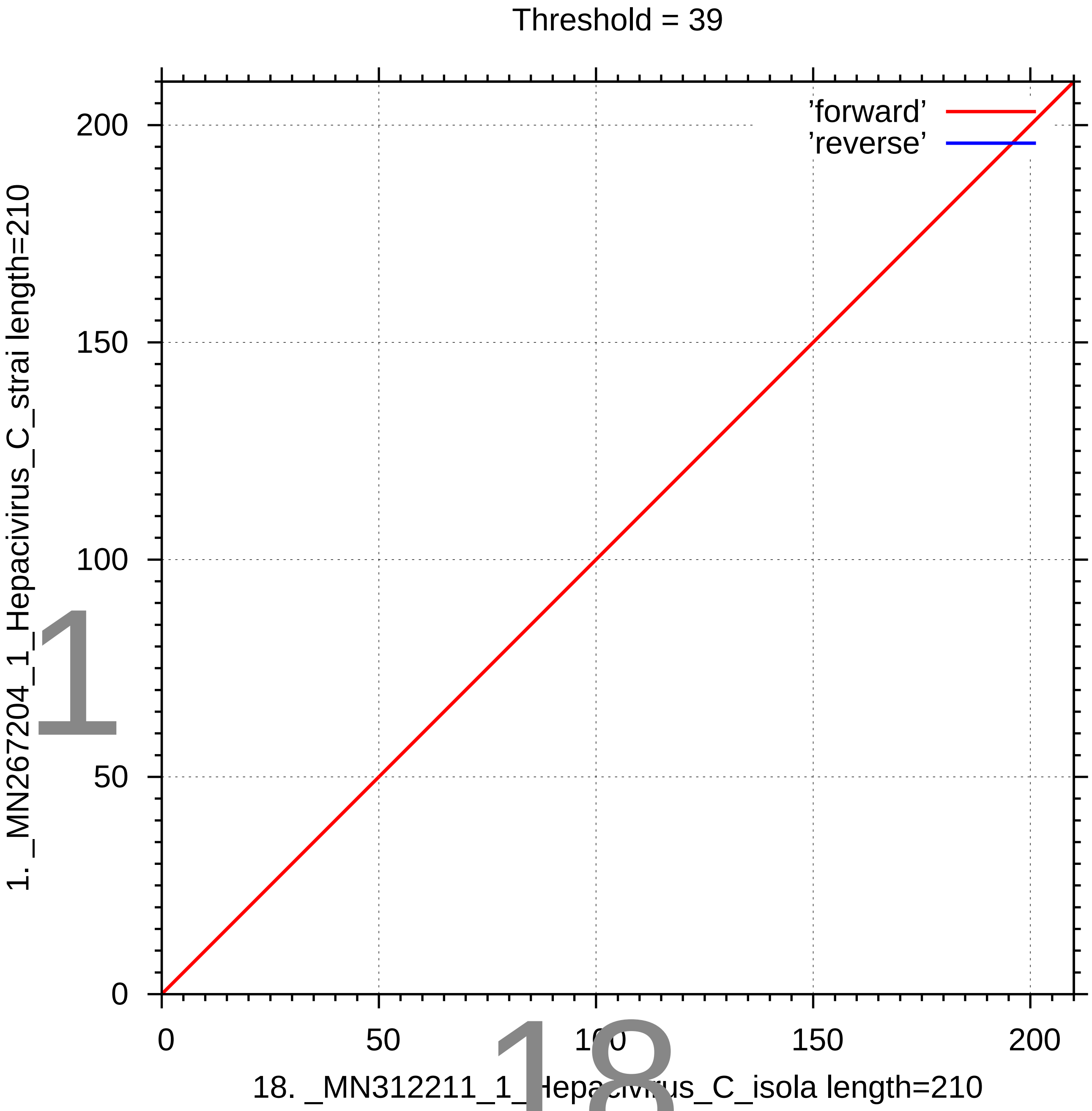

Threshold = 39
'forward'
'reverse'
200
150
100
50
0
0
50
100
150
200
1. _MN267204_1_Hepacivirus_C_strai length=210
18. _MN312211_1_Hepacivirus_C_isola length=210

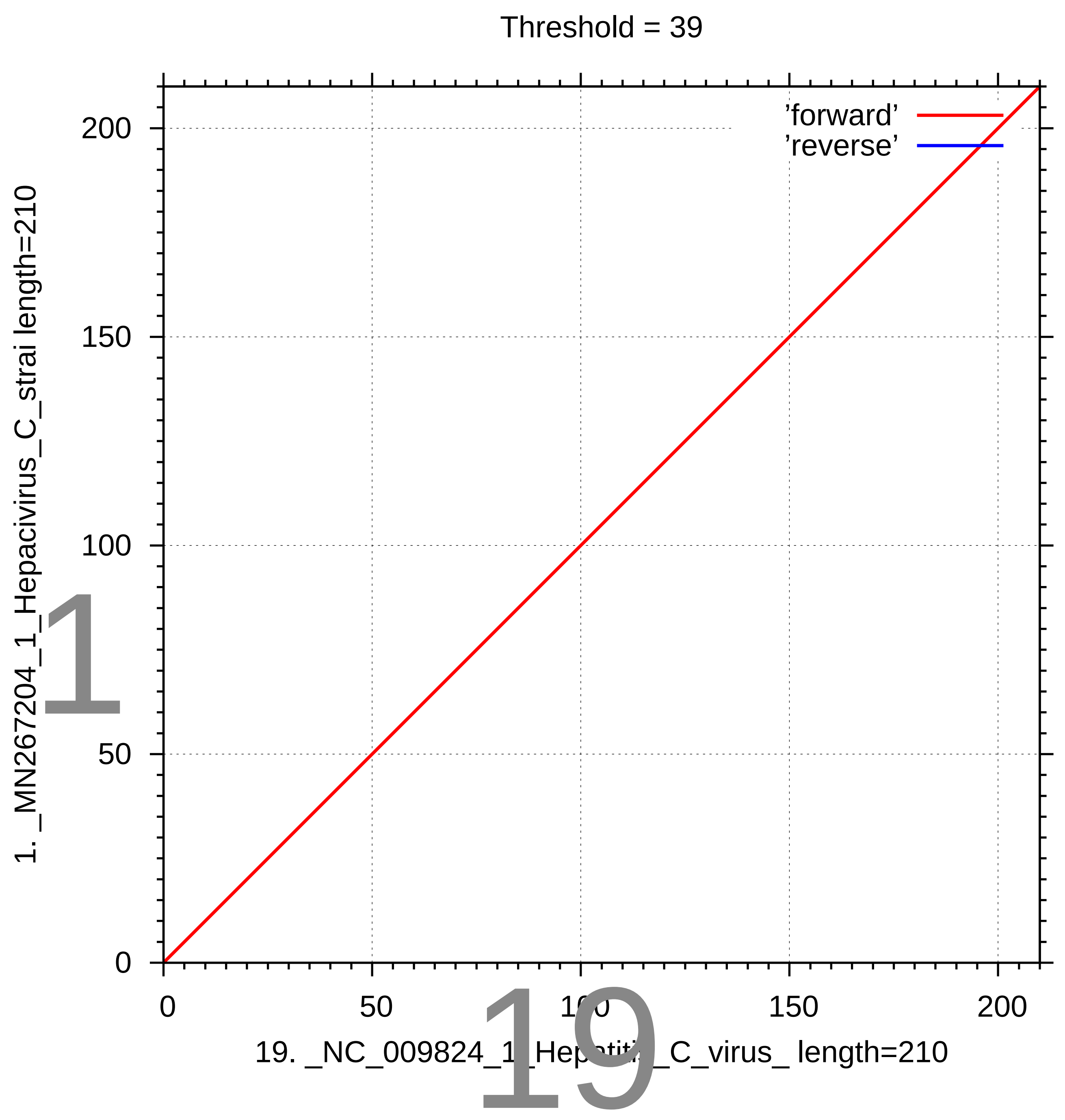

Threshold = 39
'forward'
'reverse'
1. _MN267204_1_Hepacivirus_C_strai length=210
19. _NC_009824_1_Hepatitis_C_virus_ length=210
0
50
100
150
200
1
19

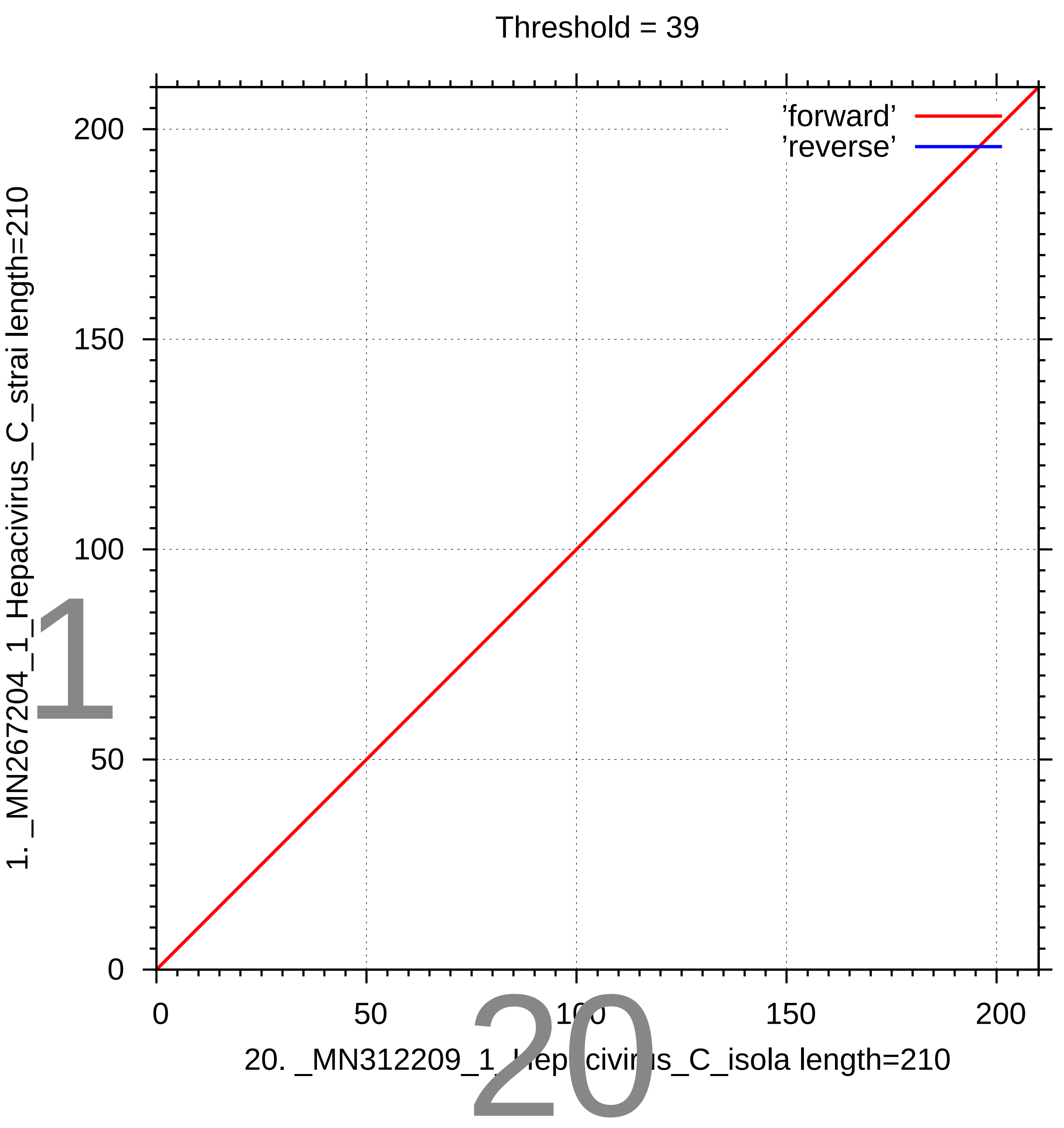
Threshold = 39
'forward'
'reverse'
1. _MN267204_1_Hepacivirus_C_strai length=210
20. _MN312209_1_Hepacivirus_C_isola length=210
0
50
100
150
200
1
20

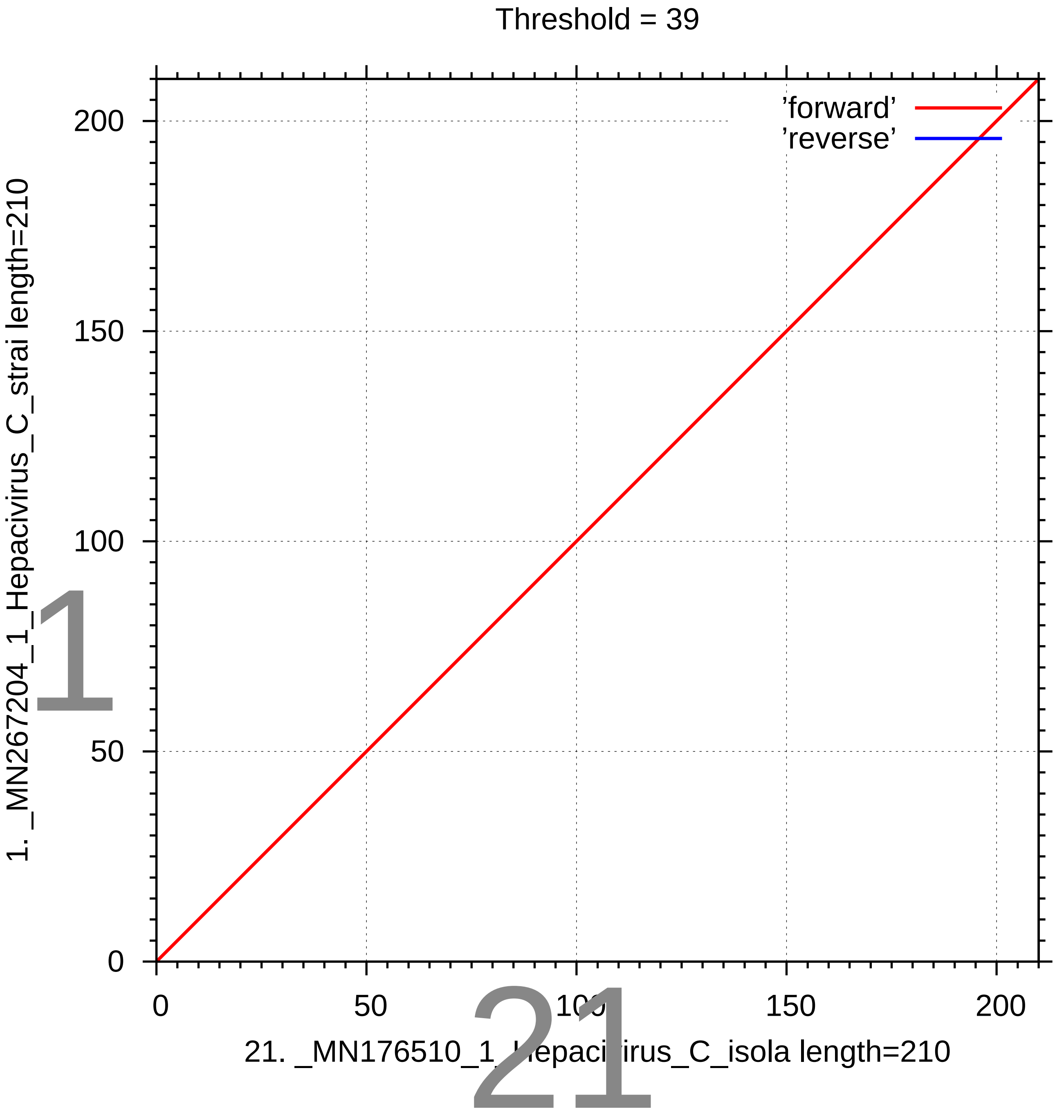
Threshold = 39
'forward'
'reverse'
0
50
100
150
200
1. _MN267204_1_Hepacivirus_C_strai length=210
21. _MN176510_1_Hepacivirus_C_isola length=210
1
21

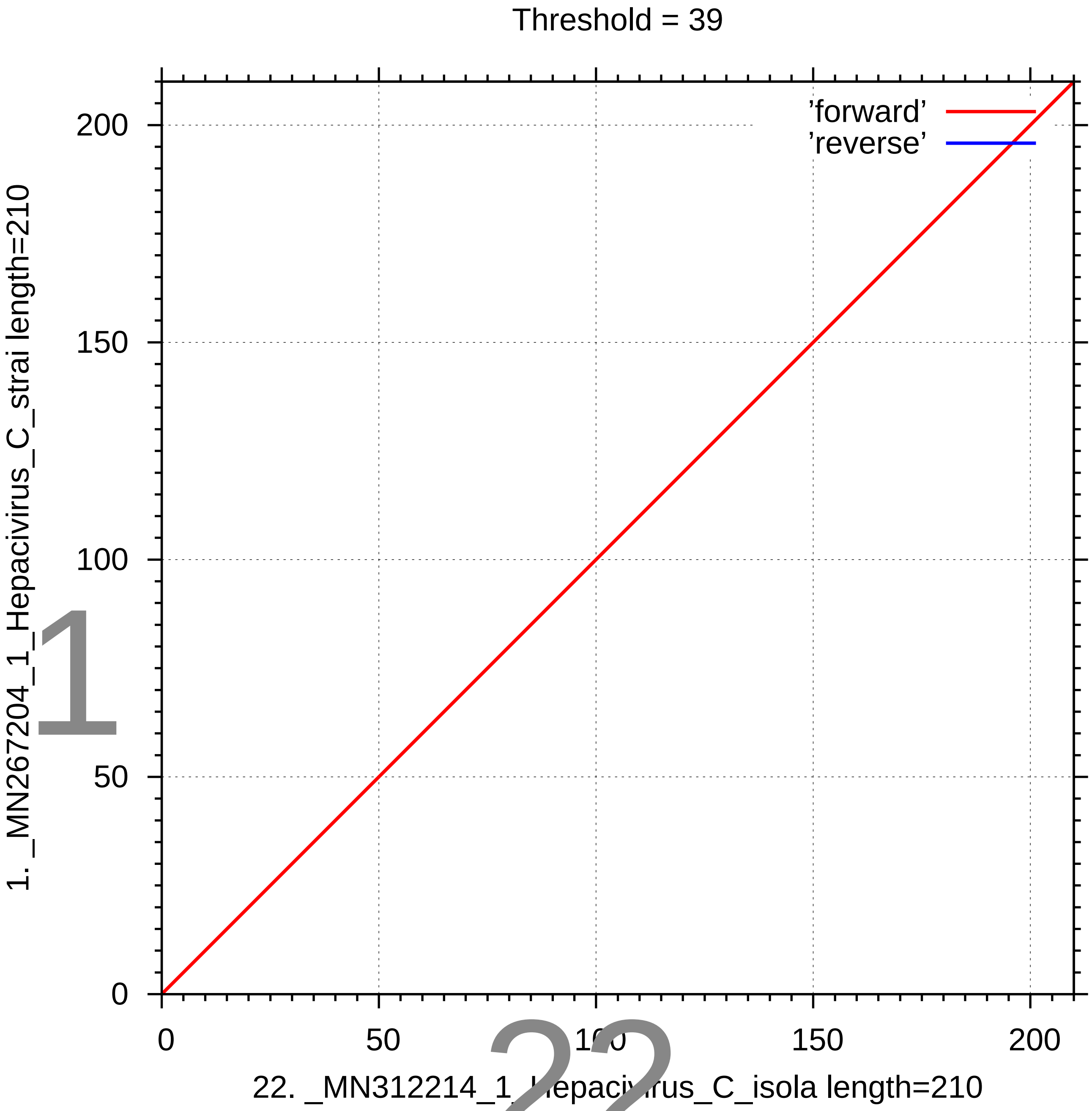

Threshold = 39
'forward'
'reverse'
0
50
100
150
200
0
50
100
150
200
1. _MN267204_1_Hepacivirus_C_strai length=210
22. _MN312214_1_Hepacivirus_C_isola length=210

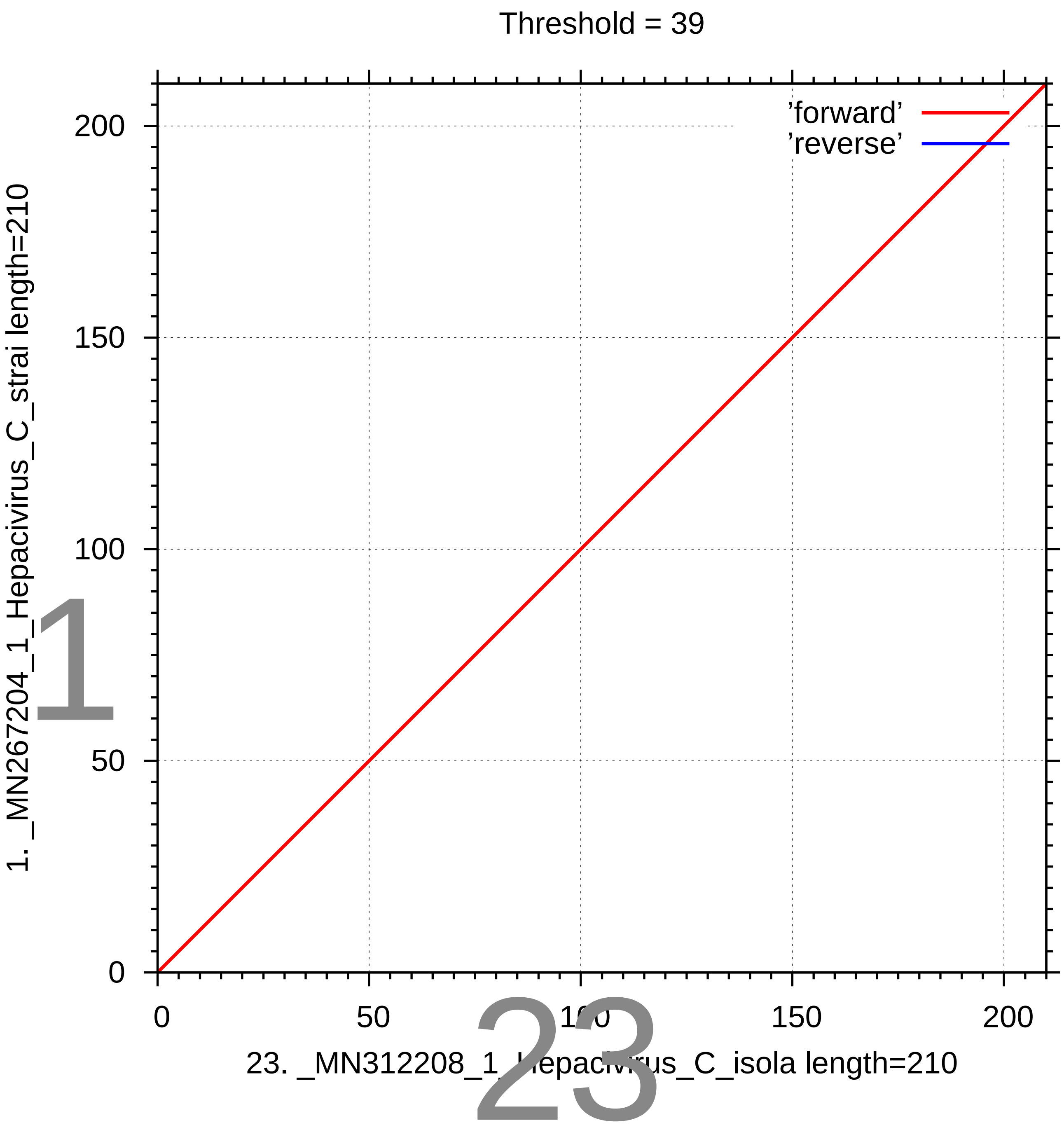

Threshold = 39
'forward'
'reverse'
0
50
100
150
200
1. _MN267204_1_Hepacivirus_C_strai length=210
23. _MN312208_1_Hepacivirus_C_isola length=210
1
23

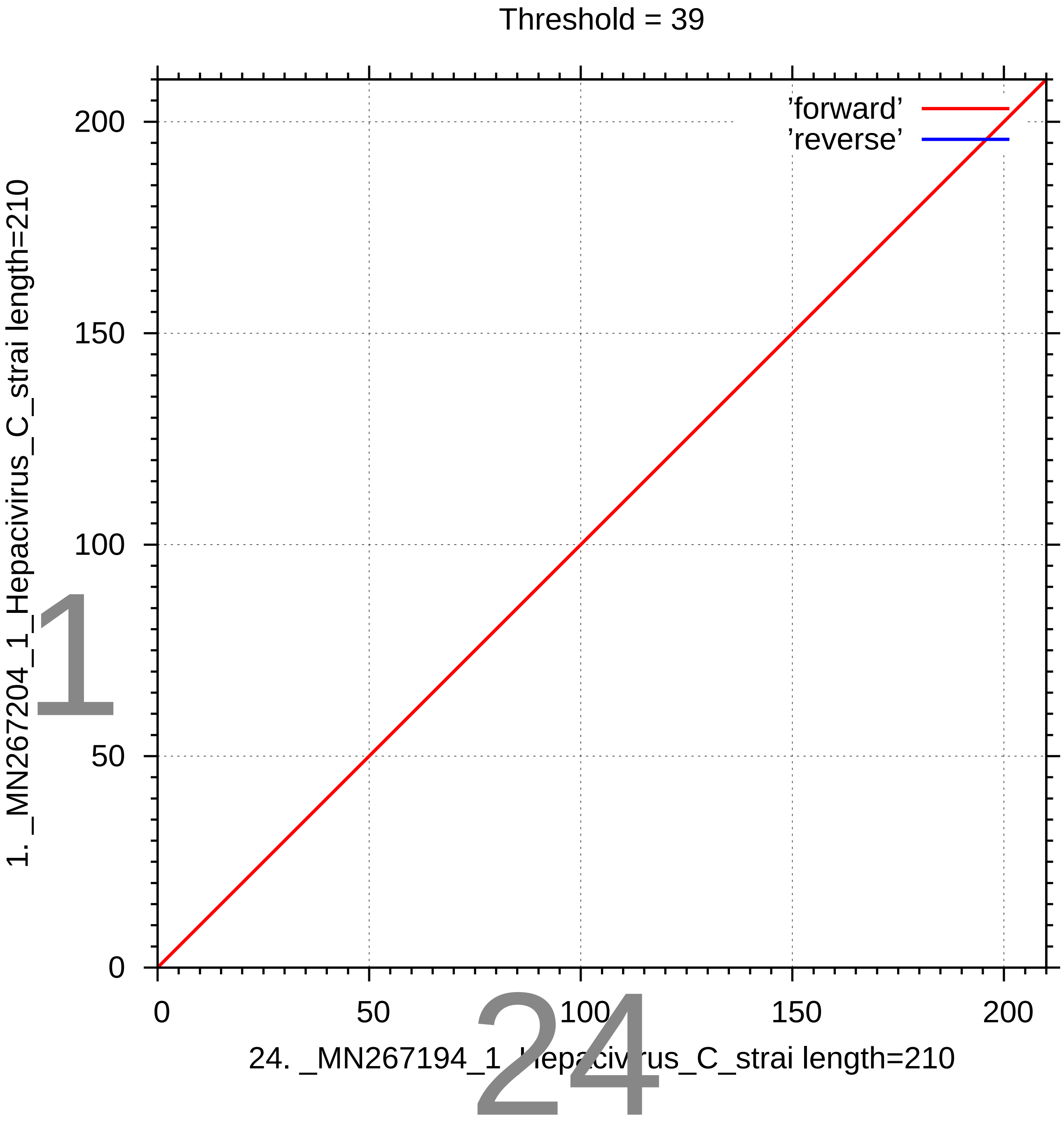
Threshold = 39
'forward'
'reverse'
1. _MN267204_1_Hepacivirus_C_strai length=210
24. _MN267194_1_Hepacivirus_C_strai length=210
0
50
100
150
200
0
50
100
150
200
1
24

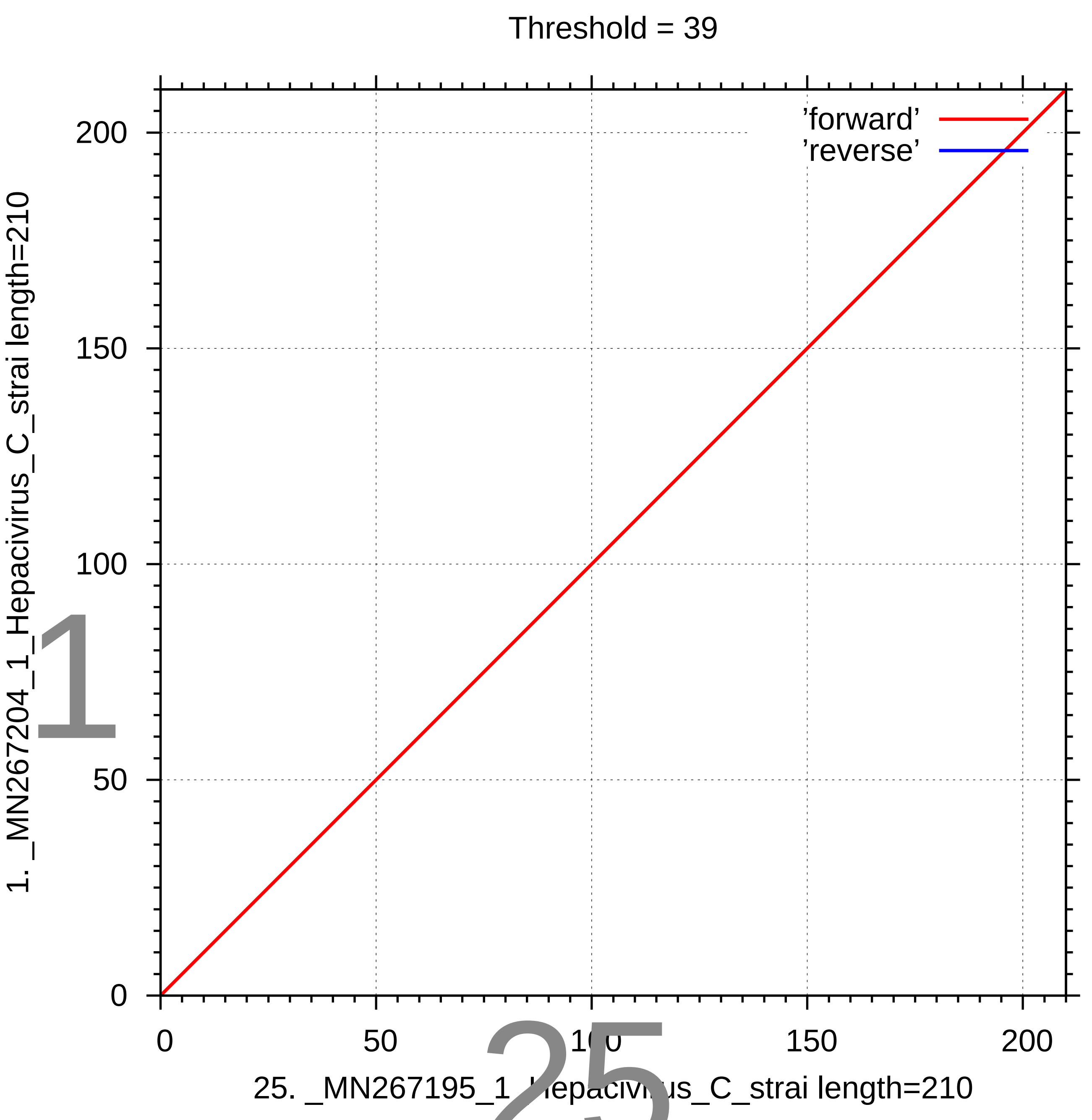
Threshold = 39
'forward'
'reverse'
0
50
100
150
200
0
50
150
200
25. _MN267195_1_Hepacivirus_C_strai length=210
1. _MN267204_1_Hepacivirus_C_strai length=210

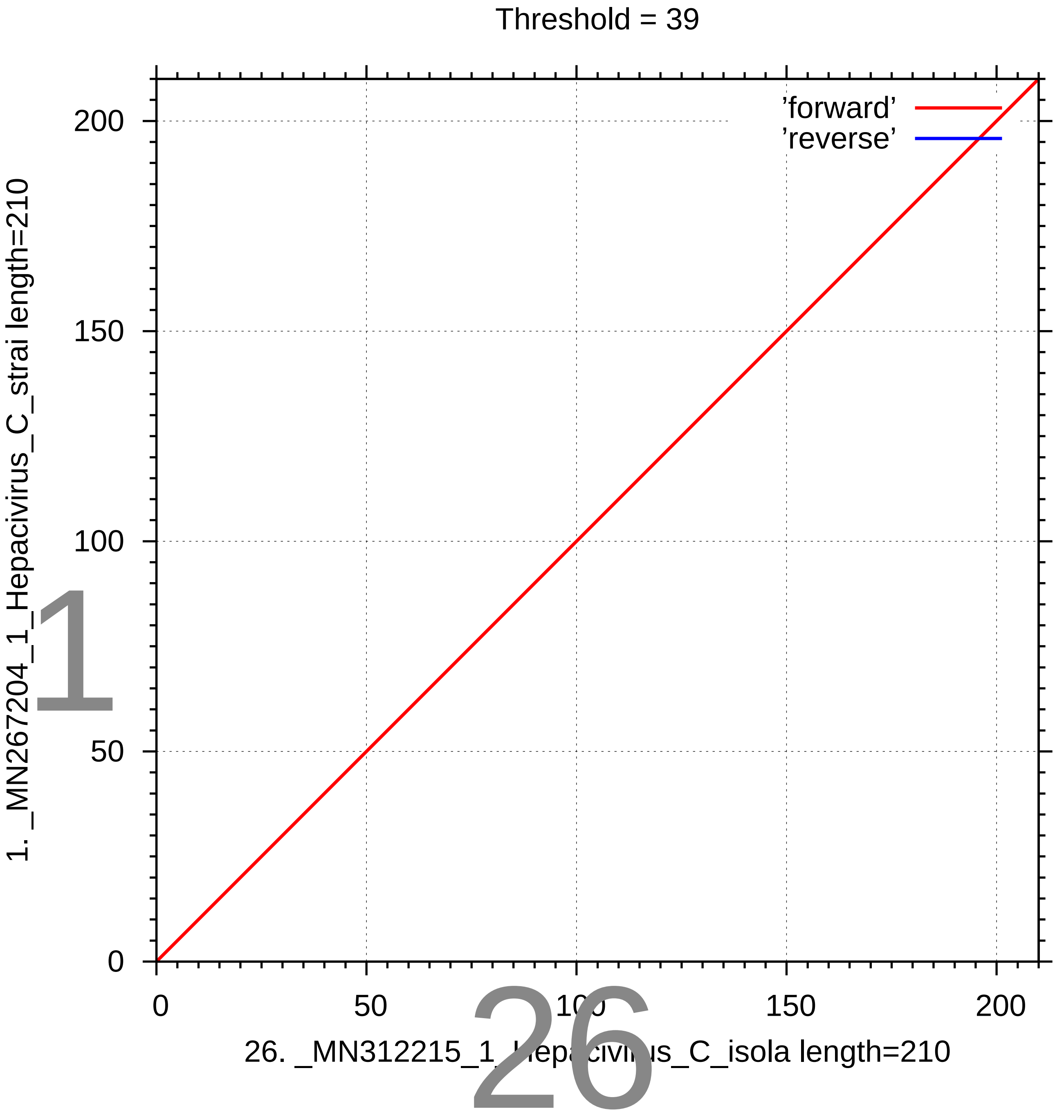
Threshold = 39
'forward'
'reverse'
1. _MN267204_1_Hepacivirus_C_strai length=210
26. _MN312215_1_Hepacivirus_C_isola length=210
0
50
100
150
200
1
26

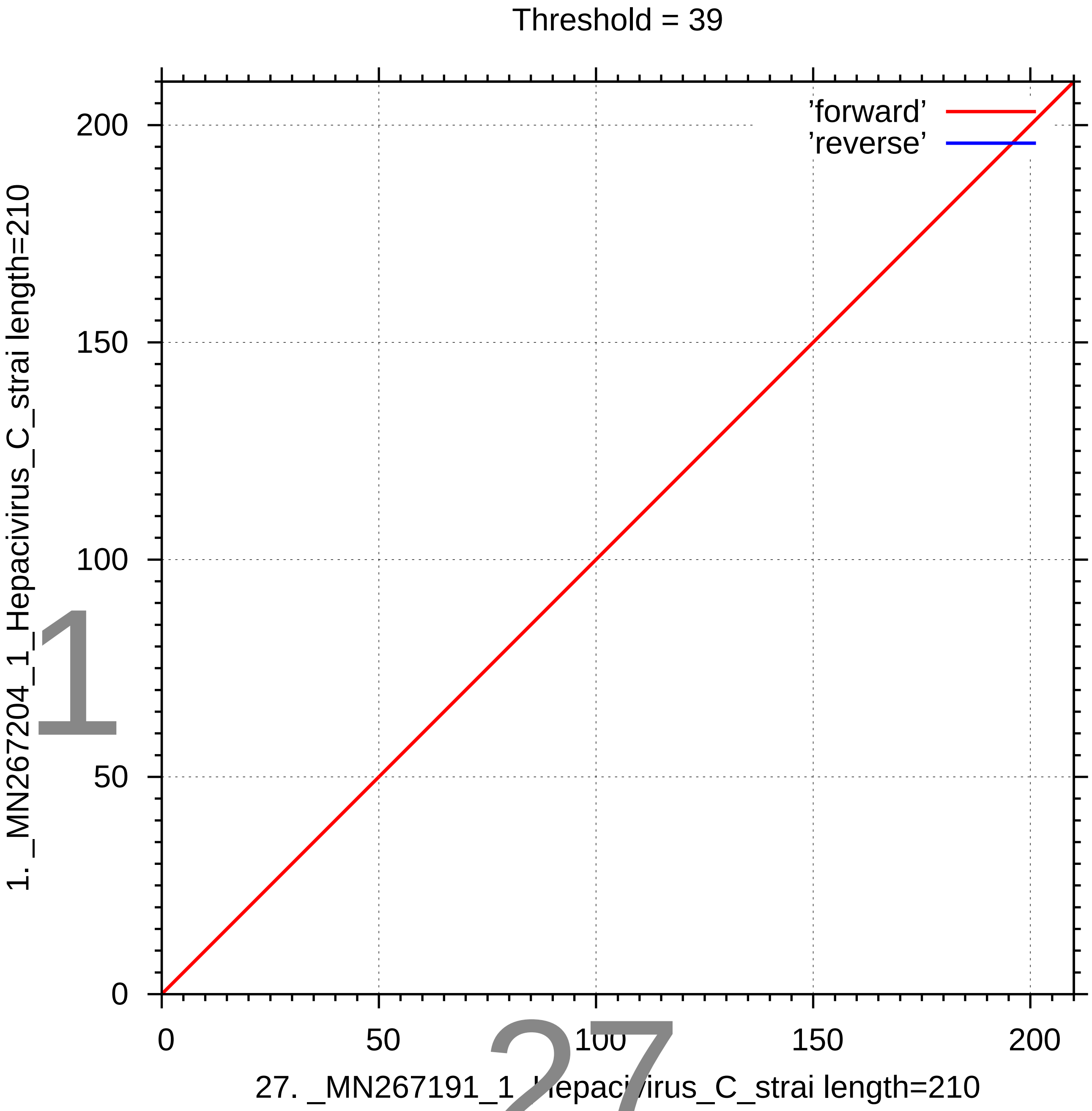

Threshold = 39
'forward'
'reverse'
0
50
100
150
200
1. _MN267204_1_Hepacivirus_C_strai length=210
27. _MN267191_1_Hepacivirus_C_strai length=210

1
27

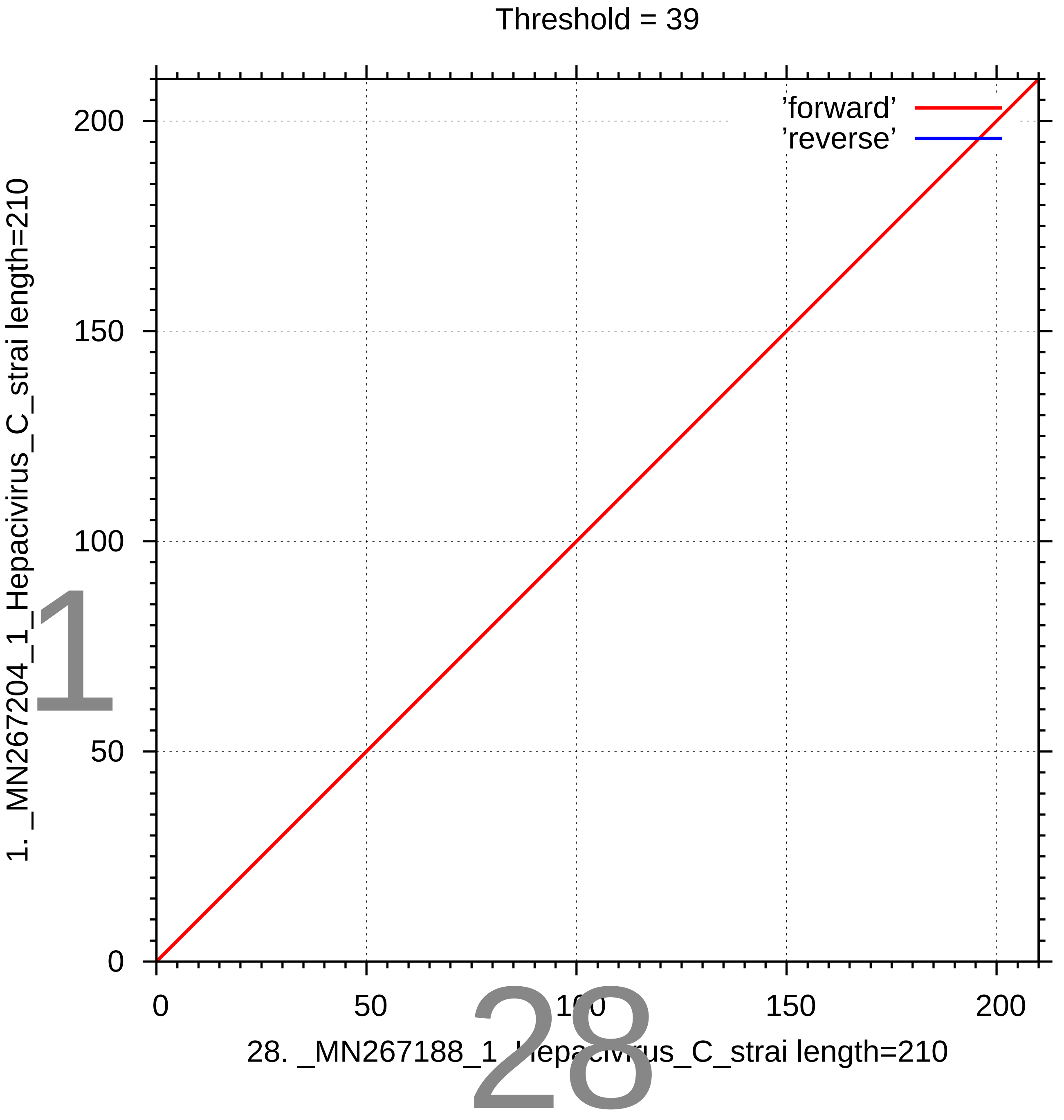

Threshold = 39
'forward'
'reverse'
0
50
100
150
200
1. _MN267204_1_Hepacivirus_C_strai length=210
28. _MN267188_1_Hepacivirus_C_strai length=210
1
28

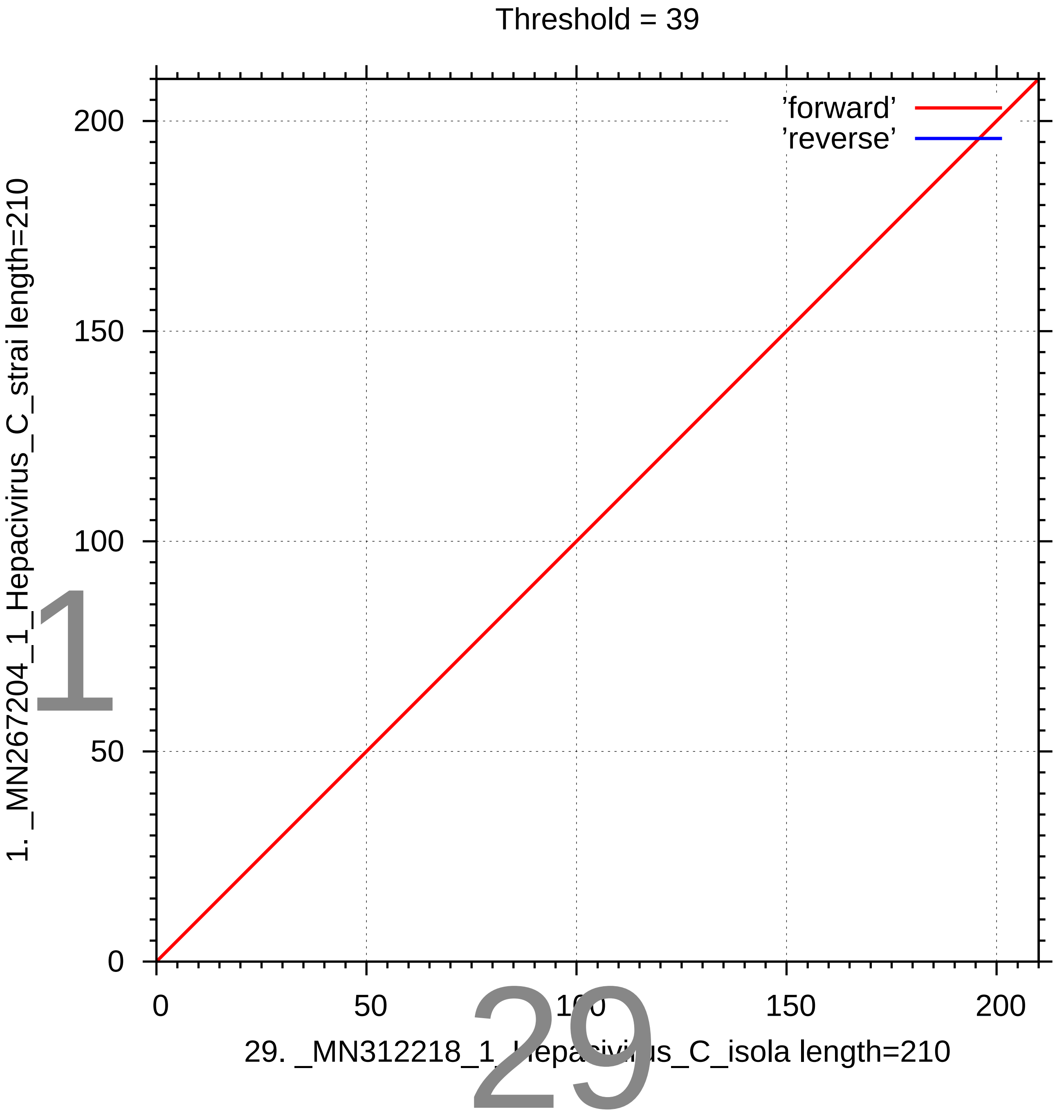

Threshold = 39
'forward'
'reverse'
200
150
100
50
0
0
50
100
150
200
1. _MN267204_1_Hepacivirus_C_strai length=210
29. _MN312218_1_Hepacivirus_C_isola length=210
1
29

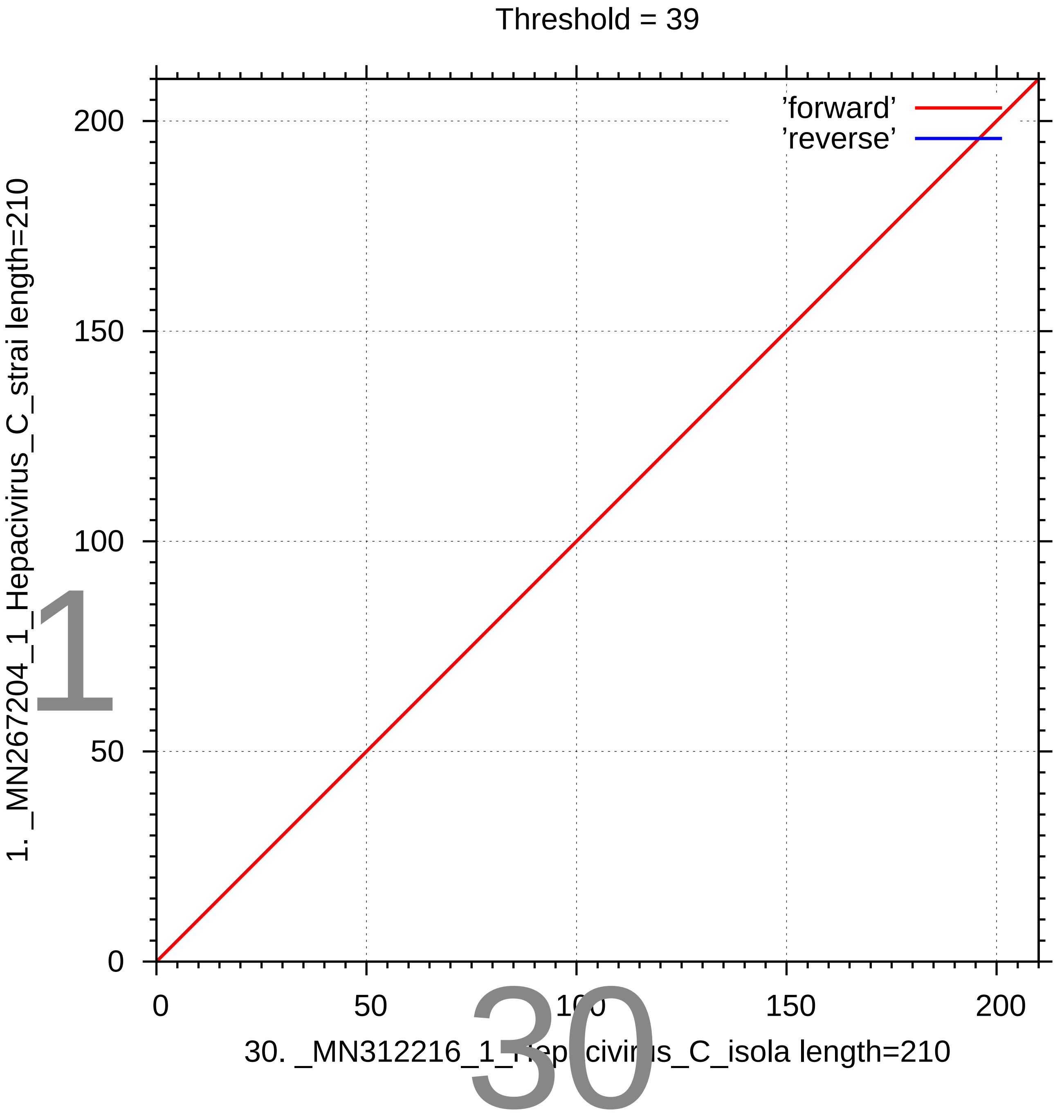
Threshold = 39
'forward'
'reverse'
0
50
100
150
200
1. _MN267204_1_Hepacivirus_C_strai length=210
30. _MN312216_1_Hepacivirus_C_isola length=210
1
30

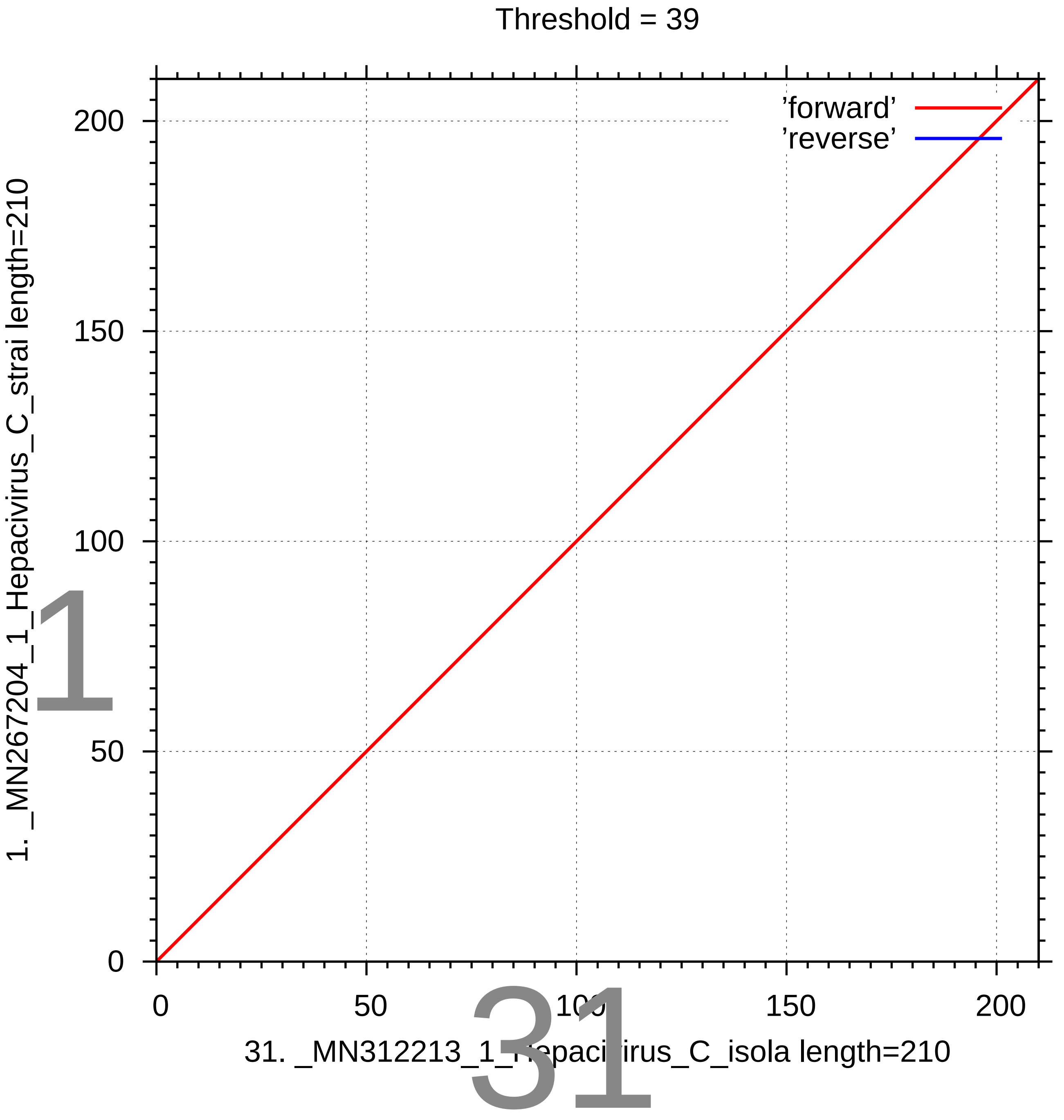

Threshold = 39
'forward'
'reverse'
1. _MN267204_1_Hepacivirus_C_strai length=210
31. _MN312213_1_Hepacivirus_C_isola length=210
0
50
100
150
200
1
31

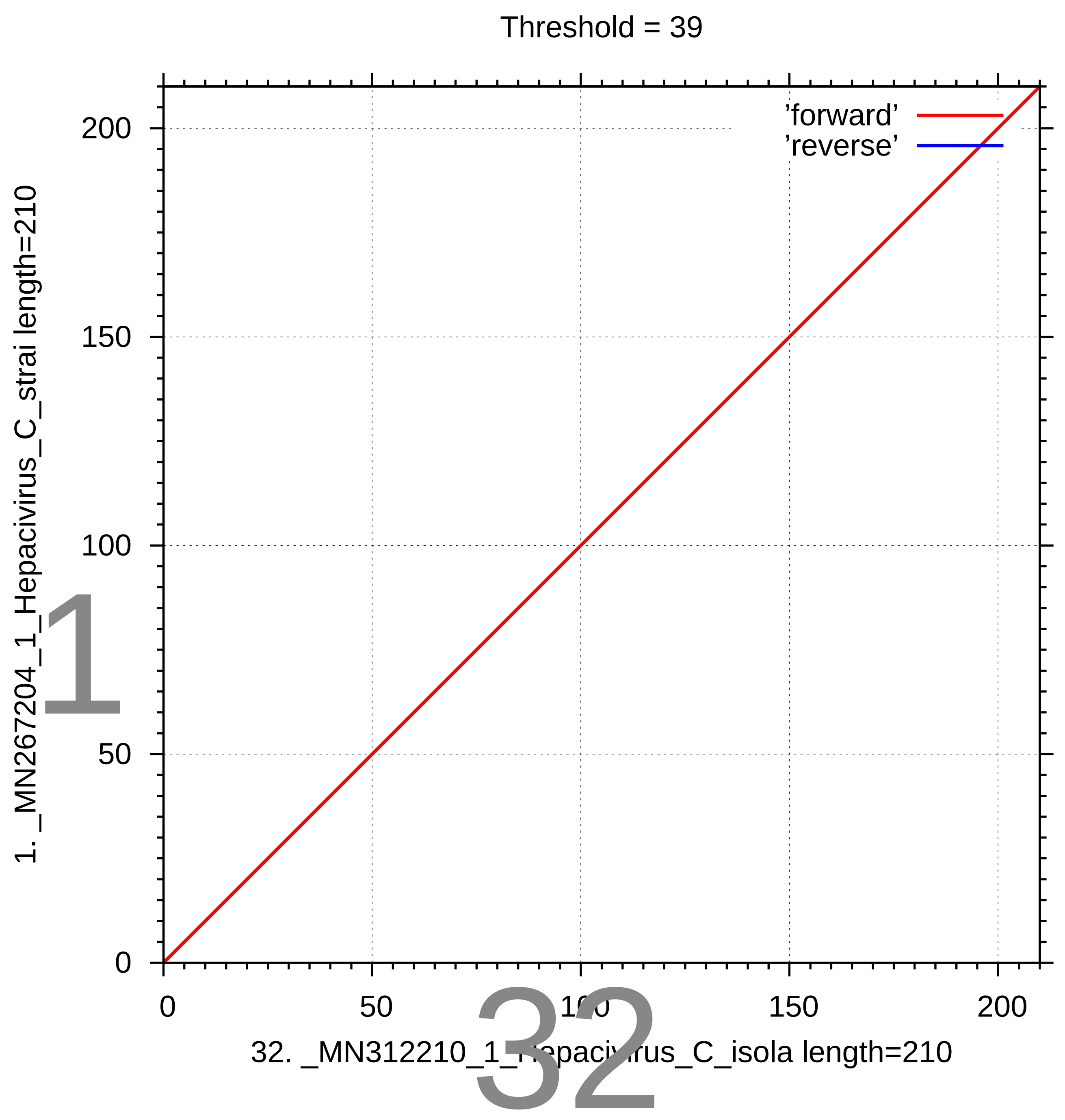

Threshold = 39
'forward'
'reverse'
0
50
100
150
200
1. _MN267204_1_Hepacivirus_C_strai length=210
32. _MN312210_1_Hepacivirus_C_isola length=210
1
32

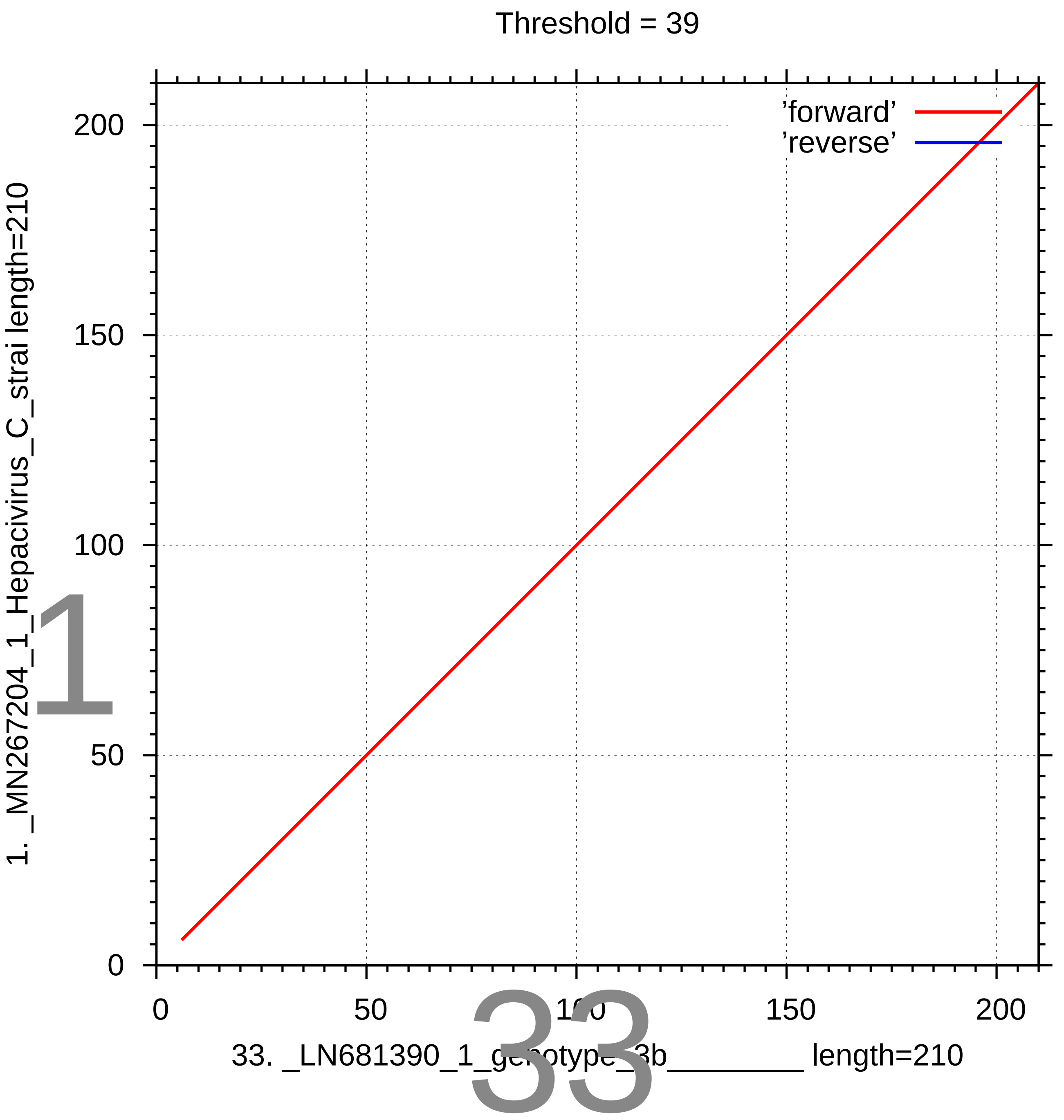

Threshold = 39
'forward'
'reverse'
1. _MN267204_1_Hepacivirus_C_strai length=210
33. _LN681390_1_genotype_3b________ length=210
0
50
100
150
200
1
33

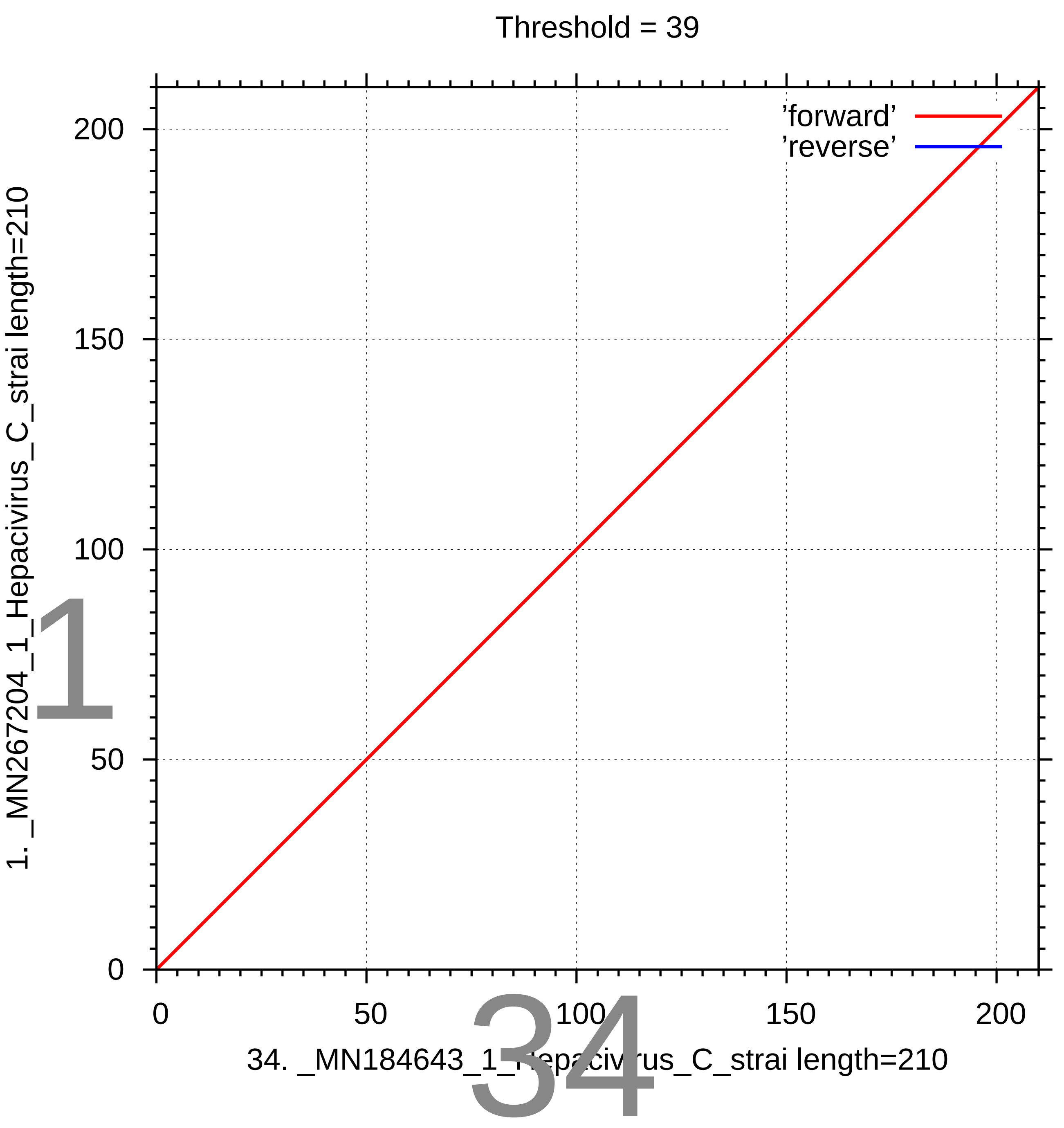
Threshold = 39
'forward'
'reverse'
1. _MN267204_1_Hepacivirus_C_strai length=210
34. _MN184643_1_Hepacivirus_C_strai length=210
0
50
100
150
200
1
34

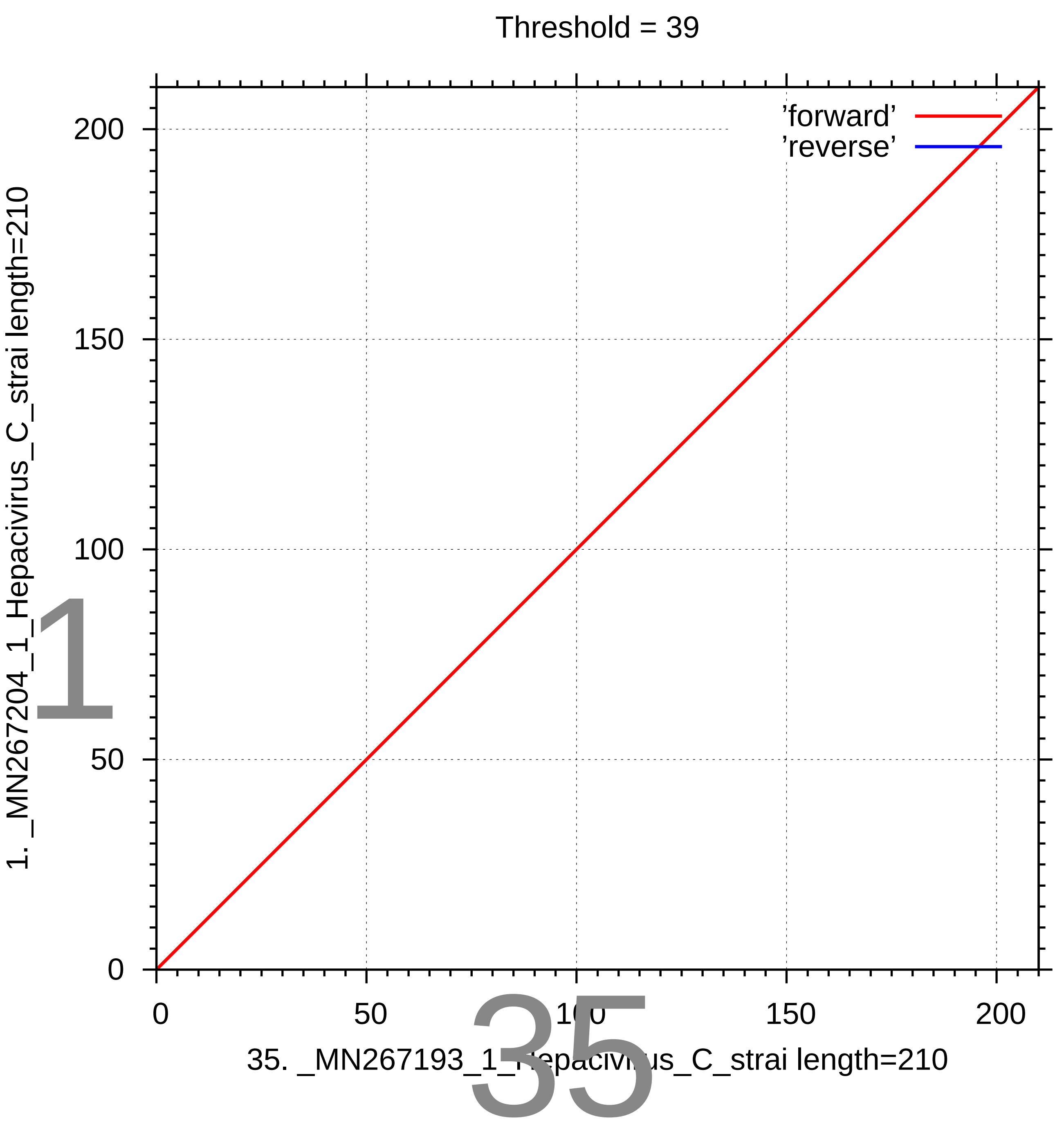
Threshold = 39
'forward'
'reverse'
0
50
100
150
200
0
50
100
150
200
1. _MN267204_1_Hepacivirus_C_strai length=210
35. _MN267193_1_Hepacivirus_C_strai length=210
1
35

Threshold = 39

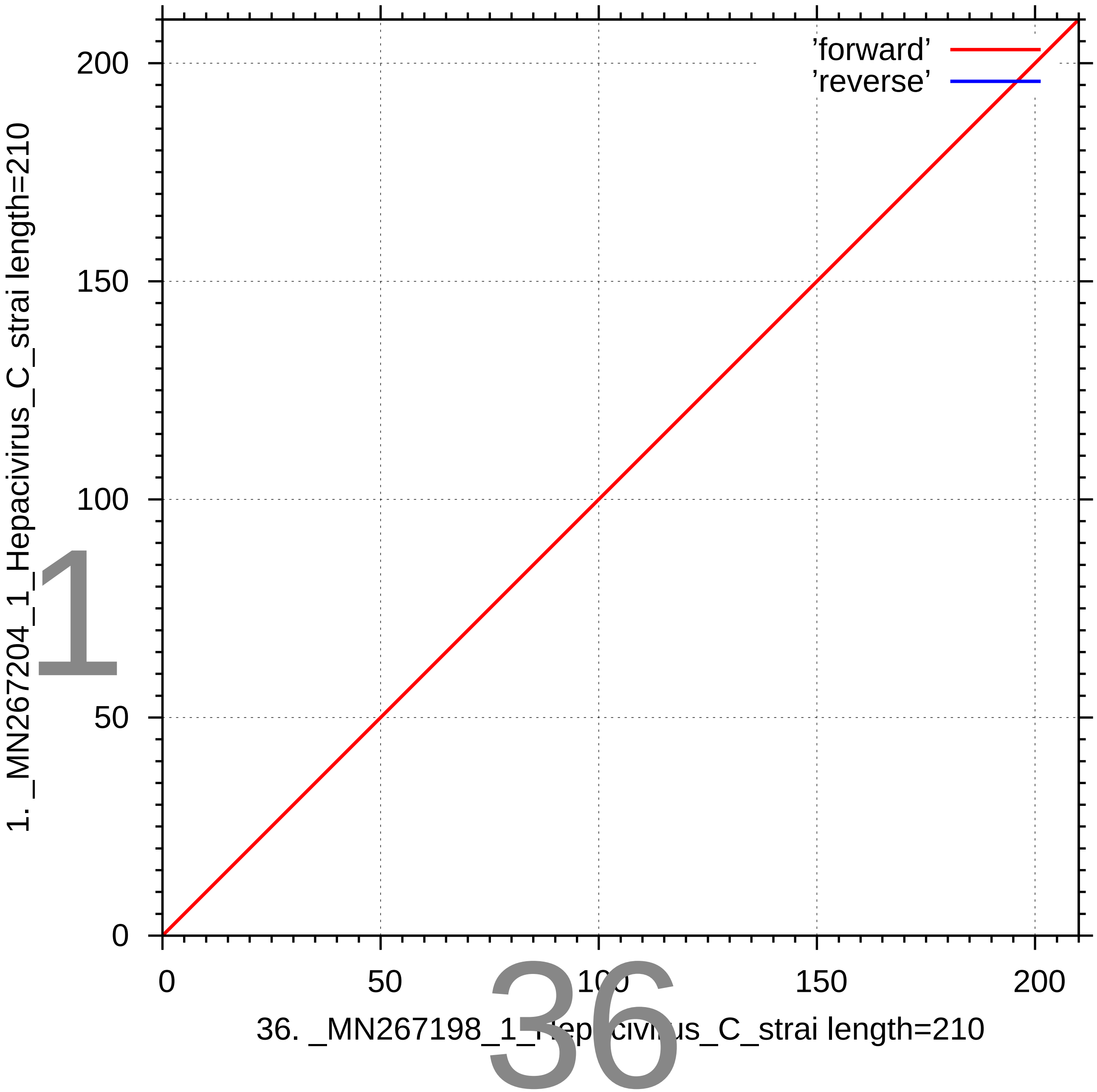

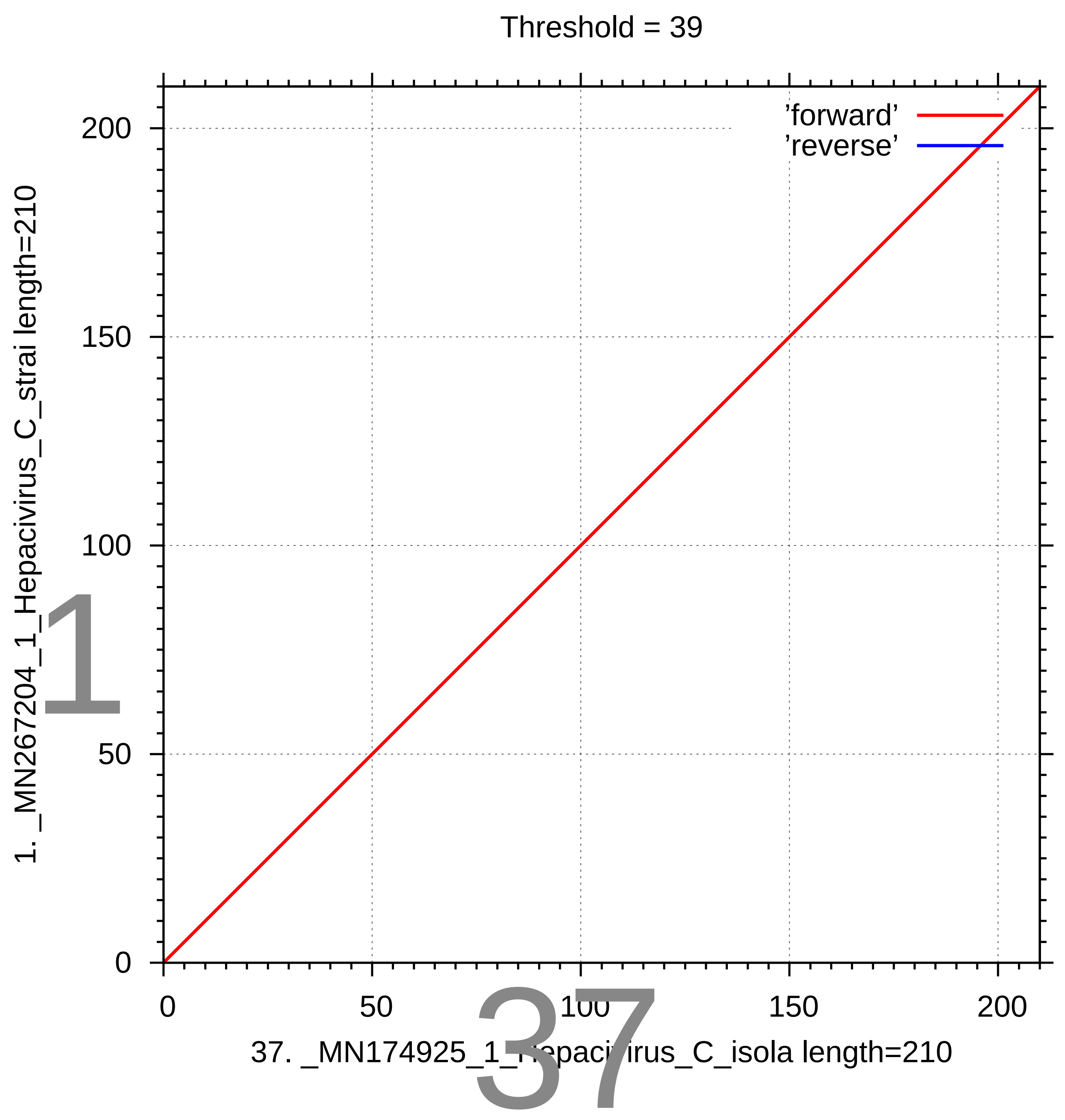
Threshold = 39
'forward'
'reverse'
200
150
100
50
0
0
50
100
150
200
1. _MN267204_1_Hepacivirus_C_strai length=210
37. _MN174925_1_Hepacivirus_C_isola length=210
1
37

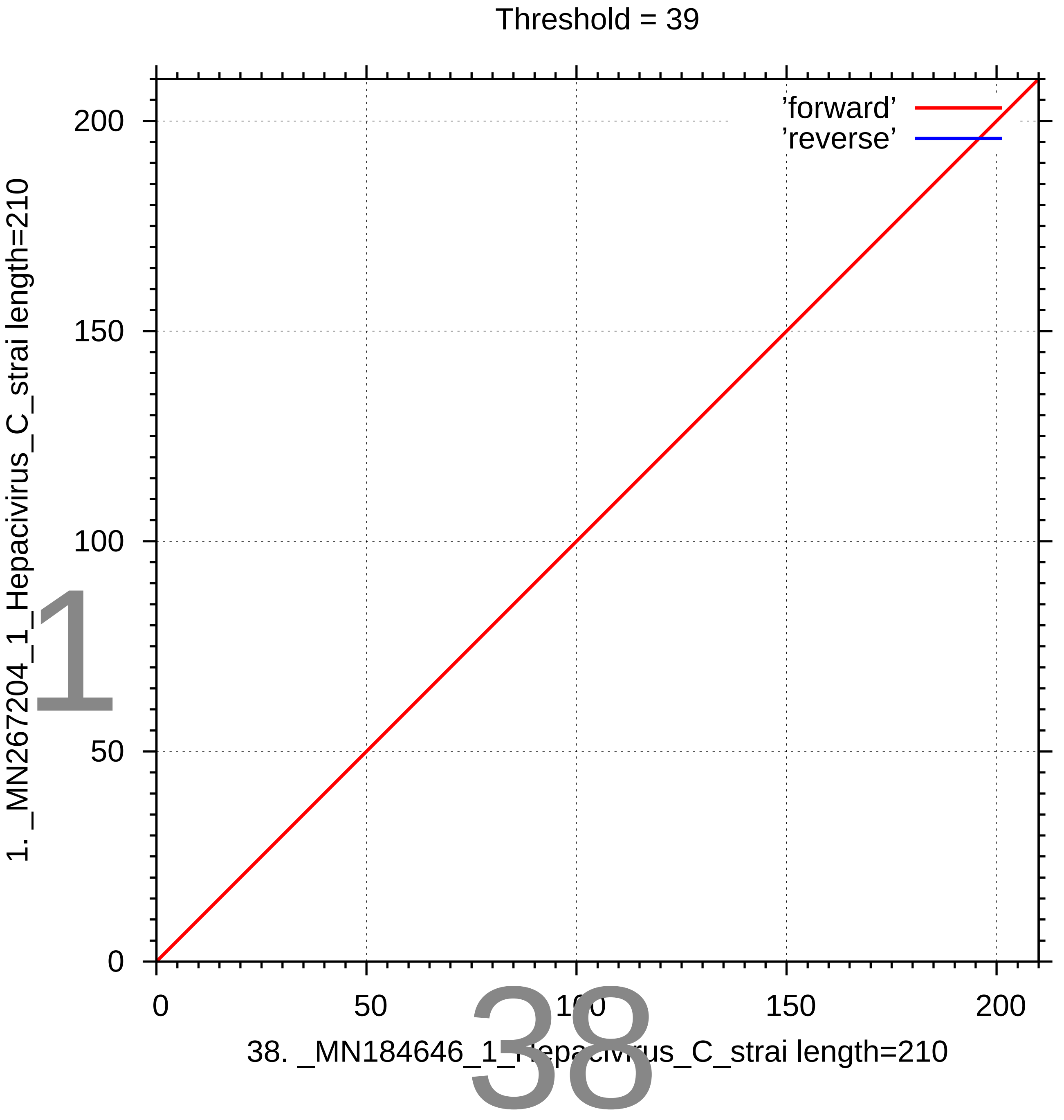

Threshold = 39
'forward'
'reverse'
0
50
100
150
200
1. _MN267204_1_Hepacivirus_C_strai length=210
38. _MN184646_1_Hepacivirus_C_strai length=210
1
38

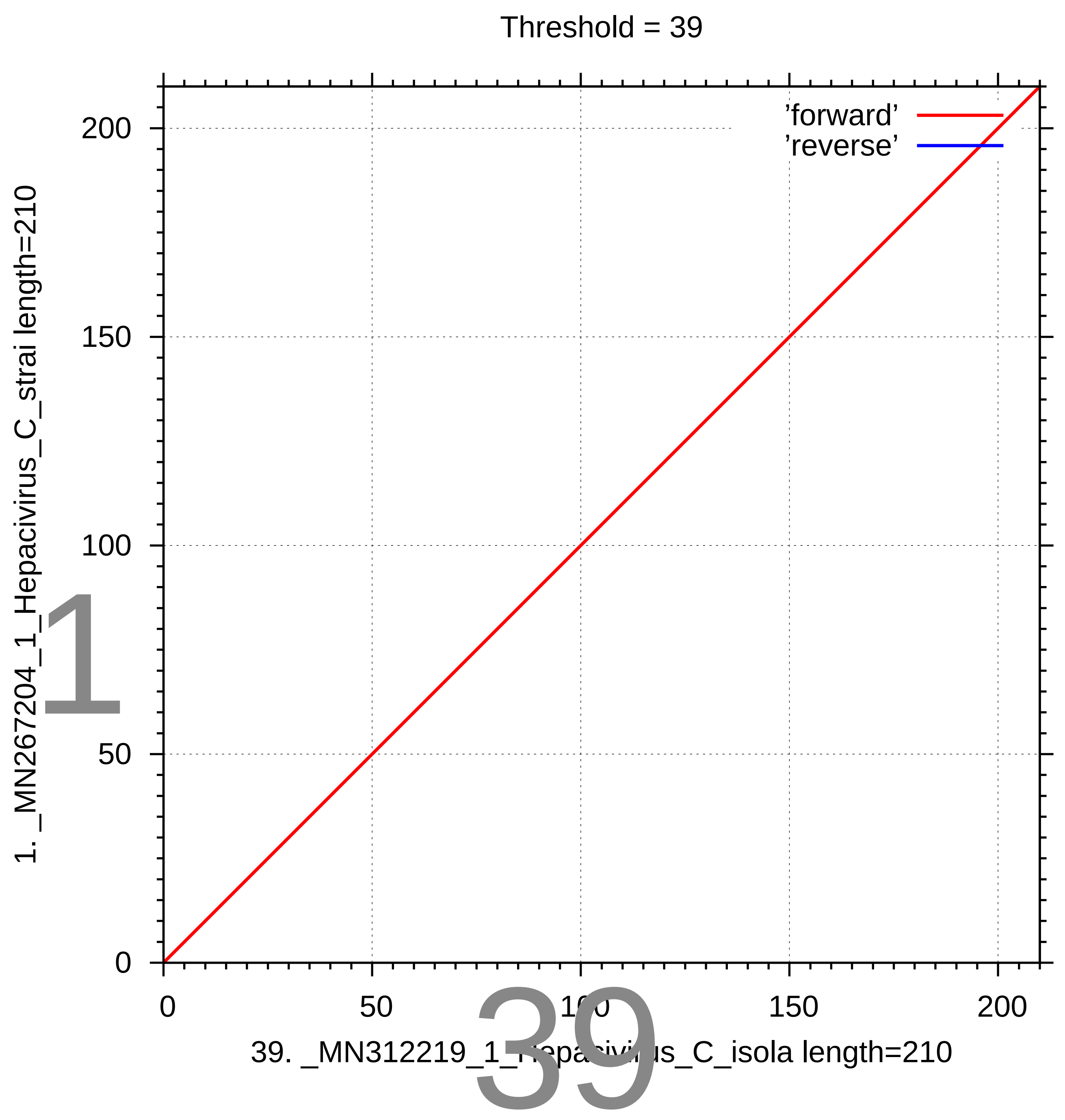
Threshold = 39
'forward'
'reverse'
0
50
100
150
200
0
50
100
150
200
1. _MN267204_1_Hepacivirus_C_strai length=210
39. _MN312219_1_Hepacivirus_C_isola length=210
1
39

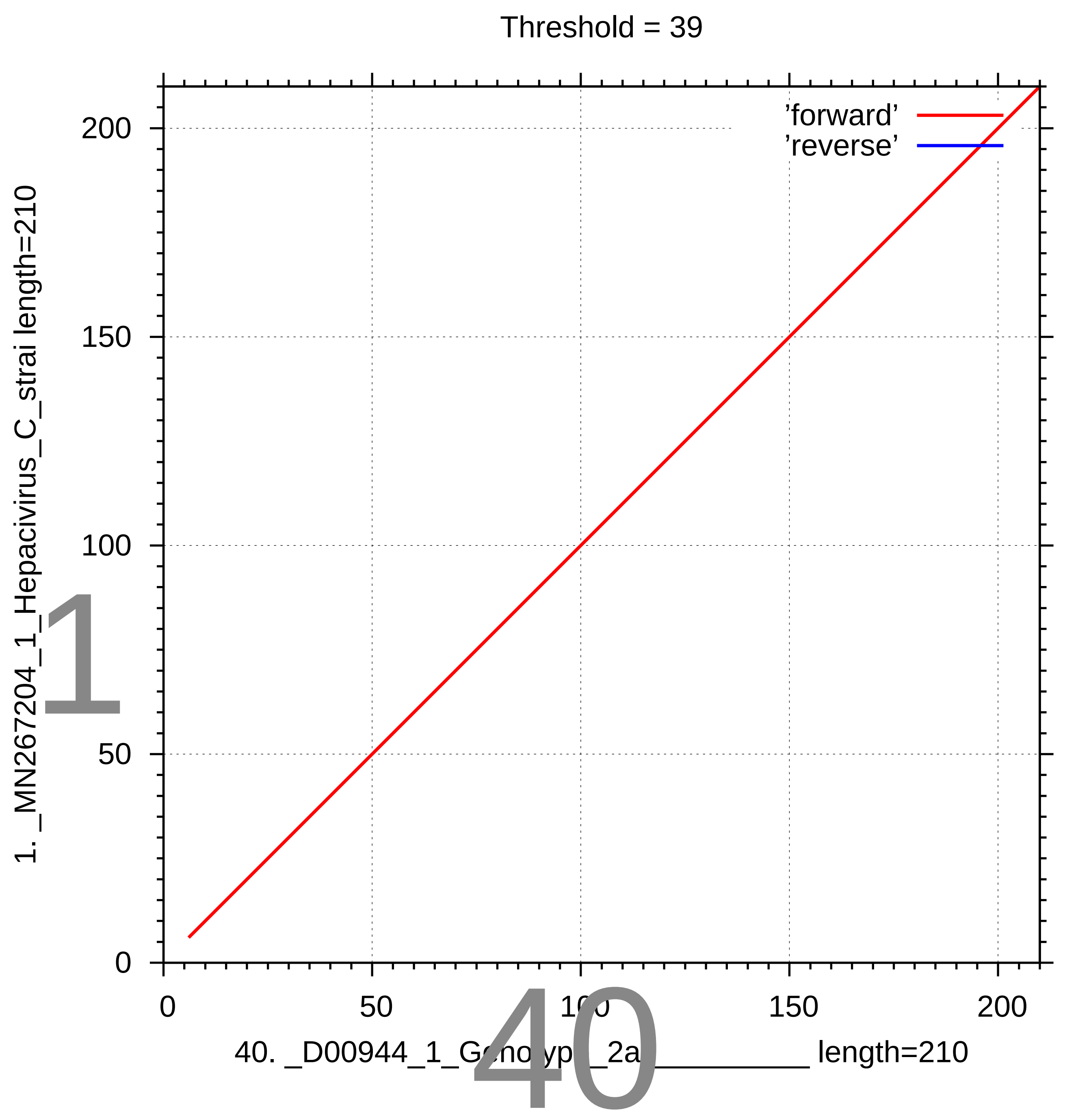

Threshold = 39
'forward'
'reverse'
0
50
100
150
200
1. _MN267204_1_Hepacivirus_C_strai length=210
length=210
1
40

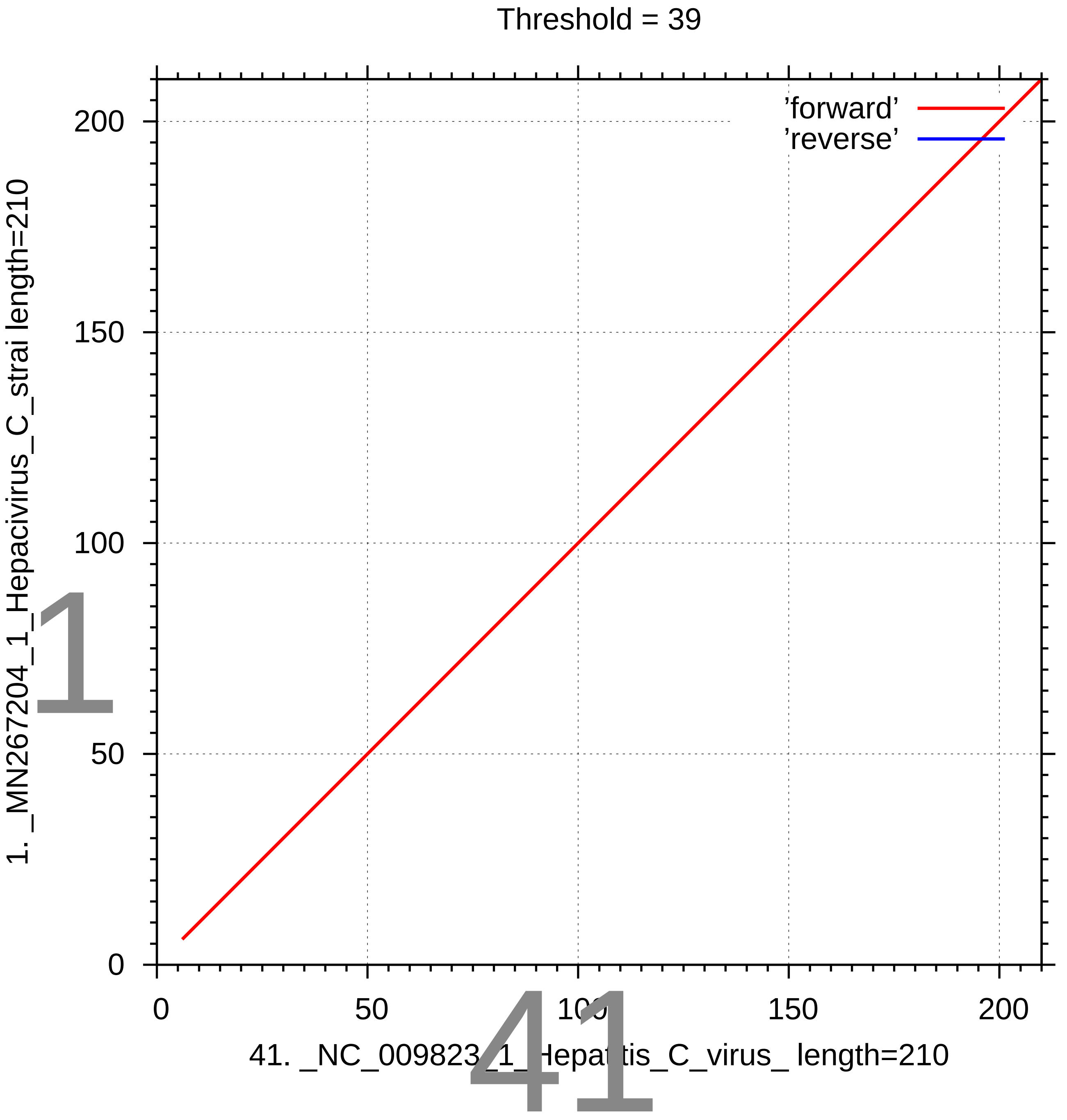

Threshold = 39
'forward'
'reverse'
0
50
100
150
200
1. _MN267204_1_Hepacivirus_C_strai length=210
41. _NC_009823_1_Hepatitis_C_virus_ length=210
1
41

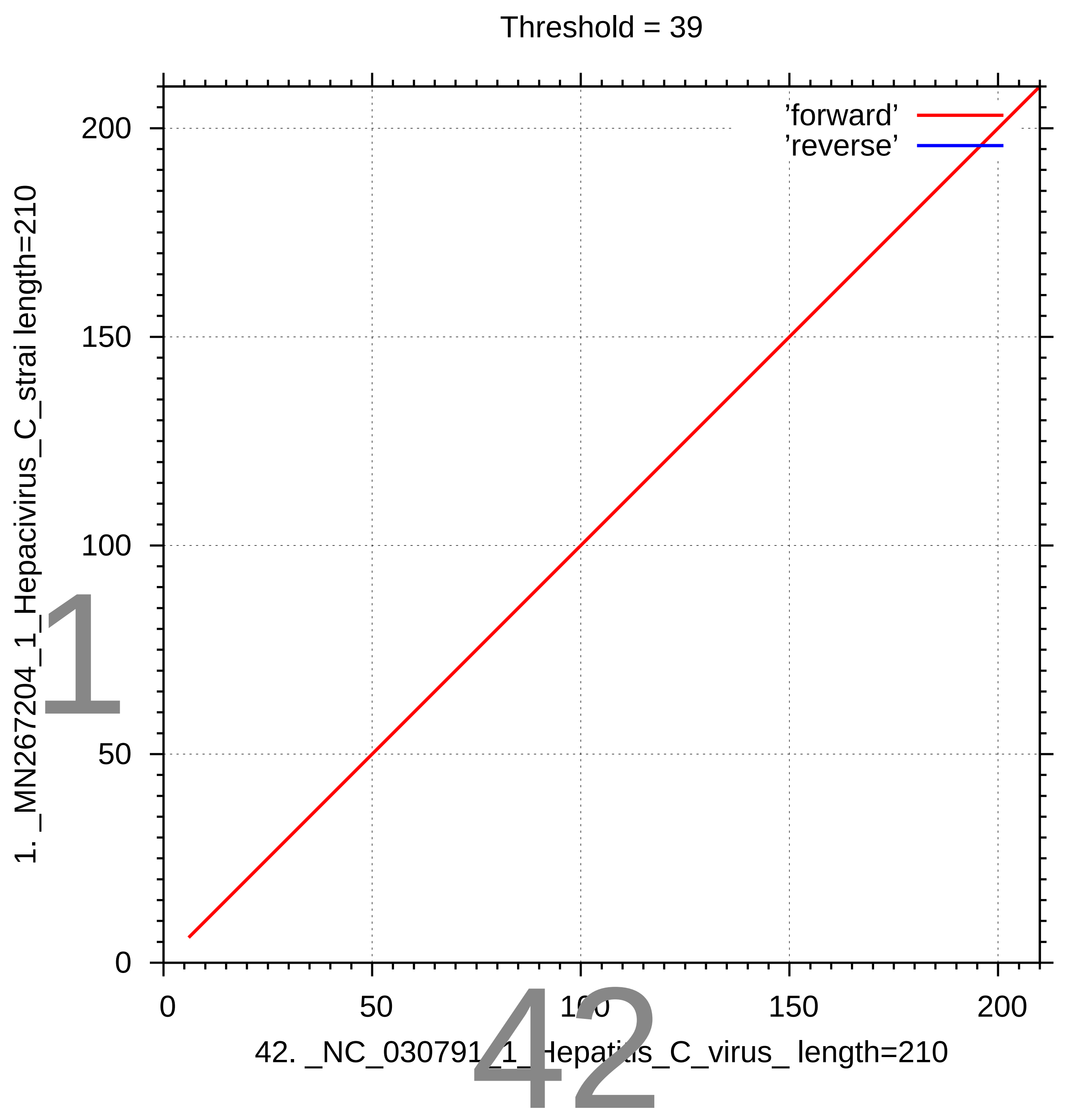

Threshold = 39
'forward'
'reverse'
0
50
100
150
200
1. _MN267204_1_Hepacivirus_C_strai length=210
42. _NC_030791_1_Hepatitis_C_virus_ length=210
1
42

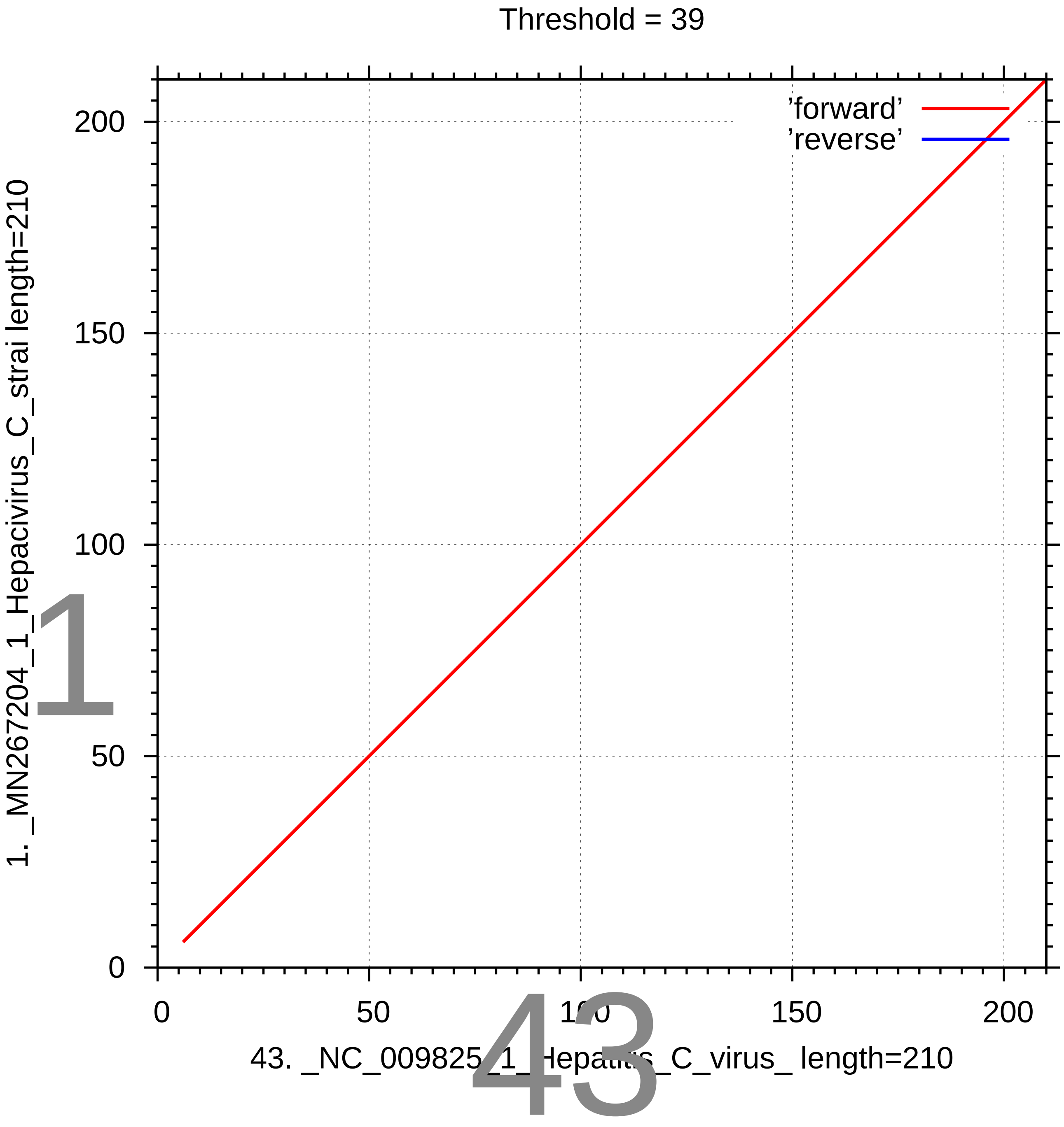
Threshold = 39
'forward'
'reverse'
0
50
100
150
200
43. _NC_009825_1_Hepatitis_C_virus_ length=210
1. _MN267204_1_Hepacivirus_C_strai length=210
1
43

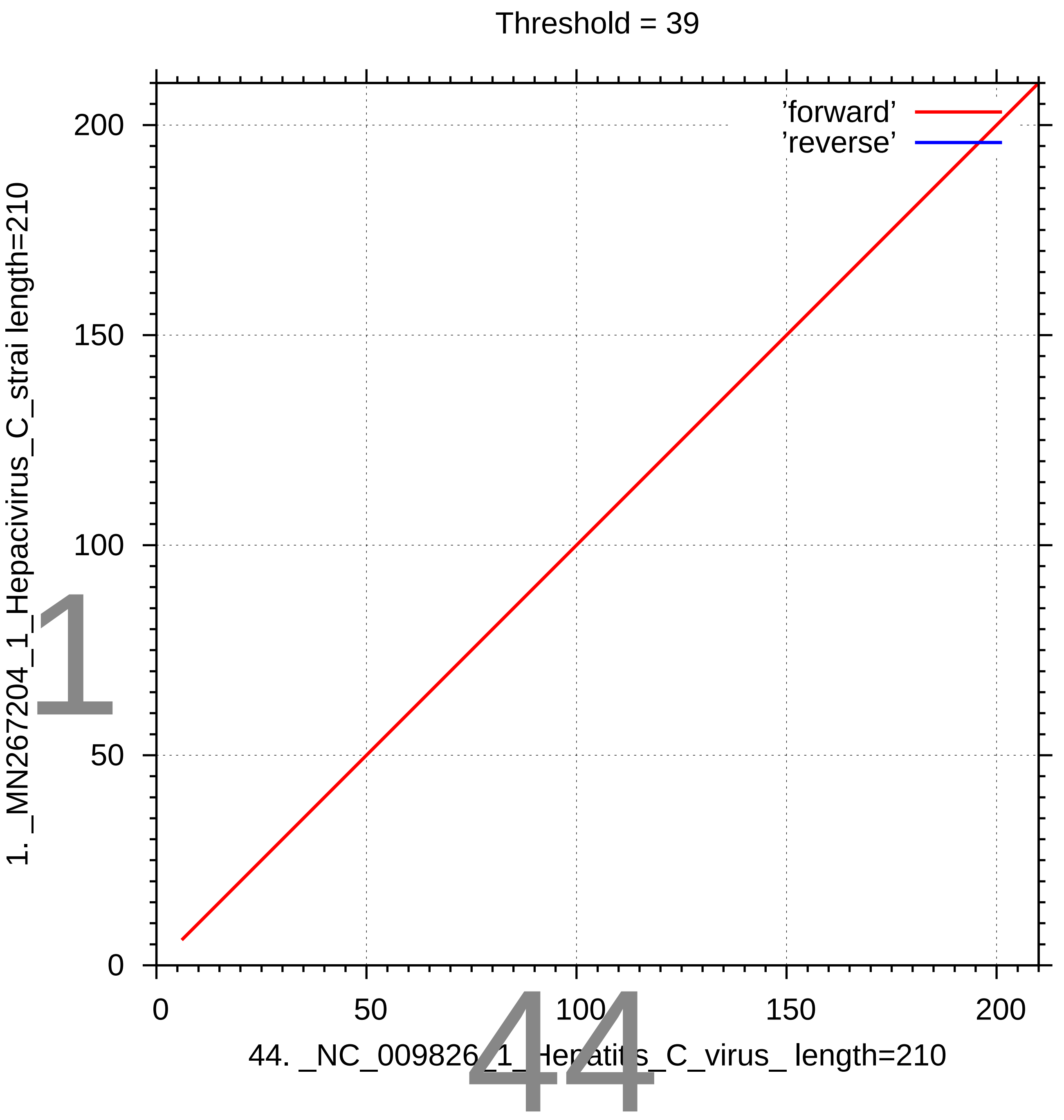
Threshold = 39
'forward'
'reverse'
0
50
100
150
200
1. _MN267204_1_Hepacivirus_C_strai length=210
44. _NC_009826_1_Hepatitis_C_virus_ length=210
1
44

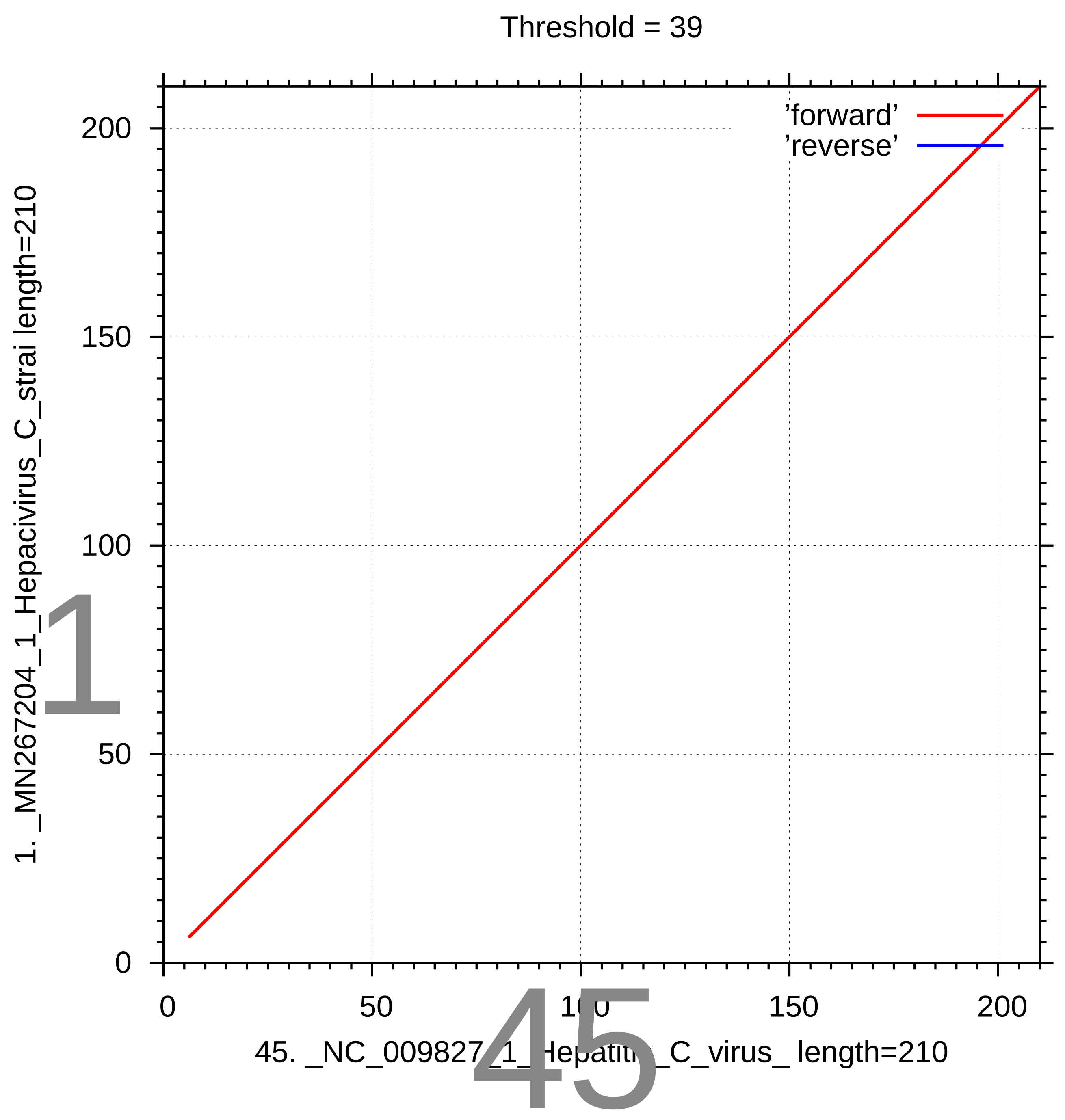

Threshold = 39
'forward'
'reverse'
1. _MN267204_1_Hepacivirus_C_strai length=210
45. _NC_009827_1_Hepatitis_C_virus_ length=210
0
50
100
150
200
1
45

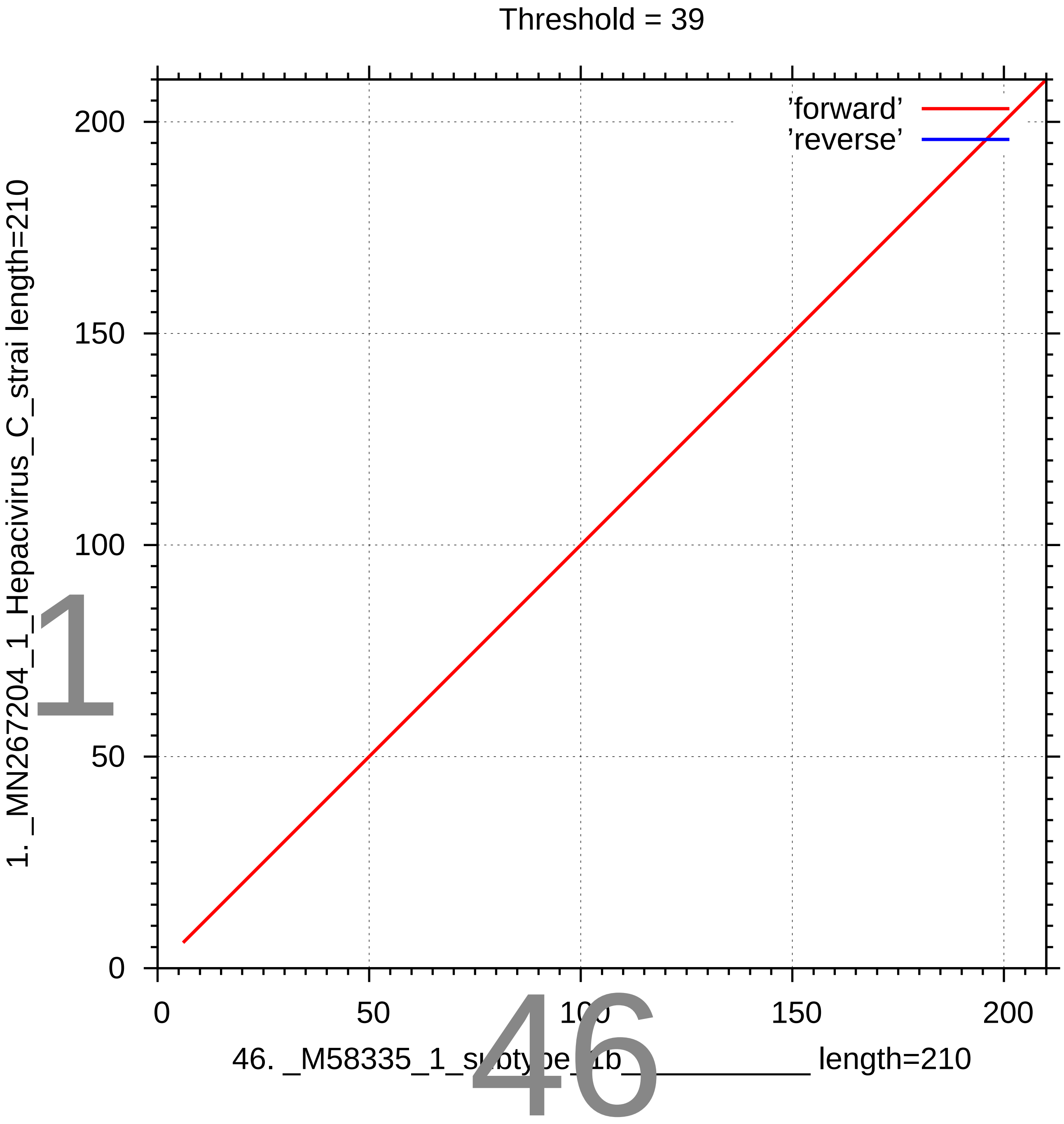
Threshold = 39
'forward'
'reverse'
1. _MN267204_1_Hepacivirus_C_strai length=210
46. _M58335_1_subtype_1b__________ length=210
0
50
100
150
200
1
46

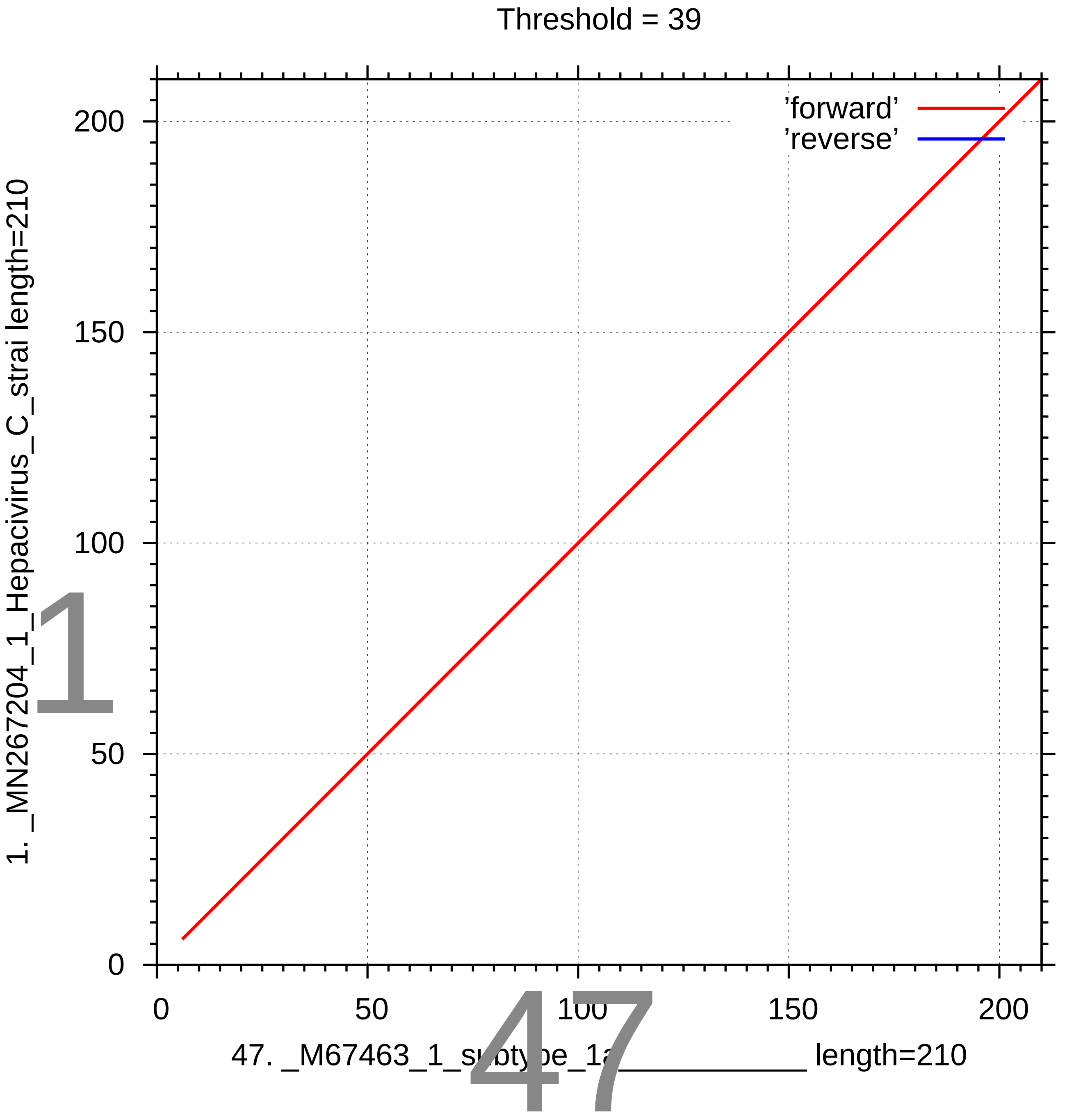
Threshold = 39
'forward'
'reverse'
0
50
100
150
200
1. _MN267204_1_Hepacivirus_C_strai length=210
47. _M67463_1_subtype_1a____________ length=210
1
47

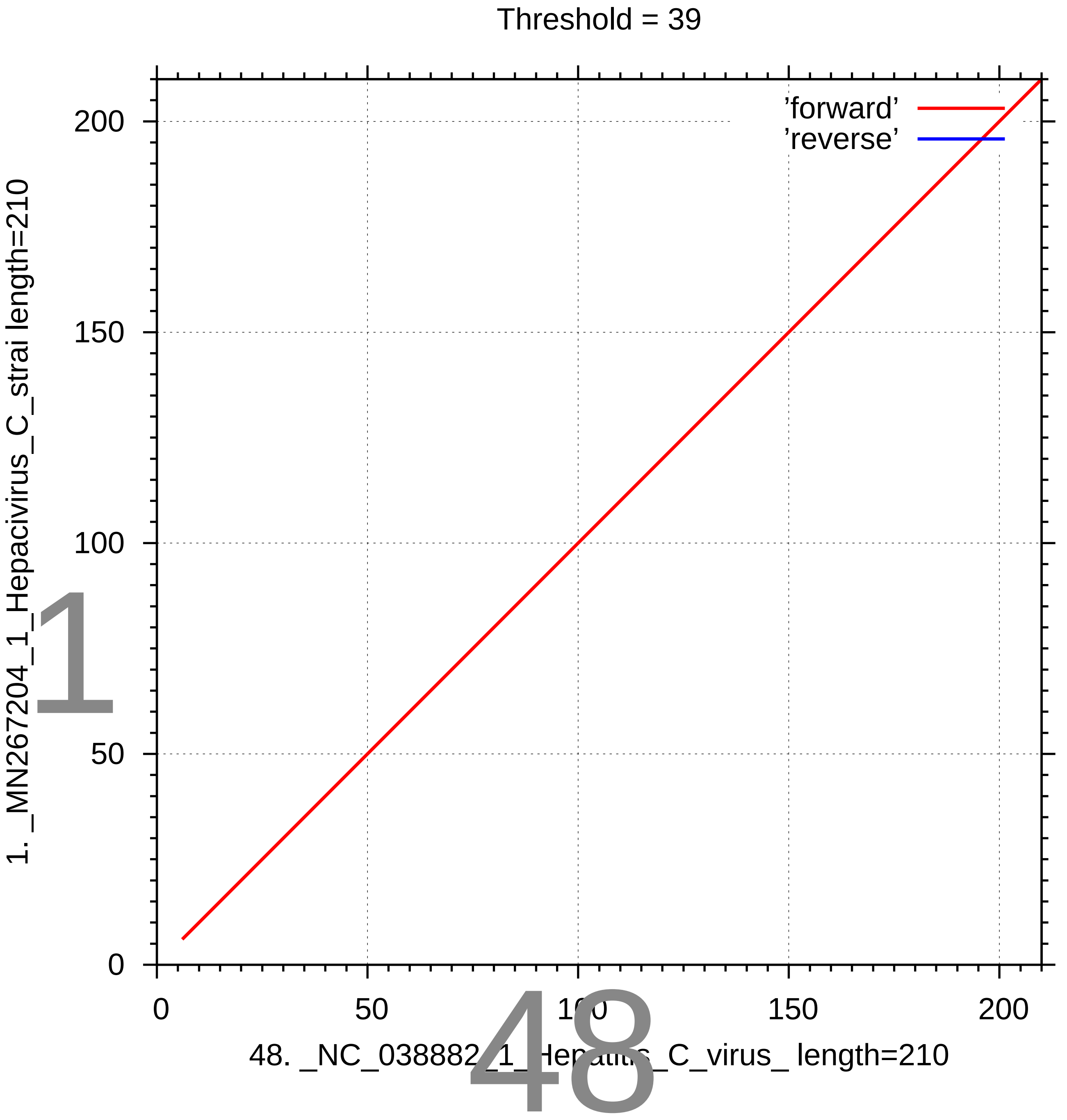
Threshold = 39
'forward'
'reverse'
1. _MN267204_1_Hepacivirus_C_strai length=210
48. _NC_038882_1_Hepatitis_C_virus_ length=210
0
50
100
150
200
1
48

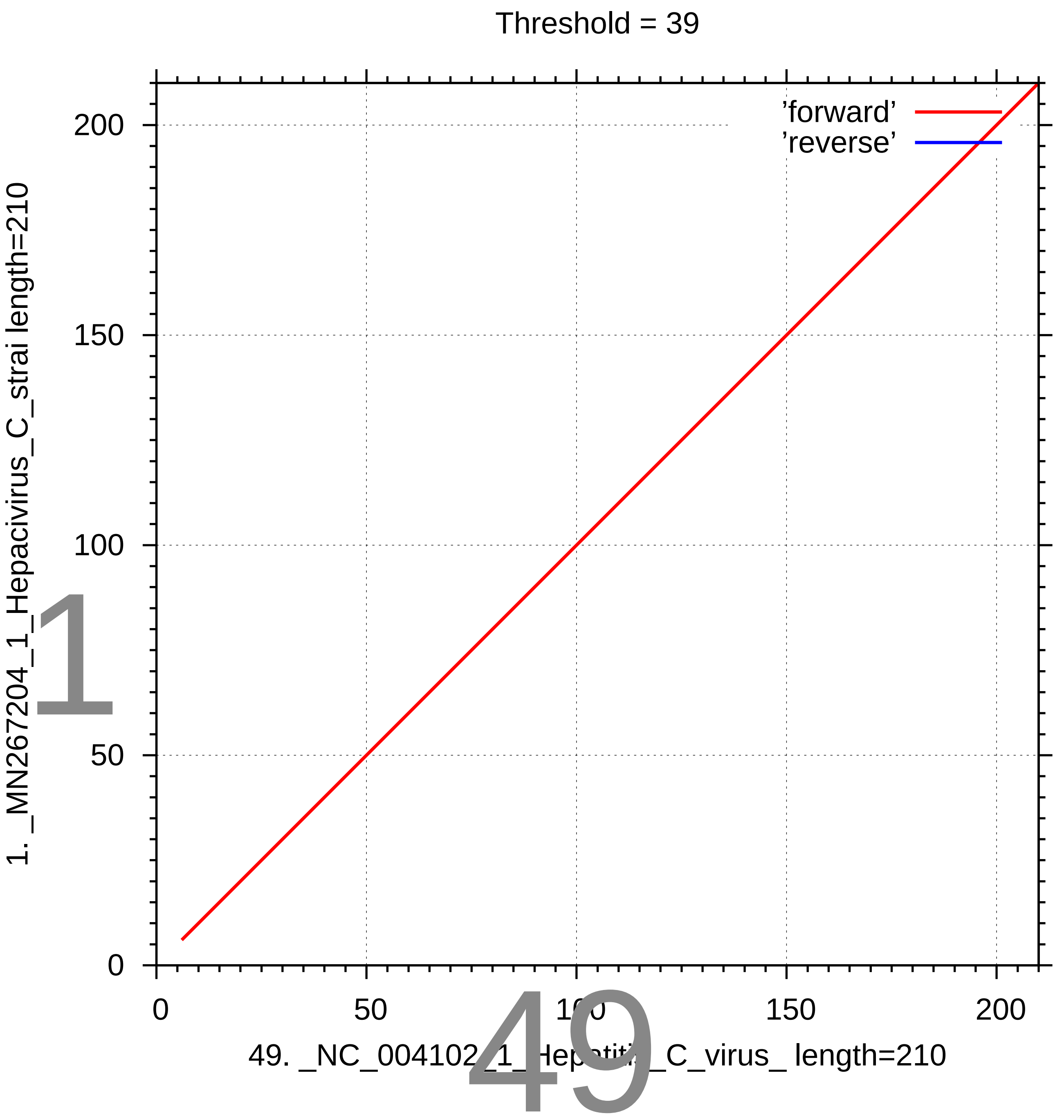

Threshold = 39
'forward'
'reverse'
1. _MN267204_1_Hepacivirus_C_strai length=210
49. _NC_004102
_C_virus_ length=210
0
50
100
150
200
1
49

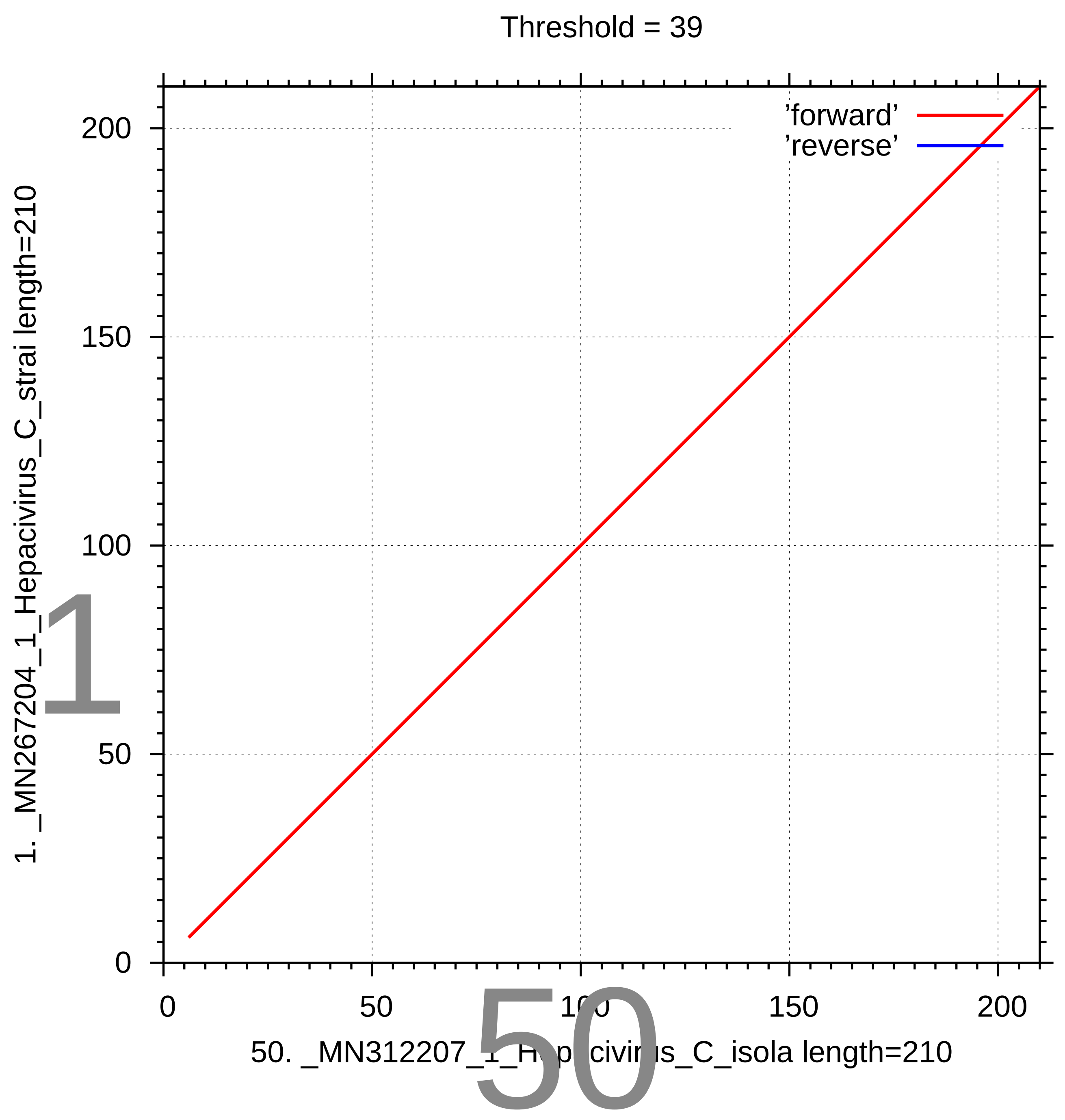
Threshold = 39
'forward'
'reverse'
0
50
100
150
200
1. _MN267204_1_Hepacivirus_C_strai length=210
50. _MN312207_1_Hepacivirus_C_isola length=210
1
50
